# Designed wrinkles for optical encryption and flexible integrated circuit carrier board

**DOI:** 10.1038/s41467-024-50069-7

**Published:** 2024-07-04

**Authors:** Shilong Zhong, Zhaoxiang Zhu, Qizheng Huo, Yubo Long, Li Gong, Zetong Ma, Dingshan Yu, Yi Zhang, Weien Liang, Wei Liu, Cheng Wang, Zhongke Yuan, Yuzhao Yang, Shaolin Lu, Yujie Chen, Zhikun Zheng, Xudong Chen

**Affiliations:** 1 School of Chemical Engineering and Light Industry, Guangdong University of Technology, Guangzhou, China; 2 Jieyang Branch of Chemistry and Chemical Engineering Guangdong Laboratory, Jieyang, Guangdong China; 3 Key Laboratory for Polymeric Composite and Functional Materials of Ministry of Education, School of Chemistry, Sun Yat-sen University, Guangzhou, China; 4 State Key Laboratory of Optoelectronic Materials and Technologies, School of Electronics and Information Technology, Sun Yat-sen University, Guangzhou, China; 5 Unit 66018 of the People’s Liberation Army, Tianjin, China; 6 Instrumental Analysis Research Center, Sun Yat-sen University, Guangzhou, China; 7 School of Materials Science and Engineering, Sun Yat-sen University, Guangzhou, China

**Keywords:** Polymers, Surface patterning

## Abstract

Patterns on polymers usually have different mechanical properties as those of the substrates, causing deformation or distortion and even detachment of the patterns from the polymer substrates. Herein, we present a wrinkling strategy, which utilizes photolithography to define the area of stress distribution by light-induced physical crosslinking of polymers and controls diffusion of residual solvent to redistribute the stress and then offers the same material for patterns as substrate by thermal polymerization, providing uniform wrinkles without worrying about force relaxation. The strategy allows the recording and hiding of up to eight switchable images in one place that can be read by the naked eye without crosstalk, applying the wrinkled polymer for optical anti-counterfeiting. The wrinkled polyimide film was also utilized to act as a substrate for the creation of fine copper circuit by a full-additive process. It generates flexible integrated circuit (IC) carrier board with copper wire density of 400% higher than that of the state-of-the-art in industry while fulfilling the standards for industrialization.

## Abstract (web summary)

Current wrinkling processes for pattern formation cannot be easily controlled, resulting in deformation or detachment of patterns from substrates. Here, Zhong et al. report a wrinkling strategy for creating patterned structures with long-range order and resistant to force relaxation.

## Introduction

Polymers have been widely used as substrates of various micro- or nano-patterns for flexible devices such as electronics and optics^1–4^. Usually, these patterns have different mechanical properties as those of the polymer substrates, resulting in deformation or distortion and even detachment of the patterns from the polymers due to force relaxation^5–7^. In contrast, wrinkle which originates from mechanical instability exists ubiquitous in nature, ranging from nanometer-scale floral structures to millimeter-sized fingerprints and kilometer-scale buckle folds of limestone strata^8–10^. It can change the morphology of substances without interrupting their continuity and integrity, and enable patterned structures with the same mechanical properties as those of substrates, promising great potential to generate patterns on polymer substrates without worrying about force relaxation. Unfortunately, the promise has been greatly hindered by current methods due to uncontrolled formation process^11,12^.

**Fig. 1:**
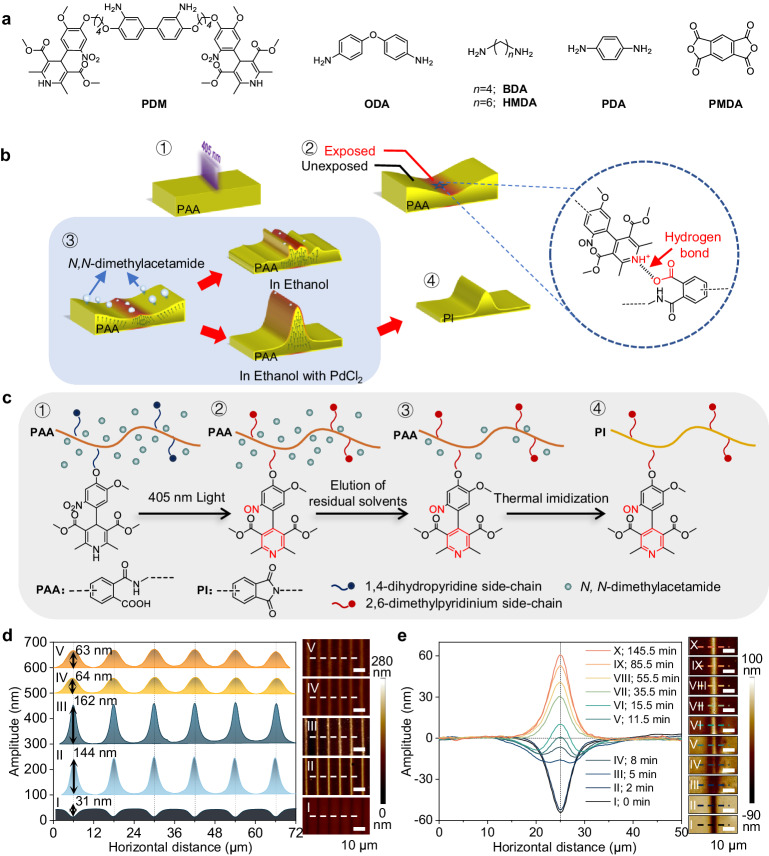
Formation of wrinkled polyimide film. **a** Chemical structure of photosensitive diamine monomer (PDM), 4,4-oxydianiline (ODA), 1,4-butanediamide (BDA), 1,6-hexylenediamine (HMDA), *p*-phenylenediamide (PDA), and pyromellitic dianhydride (PMDA). **b** Schematic for the formation of wrinkled polyimide film. 1. Direct writing of lines with a width of 2 μm on PAA film using 405 nm light. 2. The exposed area turned into dented structures due to the formation of the static and hydrogen bond between 2,6-dimethylpyridinium structures and carboxyl groups of PAA. 3. The dented structures turned into ordered ridge structures instead of random morphology as the residual solvent, i.e., *N*,*N*-dimethylacetamide, was sucked out from the PAA film after development in ethanol containing PdCl_2_. The arrows represent the migration direction of *N*,*N*-dimethylacetamide in the film in ethanol. 4. The winkled PAA film was baked at 300 °C to form wrinkled polyimide. PAA polyamic acid; PI polyimide. **c** Schematic illustration of the structure of PAA with photoactive 1,4-dihydropyridine side-chain (1) before and (2) after photoirradiation, (3) immersion in ethanol containing PdCl_2_, and then (4) imidization at 300 °C. **d** Topology (right) and corresponding height profile (left) of patterned PAA film under photoirradiation (I), and subsequent development in ethanol (II), or development in ethanol containing PdCl_2_ (III) followed by thermal treatment at 200 °C (IV) and 300 °C (V). **e** In situ AFM images (right) of photo-patterned PAA film under ethanol vapor and corresponding height profiles (left). I–X: the profiles of patterned PAA film at different time nodes in unremitting ethanol vapor. Scale bars, 10 μm (right: **d** and **e**).

Skins of animals and plants wrinkle randomly due to uncontrolled loss of water, and the natural drying of a pond leads to partially ordered land wrinkles due to a slow and more controlled dehydration process^13^. Inspired by these natural phenomena, we proposed to generate periodic wrinkles for solution-processed polymer films by controlled removal of residual solvents at designed locations. We first synthesized a photosensitive diamine monomer (PDM) with two 1,4-dihydropyridine side-chains, mixed it with pyromellitic dianhydride (PMDA), 4,4′-oxydianiline (ODA), and 1,6-hexylenediamine (HMDA) with a molar ratio of 1:7:3.6:2.4 in *N*,*N*-dimethylacetamide (boiling point: 165 °C), then curtain-coated the solution onto glass and baked at 60 °C to prepare polyamic acid (PAA) film (Fig. 1a–c, “Methods” and Supplementary Sections 1–3). Direct writing of lines with a width of 2 μm and spacing of 10 μm using 405 nm light led to dents with a depth of 31 nm, which reversed to ridges with amplitude of 144 and 162 nm after immersion for 10 s in ethanol and ethanol containing PdCl_2_, respectively (“I, II, and III” in Fig. 1d). The reversion was further demonstrated by in situ atomic force microscopy (AFM), which showed that a dent with a depth of 60 nm grew gradually from its center to form a ridge with amplitude of 60 nm under ethanol vapor within 2 h (Fig. 1e).

## Results

**Fig. 2:**
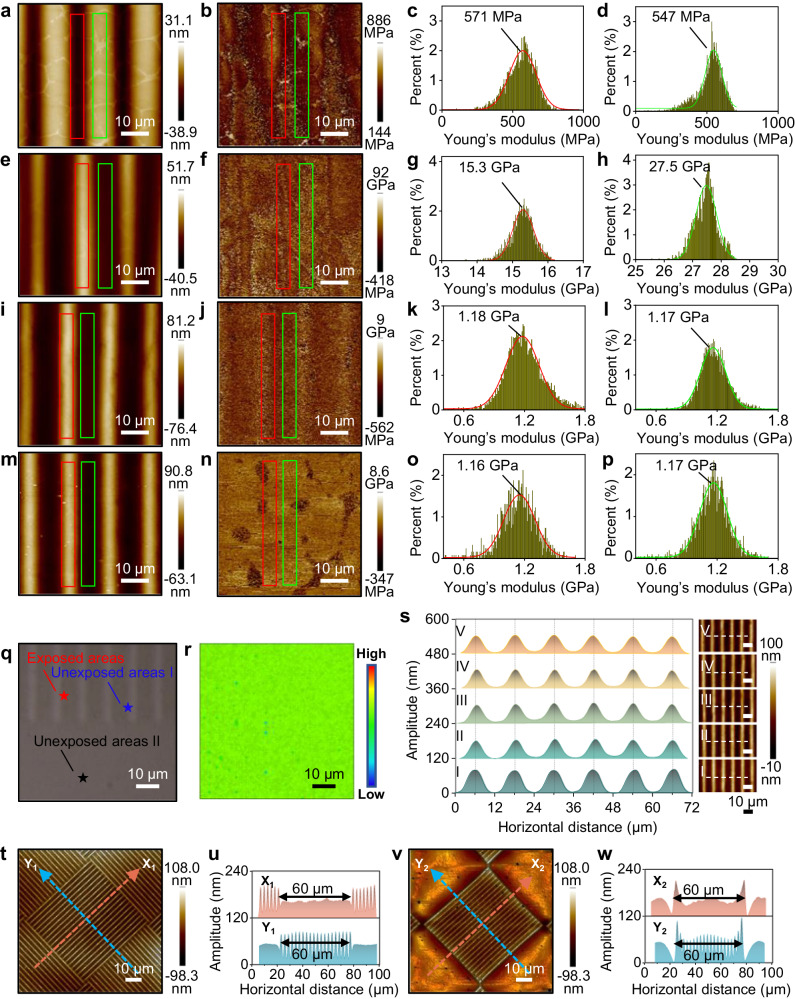
Analysis of wrinkled polyimide film. Morphologies (**a**, **e**, **i**, **m**), and corresponding normalized distribution map (**b**, **f**, **j**, **n**) of Young’s moduli for exposed (**c**, **g**, **k**, **o**) and unexposed area (**d**, **h**, **l**, **p**) of patterned PAA film under photoirradiation (**a**–**d**), subsequent development in ethanol containing PdCl_2_ (**e**–**h**), followed by thermal treatment at 200 °C (**i**–**l**) and 300 °C (**m**–**p**), respectively. Optical image (**q**) and corresponding scanned micro-infrared spectroscopy map (**r**) of wrinkled polyimide film. The scanned micro-infrared spectroscopy map was generated by chemical mapping of selected areas with a scanned frequency range of 873–3856 cm^−1^. **s** Morphologies and corresponding height profile of the wrinkled polyimide film after 1-day immersion in different solvents. I: 1 mol L^−1^ NaOH aqueous solution; II: 1 mol L^−1^ HCl aqueous solution; III: *N,N*-dimethylformamide; IV: acetone; V: H_2_O at 100 °C. **t**, **v** AFM topographic images and **u**, **w** corresponding section profiles of the wrinkled structures in the form of pixels on polyimide film before and after treatment in all the solvents used in (**s**).

The irradiation changed the structure of 1,4-dihydropyridine to 2,6-dimethylpyridinium^14^, which formed static and hydrogen bonds with the carboxyl group of PAA, leading to physical crosslinking^15^ (Fig. 1b, c and Supplementary Figs. 2, 7). It generated the initial force, which caused the volume of the exposed area to shrink and compelled part of the residual *N*,*N*-dimethylacetamide in the exposed area to the unexposed area, offering dented structures (“I” in Figs. 1d, e and 2a). The Young’s modulus at exposed and unexposed areas is 571 and 547 MPa, respectively. When the wrinkled film meets ethanol, a poor solvent for the PAA film but a good solvent for *N*,*N*-dimethylacetamide, which will be sucked out as indicated by a significant increase of Young’s modulus of the whole film at both exposed (15.3 GPa) and unexposed areas (27.5 GPa) (Fig. 2a–h and Supplementary Fig. 8). The suction rate in the unexposed area is higher since no crosslinking blocks the diffusion of residual *N*,*N*-dimethylacetamide and therefore causes higher volume shrinkage, which promotes the movement of PAA chains to the exposed area^16^ (Supplementary Section 3.4). The higher amplitude of the ridges after immersion in ethanol containing PdCl_2_ than in pure ethanol or its vapor might be due to that PdCl_2_ could coordinate with the pyridine ring in the polymer film and increased the density of the surface of the film in exposed area, and therefore increased the osmotic pressure between exposed and exposed area, leading to the higher amplitude of the wrinkled structures (Supplementary Figs. 12–18). Since the stress concentration point is the center of the exposed area, it is stranded out first. Young’s modulus of the exposed area is lower than that of the unexposed area, indicating there was more residual *N*,*N*-dimethylacetamide remained in the exposed area (Fig. 2e–h and Supplementary Fig. 8c, d), which was further confirmed by a 60.5% decrease in amplitude after thermal treatment at 200 °C for 1 h (“III and IV” in Fig. 1d). In contrast, the periodicity, full width at half maximum and orientation remained unchanged. Further increase of the baking temperature to 300 °C to form polyimide (PI) wouldn’t change the morphology of the wrinkled structure (see sample “V” in Fig. 1d), confirming the high resistance of the wrinkles against force relaxation^17^. Taking the unwrinkled area as a reference, the exposed and unexposed areas were complementary in volume, confirming the wrinkled structures were formed due to the movement of the PAA chains (Supplementary Fig. 14). The conclusion was further confirmed by mechanical analysis and micro-infrared spectroscopy as well as its scanning map, which offered the same Young’s modulus and chemical composition for both exposed and unexposed areas, suggesting they were made of the same materials (Fig. 2j–l, n–r and Supplementary Fig. 17). In contrast, patterns with different mechanical properties from those of substrates were obtained typically with contemporary patterning techniques such as photolithography^18^, electron beam lithography^19^, focus ion beam lithography^20^, printing technique^21^, laser patterning^22^, and micro/nano imprinting^23^ as well as previous wrinkling technique based on the difference of mechanical properties along certain direction of materials^24^. Different mechanical relaxation behaviors between the generated patterns and substrates due to variations in constituents or condensed structure resulted in pattern deformation or distortion and even detachment from the underlying substrates^25,26^.

To check the homogeneity of the polyimide wrinkles over large areas, spin-coated PAA film on 4-inch silicon wafer was utilized. Nine representative areas either at the center or corner of the film were selected to elucidate their morphology by AFM, which showed no significant change in both amplitude and width of the formed wrinkles, further confirming the reliability and reproducibility of the developed wrinkling strategy (Supplementary Figs. 19–22).

We then studied the chemical stability of the wrinkles by 24 h immersion in boiling water, 1 mol L^−1^ hydrogen chloride aqueous solution, 1 mol L^−1^ sodium hydroxide aqueous solution, and *N*,*N*-dimethylformamide. No obvious change of morphology was observed, indicating the wrinkles were stable under these conditions (Fig. 2s). Such stability is unreached for general polymers with periodic microstructures^27^. We went further to investigate the stability of the edge structures of the wrinkles by selecting a square with straight lines surrounded by squares with straight lines in orthogonal directions (Fig. 2t–w). No obvious alternation of the lines after immersion in the solvent mentioned above, further confirming the chemical stability of the fabricated wrinkles.

Next, we investigated the effect of baking temperature, exposure time and flexibility of the PAA film on the amplitude of the wrinkled structures (Supplementary Section 4.2). It increased with exposure time and reached a maximum at 180 ms, and then decreased and fluctuated thereafter. The exposed areas with physical crosslinking structures had a lower residual solvent diffusion rate than that at unexposed areas under the suction of ethanol, leading to force difference between these two areas and induced the formation of wrinkles. In theory, a higher degree of crosslinking formed with longer exposure time would bring higher force difference during the residual solvent diffusion process, which would lead to the formation of wrinkles with higher amplitude. On the other hand, higher degree of crosslinking could also block the directional migration of polymer chains and thus hindering the formation of wrinkles. Therefore, it is important to choose a suitable exposure time (180 ms) to balance the residual solvent diffusion rate and the degree of crosslinking. In addition, without the existence of HMDA as a soft linker, or when it was replaced with rigid diamines such as 1,4-butanediamide (BDA) and *p*-phenylenediamide (PDA), the lower amplitude was obtained. The amplitude reached the maximum when the molar amount of HMDA reached 34% of PMDA. Moreover, the ordered wrinkles can also be formed on the film after the immersion for 10 s in ethanol containing Cu(NO_3_)_2_ or methanol containing PdCl_2_ (Supplementary Figs. 28, 35).

The periodic wrinkles exhibited iridescence due to first-order light diffraction whose wavelength (*λ*) could be described by *λ* = *D* (sin *β* − sin *α*), where *D* is the periodicity of the wrinkles, *α*/*β* is the angles between incidence/observation light and surface normal^28^ (Supplementary Figs. 13b, 19 and Supplementary Section 5.1). Both wrinkled PAA and polyimide showed good monochromaticity over micrometer-sized areas, further confirming their homogeneity. Over larger areas, such as wrinkles on the 4-inch wafer showed a gradual transition of the color due to the gradual change of *β* (Supplementary Fig. 19c). Besides the straight lines, dot arrays were also generated. The wrinkled patterns fabricated by the combination of line and dot arrays on film also showed angle-dependent structural color on the viewing angle *β*, and monochromaticity of both patterns was observed under the corresponding viewing angle (Supplementary Fig. 32 and Supplementary Movie 1).

To gain an insight of the flexibility of the polyimide wrinkles, a polyimide wrinkle of Guangzhou Tower was selected: the polyimide film was detached from its supporting glass, picked up by tweezers then bent up and down (Supplementary Fig. 33). No obvious difference of the diffraction of the pattern was observed before and after the treatment, confirming the high flexibility and mechanical robustness of the polyimide wrinkle.

**Fig. 3:**
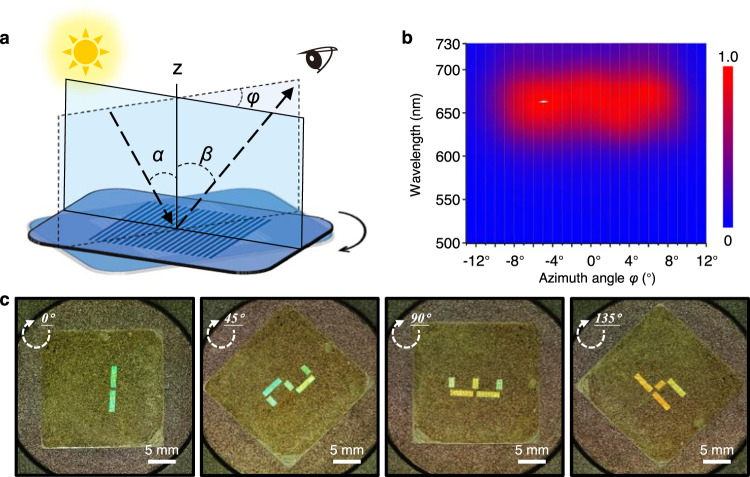
Rational design of wrinkles for optical encryption. **a** The schematic illustration of azimuthal angle *φ* changes of viewing position followed only rotating sample in-plane. The *α* and *β* located on either side of normal line z*,* respectively, represent the incident angle and diffractive angle. **b** The statistical reflection spectra of the wrinkling area for different azimuthal angle *φ* with *α* = 0° and *β* = 12.7°. Color scale bar, reflectance value. **c** Optical images of the wrinkled PAA film at different rotation angles *φ* (0°, 45°, 90°, and 135°).

Interestingly, light observed by the naked eye could be controlled by regulating the azimuthal angle (*φ*) of the plane of incidence and observation light with that of surface normal and direction of the wrinkles by rotation^29^ (Fig. 3, Supplementary Fig. 34 and Supplementary Section 5.2). The wrinkles were red with maximum light intensity at ~670 nm and *α* = 0° and *β* = 12.7°. The light intensity remained constant at |*φ*| ≦ 6°, decreased significantly with further increase of the |*φ*|, and no patterns could be visualized due to the disappearance of the diffracted light at |*φ*| ≧ 12°.

We then created switchable images that could be read by the naked eye with the wrinkling strategy and multiplexing technique, a widely used technique in creating multiple images at one position in high-capacity optical information storage^30^. Since a wrinkled image disappeared in the eye with a rotation angle (*φ*) of ±12° and only a reversed image was observed for angles higher than 180° (Fig. 3b), eight (180°/24°) switchable images could be visualized at one position at maximum. Both continuous switching of four images (numbers 1–4) and eight images (numbers 1–8) were demonstrated without visible crosstalk (Supplementary Figs. 34–38 and Supplementary Movies 2, 3). In contrast, contemporary techniques such as orbital angular momentum holography can switch two images without crosstalk and four images in maximum at one position with obvious crosstalk^31^.

Next, we tuned the coefficient thermal expansion of the polyimide wrinkles by replacing ODA with PDA, and reached the same value (17.8 × 10^−6^/°C) as that of copper with a molar ratio for PDM:PMDA:PDA of 1:8:7 (Supplementary Fig. 39a). The consistency allows the application of the wrinkled polyimide as substrate for the construction of fine copper circuit to make flexible IC carrier board since detachment of copper from substrate due to temperature change could then be avoided at maximum.

IC carrier board is the platform on which components of different devices, ICs, and antenna were built. It uses either flexible or hard board. The flexible IC board is composed of a flexible polymer substrate (mainly polyimide and polyester) and fine copper circuits^32^. At present, polyimide film has the best comprehensive performance due to its excellent high-temperature resistance, mechanical properties, and chemical stability.

The rapid development of ICs has promoted the development of IC carrier board in the direction of high-density, high integration, packaging, miniaturization, and multi-layer, especially the development of line width and line spacing of conductive lines to high-density and high-precision^33^. In industry, copper circuits on IC carrier board are typically produced by a subtractive process, which wastes high amounts of copper, contaminates the environment, and increases the cost^34^. In the process, photoresists are needed for the construction of circuits and removed afterward. Noteworthy, an adhesive interposer is needed to bond copper foil to substrate which easily leads to adhesion failure due to the oxidative degradation, moisture absorption, and mismatch of its thermal expansion coefficient with that of copper^35^.

**Fig. 4:**
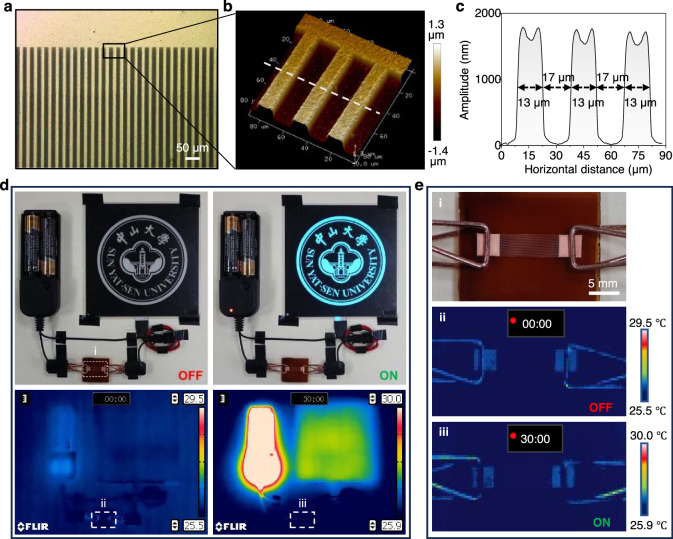
Copper circuit on wrinkled polyimide film. **a** Optical microscopy image of a copper circuit on wrinkled polyimide film. The copper lines are connected to a square copper foil with a side length of 4 mm. **b** Three-dimensional AFM image and **c** corresponding height profile of the copper lines. **d** Photographic (upper) and infrared camera (lower) images of a conductive circuit composed of a power supply, a copper circuit, and a customized luminescent plate before (left) and after (right) 30 min electrification. Except for the power supply and the customized luminescent plate, no visible infrared image of the copper circuit was obtained indicating it had higher conductivity and did not contain broken copper lines which would generate heat. **e** Optical image (i) of the copper circuit, which is contacted in the conductive circuit by two copper clips. Infrared camera images of the copper circuit before (ii) and after (iii) 30 min electrification.

Here, copper was directly deposited onto wrinkled polyimide film without the use of photoresist and adhesive interposer by a full-additive process involving electroless deposition with Pd(0) as nucleation agent (Fig. 4 and Supplementary Section 7), which came from thermally reduced Pd^2+^ that coordinated to nitrogen atom in the pyridine ring at the exposed area of PAA during immersion in ethanol containing PdCl_2_^36^. We produced a copper circuit composed of contacts and interconnects on polyimide film with a thickness of ~12 μm. The lines are sharp, continuous, and uniform over centimeter-sized areas, and their width, thickness, and interline spacing are ~13, 1.8, and 17 μm, respectively (Fig. 4c and Supplementary Section 7.3). The line density is more than 400% higher than that of the state-of-the-art in the industry (both line width and spacing are 76.2 μm)^37^. The American Society for Testing and Materials standard D3359 tape test method was then applied to test the adhesion stability of the copper patterns on the polyimide film, and the highest grade (5B) was reached (Supplementary Movie 4). The electrical resistance of the copper circuit remained unchanged after 1000 times cyclic bending with a curvature radius of 0.5 mm as well as stretching with a cyclic strain of 3%, indicating their high flexibility and mechanical robustness (Supplementary Section 7.4). The copper circuit was then integrated into a closed circuitry to light an electroluminescent device for more than 30 min without obvious heating effects with a current density of 3.95 × 10^7^ A·m^−2^, further confirming the high conductivity and reliability of the copper circuit (Fig. 4d, e and Supplementary Movies 5, 6). The relative dielectric constant of the polyimide film is 3.3 (Supplementary Fig. 39b), which meets the requirement of materials for IC carrier board (3.0–3.5) in general environments. Note that, all the parameters of the IC carrier board meet or go beyond the industry standards for commercialization (Supplementary Table 5).

## Discussion

In summary, we presented a wrinkling strategy for creating patterned structures with long-range order over wafer scale on polymer substrates. Both the light-induced physical crosslinking at defined areas and the following diffusion of residual solvents redistributed the stress of polymer film to induce ordered wrinkling structures. We demonstrated its implementation for the unprecedented generation of microstructures on polymers without worrying about force relaxation. We illustrated the application of these structures for high-capacity information encryption and optical anti-counterfeiting by generating eight switchable images in one place without crosstalk. We went further to show the wrinkled polyimide film could act as a substrate for the creation of fine copper circuits by a full-additive process. Its application was expended to generate a flexible IC carrier board with copper wire density of 400% higher than that of the state-of-the-art in the industry while fulfilling the standards for industrialization.

## Methods

### Synthesis of photosensitive PAA polymers

#### General procedure A

The photosensitive PAA polymers are synthesized by the polycondensation method. Briefly, all diamine monomers are dissolved in DMAc at 5 °C under dark. The solution is stirred for 30 min at 5 °C, and then the PMDA is added into the solution in batches and stirred for 12 h to afford photosensitive PAA solution. The five kinds of available diamine monomers (PDM, ODA, HMDA, BDA, and PDA) are employed to have a condensation polymerization with PMDA. Thus, a series of PAA films, including PDM–PMDA–ODA (PPO), PDM–PMDA–ODA–HMDA (PPOH), PDM–PMDA–ODA–BDA (PPOB), PDM–PMDA–ODA–PDA (PPOP), PDM–PMDA–PDA (PPP), and PDM–PMDA are synthesized.

PDM–PMDA–ODA (PPO): PDA (1.11 g, 1 mmol), PMDA (1.53 g, 7 mmol), ODA (1.20 g, 6 mmol), and 34.56 g DMAc are used to obtain PPO solution under general procedure A.

PDM–PMDA–ODA–HMDA-1 (PPOH-1): PDM (1.11 g, 1 mmol), PMDA (1.53 g, 7 mmol), ODA (1.06 g, 5.28 mmol), HMDA (0.08 g, 0.72 mmol), and 34.02 g DMAc are used to obtain PPOH-1 solution under general procedure A.

PDM–PMDA–ODA–HMDA-2 (PPOH-2): PDM (1.11 g, 1 mmol), PMDA (1.53 g, 7 mmol), ODA (0.90 g, 4.5 mmol), HMDA (0.17 g, 1.5 mmol), and 33.39 g DMAc are used to obtain PPOH-2 solution under general procedure A.

PDM–PMDA–ODA–HMDA-3 (PPOH-3): PDM (1.11 g, 1 mmol), PMDA (1.53 g, 7 mmol), ODA (0.72 g, 3.6 mmol), HMDA (0.28 g, 2.4 mmol), and 32.76 g DMAc are used to obtain PPOH-3 solution under general procedure A.

PDM–PMDA–ODA–HMDA-4 (PPOH-4): PDM (1.11 g, 1 mmol), PMDA (1.53 g, 7 mmol), ODA (0.54 g, 2.7 mmol), HMDA (0.38 g, 3.3 mmol), and 32.04 g DMAc are used to obtain PPOH-4 solution under general procedure A.

PDM–PMDA–ODA–HMDA-5 (PPOH-5): PDM (1.11 g, 1 mmol), PMDA (1.53 g, 7 mmol), ODA (0.36 g, 1.8 mmol), HMDA (0.49 g, 4.2 mmol), and 31.41 g DMAc are used to obtain PPOH-5 solution under general procedure A.

PDM–PMDA–ODA–BDA (PPOB): PDM (1.11 g, 1 mmol), PMDA (1.53 g, 7 mmol), ODA (0.72 g, 3.6 mmol), BDA (0.21 g, 2.4 mmol), and 32.13 g DMAc are used to obtain PPOB solution under general procedure A.

PDM–PMDA–ODA–PDA (PPOP): PDM (1.11 g, 1 mmol), PMDA (1.53 g, 7 mmol), ODA (0.72 g, 3.6 mmol), PDA (0.26 g, 2.4 mmol), and 32.58 g DMAc are used to obtain PPOP solution under general procedure A.

PDM–PMDA–PDA-1 (PPP-1): PDM (1.11 g, 1 mmol), PMDA (1.53 g, 7 mmol), PDA (0.65 g, 6 mmol), and 29.61 g DMAc are used to obtain PPP-1 solution under general procedure A.

PDM–PMDA–PDA-2 (PPP-2): PDM (1.11 g, 1 mmol), PMDA (1.74 g, 8 mmol), PDA (0.76 g, 7 mmol), and 32.49 g DMAc are used to obtain PPP-2 solution under general procedure A.

PDM–PMDA–PDA-3 (PPP-3): PDM (1.11 g, 1 mmol), PMDA (1.96 g, 9 mmol), PDA (0.86 g, 8 mmol), and 35.37 g DMAc are used to obtain PPP-3 solution under general procedure A.

PDM–PMDA–PDA-4 (PPP-4): PDM (1.11 g, 1 mmol), PMDA (2.18 g, 10 mmol), PDA (0.97 g, 9 mmol), and 38.34 g DMAc are used to obtain PPP-4 solution under general procedure A.

PDM–PMDA–PDA-5 (PPP-5): PDM (1.11 g, 1 mmol), PMDA (2.40 g, 11 mmol), PDA (1.08 g, 10 mmol), and 41.31 g DMAc are used to obtain PPP-5 solution under general procedure A.

PDM–PMDA: PDM (1.11 g, 1 mmol), PMDA (0.22 g, 1 mmol), and 11.97 g DMAc are used to obtain PDM–PMDA solution under general procedure A.

### Formation of wrinkles on photosensitive PAA films

First, in the dark, the solutions of PAA polymers are coated on the clean cover-glasses (2.5 × 2.5 cm) or 4-inch silicon wafer in fixed volume (100 μL for cover-glasses and 1 mL for silicon wafer), and followed by baking in a vacuum oven at 60 °C for 2 h, then obtained the original photosensitive films. The preparation process schematic of the grating wrinkles on PAA films is shown in Supplementary Fig. 10. A PAA film on the substrate is fixed on the Maskless Lithography Machine (µPG 501, Heidelberg) platform, then the grating layout is exposed on the film surface by 405-nm light and the original wrinkles formed immediately. After exposed, the film is immersed into ethanol solution of palladium chloride (PdCl_2_/ethanol, 0.1 mmol L^−1^) for 10 s and then dried with hot wind to gain wrinkling structures on the PAA film.

### Preparation of copper circuit

Two special patterns of electrode shape are designed: two kinds of periodic lines (condition 1, 10-mm length, 10-μm width, 90-μm spacing; condition 2, 10-mm length, 10-μm width, 20-μm spacing) are distributed between two squares with a side length of 4 mm, and each line is connected to the square closely. Two patterns are exposed on the PAA films through the maskless lithography system, respectively, and the exposed time is set as 180 ms. Then the exposed films are immersed in the PdCl_2_/ethanol solution for 10 s, and the sample surface is rinsed repeatedly with anhydrous ethanol. The PAA films which surface adsorbed PdCl_2_ are placed in a vacuum oven to experience a programmed heating procedure and then transformed into polyimide films. The polyimide films are treated with the typical electroless plating process at room temperature to obtain the final copper circuit samples.

### Supplementary information

Supplementary Information

Description of Supplementary Information

Supplementary Movie 1

Supplementary Movie 2

Supplementary Movie 3

Supplementary Movie 4

Supplementary Movie 5

Supplementary Movie 6

### Source data

Source data

## Data Availability

All data supporting the findings of this study are available within the paper and its Supplementary Information files.   Source data are provided with this paper.
