# Supplementary material for: Designed wrinkles for optical encryption and flexible integrated circuit carrier board
Source: Nat Commun. 2024 Jul 4;15:5616. doi: 10.1038/s41467-024-50069-7 (PMC11224375; doi:10.1038/s41467-024-50069-7)
Supplement: Supplementary file 1 — Supplementary Information [file 41467_2024_50069_MOESM1_ESM.pdf]

Supplementary Information for "Designed Wrinkles for Optical Encryption and Flexible Integrated Circuit Carrier Board"

Shilong Zhong, Zhaoxiang Zhu, Qizheng Huo, Yubo Long, Li Gong, Zetong Ma, Dingshan Yu, Yi Zhang, Weien Liang, Wei Liu, Cheng Wang, Zhongke Yuan, Yuzhao Yang, Shaolin Lu, Yujie Chen, Zhikun Zheng, Xudong Chen

Table of Contents

Materials and Methods

Section 1: Synthesis procedures

Section 1.1 Synthesis of photosensitive diamine monomer

Section 1.2 Synthesis of photosensitive polyamic acid polymers

Section 2: Structural properties of photosensitive diamine monomer

Section 2.1 Photosensitive properties analysis

Section 2.2 Thermal stability analysis

Section 3: Structural properties of photosensitive polyamic acid

Section 3.1 Nuclear magnetic resonance spectroscopic analysis

Section 3.2 Fourier transform infrared spectroscopic analysis

Section 3.3 Young's modulus determined by AFM

Section 3.4 Residual solvent absorption and desorption analysis

Section 4: The formation of wrinkles on photosensitive polyamic acid films

Section 4.1 The fabrication of uniform wrinkles in a large area

Section 4.2 Controllable adjustment of wrinkles morphology

Section 5: Optical properties of wrinkles on photosensitive polyamic acid films

Section 5.1 Diffraction simulations of wrinkles under out-of-plane rotation

Section 5.2 Diffraction simulations of wrinkles under in-plane rotation

Section 6: Patterned wrinkling photosensitive polyamic acid film

Section 6.1 Dot matrix patterns and wrinkles patterns

Section 6.2 Patterning four-plex wrinkles

Section 6.3 Patterning eight-plex wrinkles

Section 7: Constructing copper circuit on the wrinkles template

Section 7.1 Coefficient thermal expansion and dielectric properties analysis

Section 7.2 The characterization analysis of fine copper lines with a 13-μm width and an 87-μm spacing

Section 7.3 The characterization analysis of fine copper lines with a 13-μm width and a 17-μm spacing

Section 7.4 Mechanical stability of copper circuit

Section 8: Supplementary animation demonstration

Section 8.1 Supplementary Movie 1

Section 8.2 Supplementary Movie 2

Section 8.3 Supplementary Movie 3

Section 8.4 Supplementary Movie 4

Section 8.5 Supplementary Movie 5

Section 8.6 Supplementary Movie 6

Supplementary Methods

Materials.

All analytical pure solvents and reagents mentioned in this paper, which have enough purity to be used directly unless descript specified, are purchased from Macklin (Macklin Biochemical Co., Ltd., Shanghai, China).

Characterization.

A Nuclear Magnetic Resonance Spectroscopy (CyroProbe TCI, Bruker) is used to confirm the chemical structures of Compounds ii-v and PPOH-3 (see in Section 1.2). Chloroform-*d* ($CDCl_3$), dimethylsulfoxide-d_6 (DMSO-d_6) and *N*, *N*-dimethylformamide-d_7 (DMF-d_7) are used as a solvent to dissolve products.

A Liquid Chromatography-Triple Quadrupole Mass Spectrometry (TSQ Endura, Thermo Fisher) is employed to identify the molecular weight of Compounds ii, iii, and iv. A Matrix-Assisted Laser Desorption / Ionization Time of Flight Mass Spectrometry (Ultrafle Xtreme, Bruker) is employed to identify the molecular weight of Compound v. Compounds ii-v are needed to dissolve in methyl alcohol before testing.

A Thermo Gravimetric Analysis (TG209F1 Libra, NETZSCH) is used to analyze the thermal stability of Compounds i, ii, v and PPOH-3. It operates in air or nitrogen atmosphere and the testing temperature ranges from 25 ℃ to 900 ℃ with a heating rate of 5 ℃ min^{-1}.

An Ultraviolet and Visible (UV-vis) Spectrophotometer (Lambda 950, Perkin Elmer) is used to research the optical absorption properties of the photosensitive materials of Compound ii to ensure the 405 nm light (Maskless Lithography Machine) satisfies the requirement of subsequent exposing. And this equipment is used to detect the gelation process of polyamic acid solution mixed with ethanol under transmissive mode.

A Fluorospectro Photometer (F-4500, Hitachi) is employed to detect the fluorescence characteristic of the ethanol solutions of Compound ii (1 $mmol{\cdot}L^{-1}$) that is irradiated with different durations by 405 nm light. The excitation wavelength (430 nm) is generated by a 30 W light source.

A Stereoscopic Microscope (WSZ0745TB, Guangzhou micro domain optical instrument Co. Ltd.) with industrial digital camera (ICX252AQ, SONY) is employed to capture the details of wrinkled images with structural color. Samples on the objective table are illuminated by oblique incidence white light generated with a 15 W light-emitting diode (BT-TCL96, Microvision China) source.

An Ultra-depth Three-dimensional Microscope (KEYENCE VHX-1000C, Keyence) is used to capture microstructure images including wrinkled patterns and copper circuit. Samples on the objective table are illuminated by a 25 W halogen lamp at the top.

An Atomic Force Microscope (AFM, Dimension Fastscan, Bruker) is used to monitor the formation process of wrinkles and analyze the profiles and mechanical properties of the wrinkles on films. Both the morphology and Young’s modulus of the

photosensitive polyamic acid film surface in an ethanol vapor atmosphere are analyzed under in-situ AFM method. A self-made device consisting of a petri dish and a small plastic block is used to keep the ethanol vapor atmosphere (Supplementary Fig.1), the small plastic block is fixed on the center of the petri dish through hot melt adhesive. PPOH-3 film sample is fixed on the top of the small plastic block, and a few milliliters of anhydrous ethanol is added to the device, where the ethanol liquid level is below the top surface of the plastic block, so the ethanol vapor atmosphere can be realized in a few minutes later as a cover on the device. The duration of the sample in the steam atmosphere is recorded by the method of accumulative timing, which is automatically stopped when the cover of the device is removed, and then the surface morphology and Young’s modulus evolution of the polyamic acid (PAA) film are characterized and the relevant data are collected.

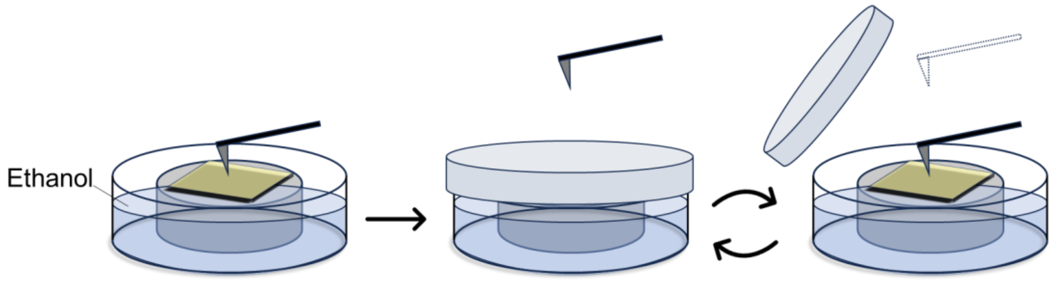

Supplementary Figure 1. In-situ AFM measurement process schematic of grating wrinkles on PAA films.

A Digital Movie Camera (FDR-AX60, SONY) is employed to take photographs and record dynamic displays of samples. Before filming, samples are illuminated with a white light source, the capture direction of the camera is adjustable with the rotation of the tripod head perpendicular to the wrinkling structures.

A Fiber Spectrometer (MAYA2000-Pro, Ocean Optics) with a 215-2500 nm source (DH-2000, Ocean Optics) is used to obtained reflectance spectra. All reflectance spectra are normalized compared with naked cover glass.

A Spectrometer (Nicolet Nexus 670, Thermo Electron Co., Ltd.) is used to record the temperature-dependent Fourier transform infrared (FTIR) spectra of polyamic acid film (PDM-PMDA, see in Section 1.2). The film is sandwiched between two KBr plates, then the absorption spectra are recorded from 30 °C to 150 °C with a heating rate of 5 °C min^{-1}.

A Micro-IR Bruker VERTEX 70 instrument coupled Hyperion 2000 microscope is used to characterize structural changes of the PDM-PMDA film with different exposed times and the wrinkles on the PPOH-3 film after thermal imidization.

A Scanning Electron Microscope (SEM, Regulus 8230, Hitachi) combined with a Energy Dispersive Spectrometer (EDS) is used to observe the chemical and morphological characterization of samples. It is operated at the accelerating voltage between 2 keV and 5 keV. The samples are sputtered with Vacuum Sputtering Equipment (SC7620, Quorum) in advance.

An Infrared Camera (T530, FLIR) is used to capture the thermal effect of the flexible printed circuit sample. The flexible copper circuit sample (see in Supplementary Figure 26c, 13-μm width and 87-μm spacing) is connected to a conductive circuit composed of a power supply (3 V, 100 mA) and a customized electro-luminescent plate.

An Electrochemical Workstation (CHI 760E, Shanghai Chenhua Instrument Co. Ltd.) is used to test the continuity of the copper circuit under repeated bending at a bending radius of 0.5 mm. Cyclic linear voltage sweeps mode is adopted with a voltage sweep range from 0 V to 3 V.

A high-frequency dielectric properties test system (Keysight P5003A, USA) is used to characterize the dielectric properties of polyimide samples. The measurement method is split post, and the area of the test electrodes is 13.56 cm^2, the frequency is set as 10 GHz, and the environment temperature is 25 ℃.

A Static Thermomechanical Analyzer (TMAQ400, TA) is used to measure the linear coefficient thermal expansion of the polyimide samples with a heating rate of 2 ℃ min^{-1} from 30 ℃ to 280 ℃. The thickness of film is ~10 μm by a coating machine, then it is cut into rectangular shapes with 20-mm length and 5-mm width.

A Direct Current Resistance Measurer (TH2512B+, TongHui) is used to measure the resistance of the copper circuit. The flexible copper circuit sample with 13-μm line width and 87-μm line spacing is bent to a state of 0.5-mm curvature radius and then recovery to the original statement and then begins the resistance test.

Section 1: Synthesis procedures

Section 1.1 Synthesis of photosensitive diamine monomer (PDM)

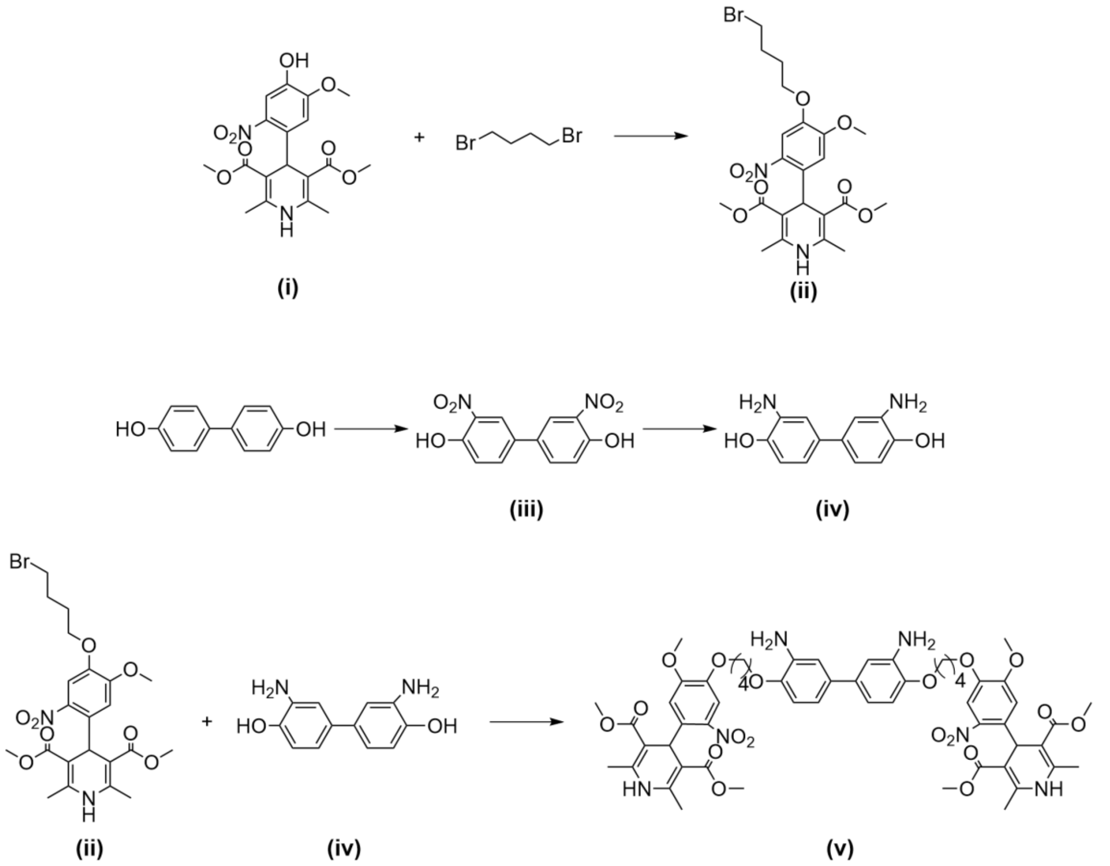

Compound i: Dimethyl-4-(4-hydroxy-5-methoxy-2-nitro-phenyl)-2,6-dimethyl-1,4-dihydropyridine-3,5-dicarboxylate. It is synthesized by the method had been reported in our previous work[1].

Compound ii: 4-[4-(4-Bromo-butoxy)-5-methoxy-2-nitro-phenyl]-2,6-dimethyl-1,4-dih-ydro-pyridine-3,5-dicar-boxylic acid dimethyl ester. Under dark environment, a mixture of Compound i (27.47 g, 0.07 mol), 1,4-dibromobutane (22.67 g, 0.11 mol), and cesium carbonate (34.21 g, 0.11 mol) are dissolved in 150-mL tetrahydrofuran then stirred at 60 °C for 15 hours under the N_2 atmosphere. After filtering, the filtrate solvent is further evaporated to leave the crude product. The product isolated as yellow powder is obtained after recrystallization from ethyl

alcohol. Yield: 89.9%. **^{1}H NMR ($CDCl_3$, 500 MHz):** δ 7.28 (s, 1H), 6.89 (s, 1H), 5.84 (s, 1H), 5.68 (s, 1H), 4.04 (t, J = 5.9 Hz, 2H), 3.80 (s, 3H), 3.59 (s, 6H), 3.48 (t, J = 6.5 Hz, 2H), 2.34 (s, 6H), 2.10 - 2.02 (m, 2H), 1.99 (dt, J = 13.8, 6.7 Hz, 2H). **^{13}C NMR ($CDCl_3$, 126 MHz):** δ 167.60, 153.02, 146.56, 144.52, 140.70, 136.62, 112.57, 108.75, 104.18, 68.32, 56.05, 51.16, 34.51, 33.26, 29.42, 27.67, 19.55. **LCMS (cESI):** calcd for $[M + Na]^+$ $[C_{22}H_{27}N_2O_8BrNa]^+$, m / z: 550.100, found: 550.020.

Compound iii: 3, 3'-Dinitrobiphenyl-4, 4'-dihydroxybiphenyl. Concentrated nitric acid (34 mL) is added into the round-bottom flask and cooled to 0 °C, then 4, 4'-dihydroxybiphenyl (4.66 g, 0.025 mol) is added into the flask in batches. The mixture is stirred at 0 °C for 4 hours before it warmed up to room temperature. The yellow solid is obtained by filtration and washed several times with deionized water. The product isolated as yellow powder is obtained after recrystallization from toluene / *N*, *N*-dimethylformamide (DMF). Yield: 98.5%. **^{1}H NMR (DMF-d_7, 500M):** δ 11.33 (s, 2H), 8.31 (d, J = 2.4 Hz, 2H), 8.03 (s, 2H), 7.34 (d, J = 8.7 Hz, 2H). **^{13}C NMR (DMF-d_7,126 MHz):** δ 152.32, 137.25, 133.51, 130.02, 122.74, 120.09. **LCMS (cESI):** calcd for $[M - H]^+$ $[C_{12}H_7N_2O_6]^+$, m / z: 275.040, found: 274.590.

Compound iv: 3,3'-Diaminobiphenyl-4,4'-dihydroxybiphenyl[2]. A solution of Compound iii (3.04 g, 0.011 mol), hydrazine monohydrate (9.0 mL, 0.18 mol) and Pd / C (0.3 g) are dissolved in 25-mL absolute ethanol then stirred at 85 °C for 4 hours under the N_2 atmosphere. After filtering, the crude gray solid is obtained. Afterward

the solid is dissolved into hot DMF and then filtered through the Celite® to remove the Pd / C catalyst. The filtrate is dropped into cold water and the precipitate are collected. The precipitate is dried in the oven to obtain the final gray product. Yield: 99.2%. **^{1}H NMR (DMSO-*d_6*, 500 MHz):** δ 8.90 (s, 2H), 6.75 (d, J = 2.1 Hz, 2H), 6.63 (d, J = 8.1 Hz, 2H), 6.53 (dd, J = 8.1, 2.1 Hz, 2H), 4.51 (s, 4H). **^{13}C NMR (DMSO-*d_6*, 126 MHz):** δ 143.36, 136.98, 133.29, 114.97, 114.64, 112.83. **LCMS (cESI):** calcd for $[M + H]^+$ $[C_{12}H_{13}N_2O_2]^+$, m / z: 217.090, found: 217.040.

Compound v / PDM: Tetramethyl 4,4'-(((((3,3'–diamino-[1,1'-biphenyl]-4,4'-diyl) bis(oxy)) -bis- (butane-4,1-diyl))bis(oxy))bis(5-methoxy-2-nitro-4,1-phenylene))bis(2,6-dimethyl-1,4-dihydropyridine-3,5-dicarboxylate). Under dark environment, a mixture of Compound ii (6.96 g, 0.013 mol), Compound iv (1.3 g, 0.006 mol) and cesium carbonate (4.89 g, 0.015 mol) are dissolved in dry DMF (35 mL) and stirred at 80 °C for 15 hours under the N_2 atmosphere. After filtering, the filtrate is dropped into cold water and the precipitate is collected. The crude product is purified by column chromatography using CH_2Cl_2 / CH_3OH (= 60 / 1 by volume) as an eluent to obtain the pure Compound v (PDM). Yield: 74.5%. **^{1}H NMR ($CDCl_3$, 500 MHz):** δ 7.29 (s, 2H), 6.88 (s, 2H), 6.87 (d, J = 1.8 Hz, 2H), 6.84 (d, J = 8.6 Hz, 2H), 6.77 (d, J = 8.3 Hz, 2H), 5.83 (s, 2H), 5.28 (d, J = 0.9 Hz, 1H), 4.09 (d, J = 5.5 Hz, 8H), 3.75 (s, 6H), 3.57 (s, 12H), 2.31 (s, 12H), 2.07 - 1.95 (m, 8H). **^{13}C NMR ($CDCl_3$, 126 MHz):** δ 167.68, 153.01, 146.55, 145.88, 144.78, 140.60, 136.56, 135.82, 134.35, 117.05, 113.90, 112.45, 111.61, 108.58, 103.97, 68.93, 67.84, 55.98,

51.12, 34.45, 26.19, 25.78, 19.40. **FTMS (cESI):** calcd for $[M + H]^+$ $[C_{56}H_{65}N_6O_{18}]^+$, m / z: 1110.430, found: 1110.440.

Section 1.2 Synthesis of photosensitive polyamic acid polymers

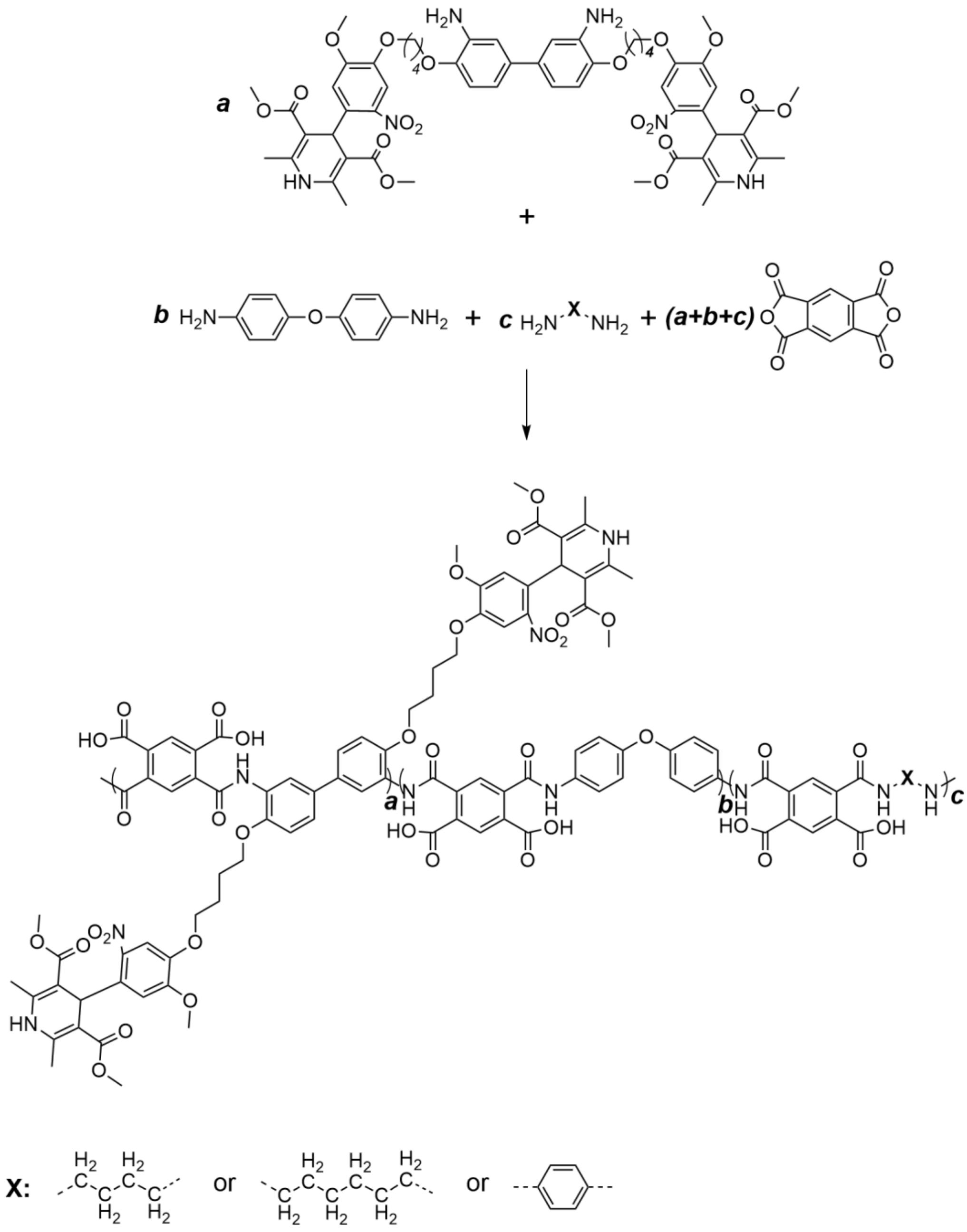

General procedure A: The photosensitive PAA polymers are synthesized by the polycondensation method. Briefly, all diamine monomers are dissolved in *N, N*-dimethylacetamide (DMAc) at 5 °C under dark. The solution is stirred for 30 minutes

at 5 ℃, and then the pyromellitic dianhydride (PMDA) is added into the solution in batches and stirred for 12 hours to afford photosensitive PAA solution. The 5 kinds of available diamine monomers that are listed in Supplementary Tab. 1 are employed to have a condensation polymerization with PMDA. They are photosensitive diamine monomer (PDM), 4,4-oxydianiline (ODA), 1,6-hexylenediamine (HMDA), 1,4-butanediamide (BDA), and *p*-phenylenediamide (PDA), respectively. Thus, a series of PAA films, including PDM-PMDA-ODA (PPO), PDM-PMDA-ODA-HMDA (PPOH), PDM-PMDA-ODA-BDA (PPOB), PDM-PMDA-ODA-PDA (PPOP), PDM-PMDA-PDA (PPP), and PDM-PMDA are synthesized.

PDM-PMDA-ODA (PPO): PDA (1.11 g, 1 mmol), PMDA (1.53 g, 7 mmol), ODA (1.20 g, 6 mmol), and 34.56 g DMAc are used to obtain PPO solution under general procedure A.

PDM-PMDA-ODA-HMDA-1 (PPOH-1): PDM (1.11 g, 1 mmol), PMDA (1.53 g, 7 mmol), ODA (1.06 g, 5.28 mmol), HMDA (0.08 g, 0.72 mmol), and 34.02 g DMAc are used to obtain PPOH-1 solution under general procedure A.

PDM-PMDA-ODA-HMDA-2 (PPOH-2): PDM (1.11 g, 1 mmol), PMDA (1.53 g, 7 mmol), ODA (0.90 g, 4.5 mmol), HMDA (0.17 g, 1.5 mmol), and 33.39 g DMAc are used to obtain PPOH-2 solution under general procedure A.

PDM-PMDA-ODA-HMDA-3 (PPOH-3): PDM (1.11 g, 1 mmol), PMDA (1.53 g, 7 mmol), ODA (0.72 g, 3.6 mmol), HMDA (0.28 g, 2.4 mmol), and 32.76 g DMAc are used to obtain PPOH-3 solution under general procedure A.

PDM-PMDA-ODA-HMDA-4 (PPOH-4): PDM (1.11 g, 1 mmol), PMDA (1.53 g, 7 mmol), ODA (0.54 g, 2.7 mmol), HMDA (0.38 g, 3.3 mmol), and 32.04 g DMAc are used to obtain PPOH-4 solution under general procedure A.

PDM-PMDA-ODA-HMDA-5 (PPOH-5): PDM (1.11 g, 1 mmol), PMDA (1.53 g, 7 mmol), ODA (0.36 g, 1.8 mmol), HMDA (0.49 g, 4.2 mmol), and 31.41 g DMAc are used to obtain PPOH-5 solution under general procedure A.

PDM-PMDA-ODA-BDA (PPOB): PDM (1.11 g, 1 mmol), PMDA (1.53 g, 7 mmol), ODA (0.72 g, 3.6 mmol), BDA (0.21 g, 2.4 mmol), and 32.13 g DMAc are used to obtain PPOB solution under general procedure A.

PDM-PMDA-ODA-PDA (PPOP): PDM (1.11 g, 1 mmol), PMDA (1.53 g, 7 mmol), ODA (0.72 g, 3.6 mmol), PDA (0.26 g, 2.4 mmol), and 32.58 g DMAc are used to obtain PPOP solution under general procedure A.

PDM-PMDA-PDA-1 (PPP-1): PDM (1.11 g, 1 mmol), PMDA (1.53 g, 7 mmol), PDA (0.65 g, 6 mmol), and 29.61 g DMAc are used to obtain PPP-1 solution under general procedure A.

PDM-PMDA-PDA-2 (PPP-2): PDM (1.11 g, 1 mmol), PMDA (1.74 g, 8 mmol), PDA (0.76 g, 7 mmol), and 32.49 g DMAc are used to obtain PPP-2 solution under general procedure A.

PDM-PMDA-PDA-3 (PPP-3): PDM (1.11 g, 1 mmol), PMDA (1.96 g, 9 mmol), PDA (0.86 g, 8 mmol), and 35.37 g DMAc are used to obtain PPP-3 solution under general procedure A.

PDM-PMDA-PDA-4 (PPP-4): PDM (1.11 g, 1 mmol), PMDA (2.18 g, 10 mmol), PDA (0.97 g, 9 mmol), and 38.34 g DMAc are used to obtain PPP-4 solution under general procedure A.

PDM-PMDA-PDA-5 (PPP-5): PDM (1.11 g, 1 mmol), PMDA (2.40 g, 11 mmol), PDA (1.08 g, 10 mmol), and 41.31 g DMAc are used to obtain PPP-5 solution under general procedure A.

PDM-PMDA: PDM (1.11 g, 1 mmol), PMDA (0.22 g, 1 mmol), and 11.97 g DMAc are used to obtain PDM-PMDA solution under general procedure A.

Supplementary Table 1. Monomers and the corresponding molar ratio of synthesizing 14 kinds of PAA polymers.

Samples	Molar ratio					
	PDM	PMDA	ODA	HMDA	BDA	PDA
PPO	1	7	6	0	0	0
PPOH-1	1	7	5.28	0.72	0	0
PPOH-2	1	7	4.5	1.5	0	0
PPOH-3	1	7	3.6	2.4	0	0
PPOH-4	1	7	2.7	3.3	0	0
PPOH-5	1	7	1.8	4.2	0	0
PPOB	1	7	3.6	0	2.4	0
PPOP	1	7	3.6	0	0	2.4
PPP-1	1	7	0	0	0	6
PPP-2	1	8	0	0	0	7
PPP-3	1	9	0	0	0	8
PPP-4	1	10	0	0	0	9
PPP-5	1	11	0	0	0	10
PDM-PMDA	1	1	0	0	0	0

Section 2: Structural properties of photosensitive diamine monomer

Section 2.1 Photosensitive properties analysis

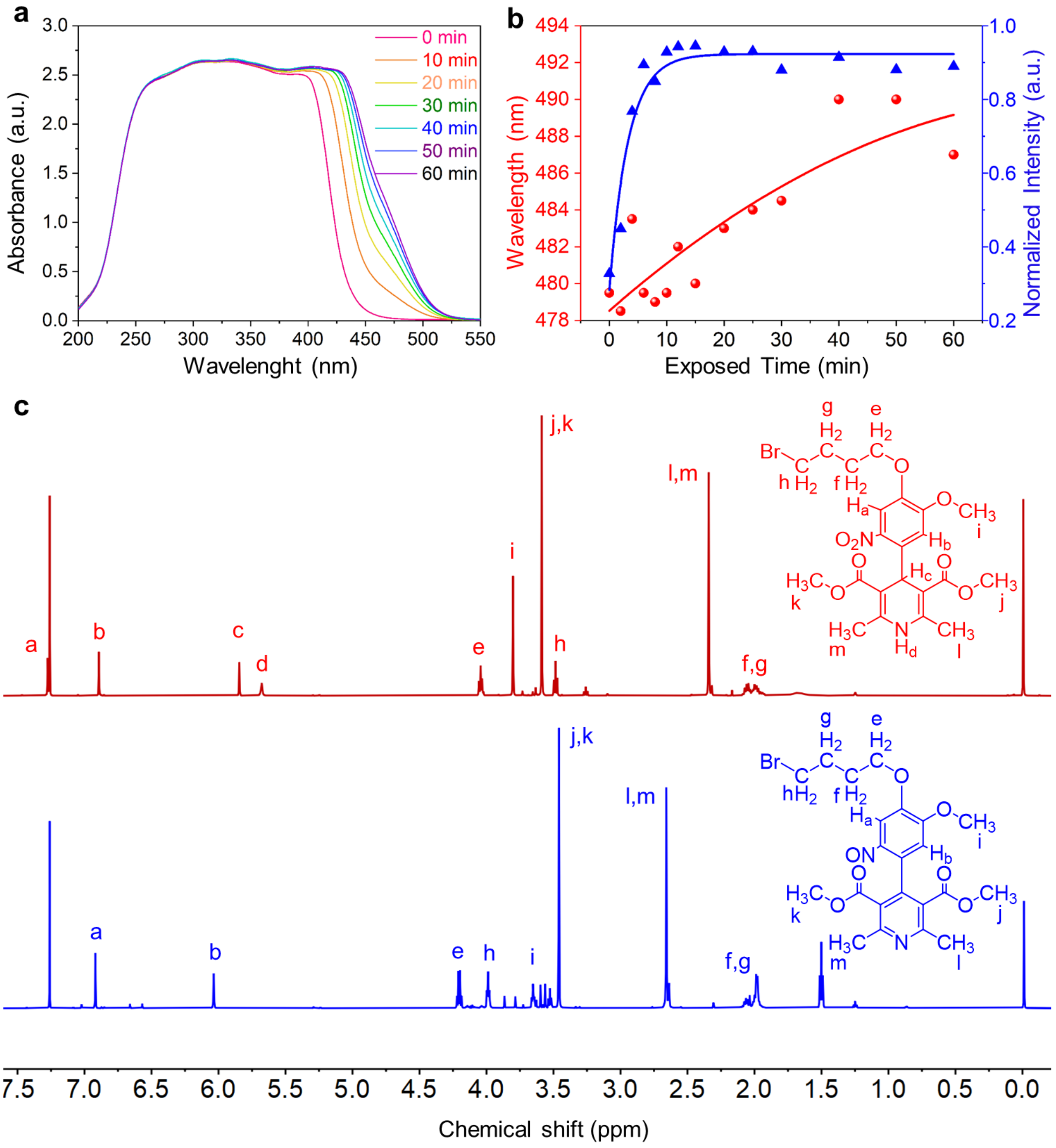

Supplementary Figure 2. Photosensitive properties of Compound ii. (a) UV-vis absorption spectrum of the ethanol solutions of Compound ii (1 mmol L^{-1}) that is irradiated by 405 nm light under different exposed times. (b) The locations and corresponding emission peaks intensities of the Compound ii ethanol solution (1 mmol L^{-1}) in 430-nm wavelength excitation under different exposed times after

irradiated by 405-nm light. (c) ^{1}H NMR spectra ($CDCl_3$, 600 MHz) of Compound ii before (red) and after (blue) irradiation by 405-nm light.

Section 2.2 Thermal stability analysis

The thermal stability of Compounds i, ii and v is also performed by thermogravimetric analyses (TGA) under nitrogen and air atmosphere, respectively. Even under the air atmosphere, all three compounds showed thermal stability well relatively below 212 ℃ (the thermal stability of compounds is i > ii > v). The decomposition temperatures for a 5% weight loss of these three compounds are listed in Supplementary Tab. 2. Compared with Compound i, Compound ii and v show lower thermal stability caused by alkyl chains and amino groups.

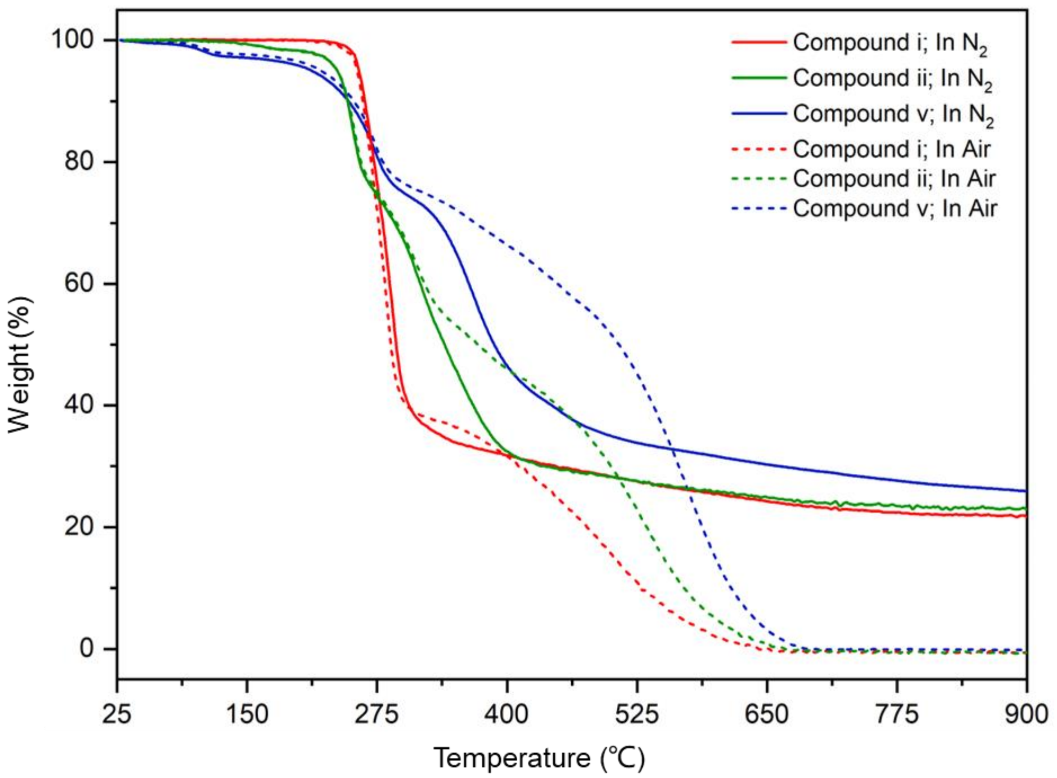

Supplementary Figure 3. The TGA thermograph of Compounds i, ii, and v.

Supplementary Table 2. The decomposition temperatures (T_{dec}) for a 5% weight loss of Compounds i, ii, and v.

Compounds	T_{dec} (℃) in N_2	T_{dec} (℃) in air
i	257.2	255.2
ii	236.8	236.8
v	212.0	221.0

Section 3: Structural properties of photosensitive polyamic acid

Section 3.1 Nuclear magnetic resonance spectroscopic analysis

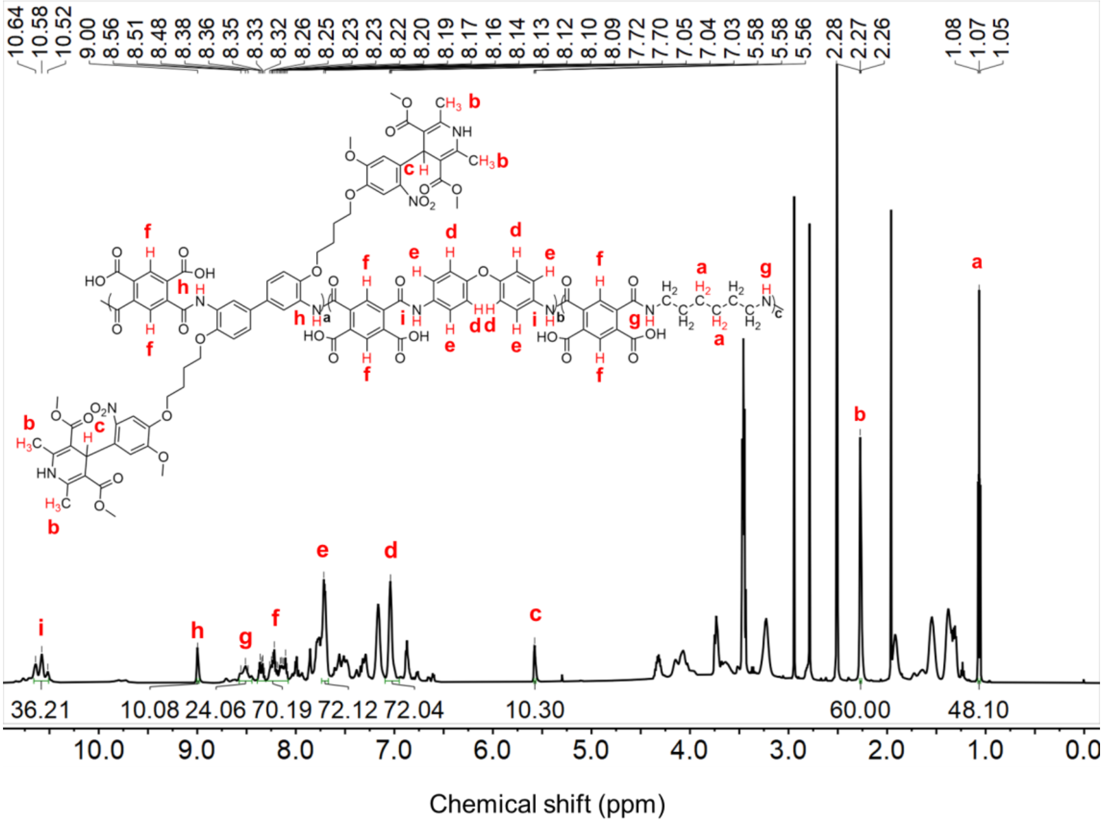

Supplementary Figure 4. ^{1}H NMR spectra of PAA (PPOH-3).

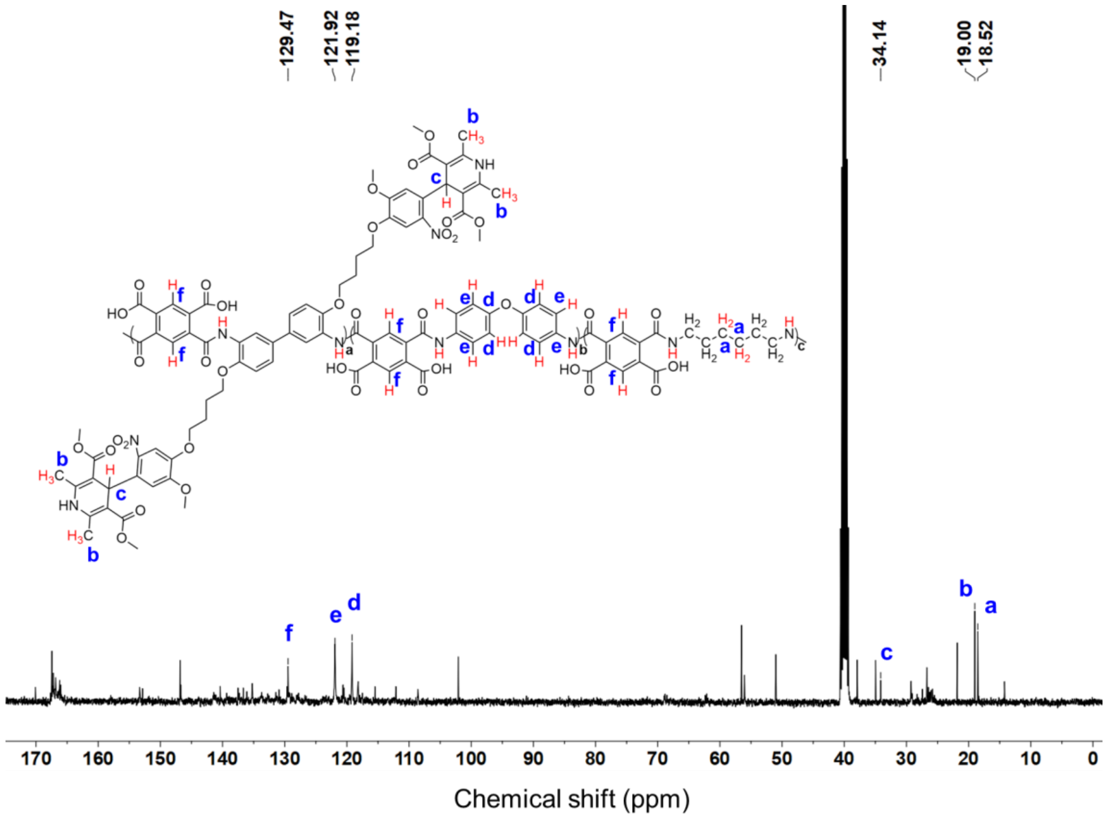

Supplementary Figure 5. ^{13}C NMR spectra of PAA (PPOH-3).

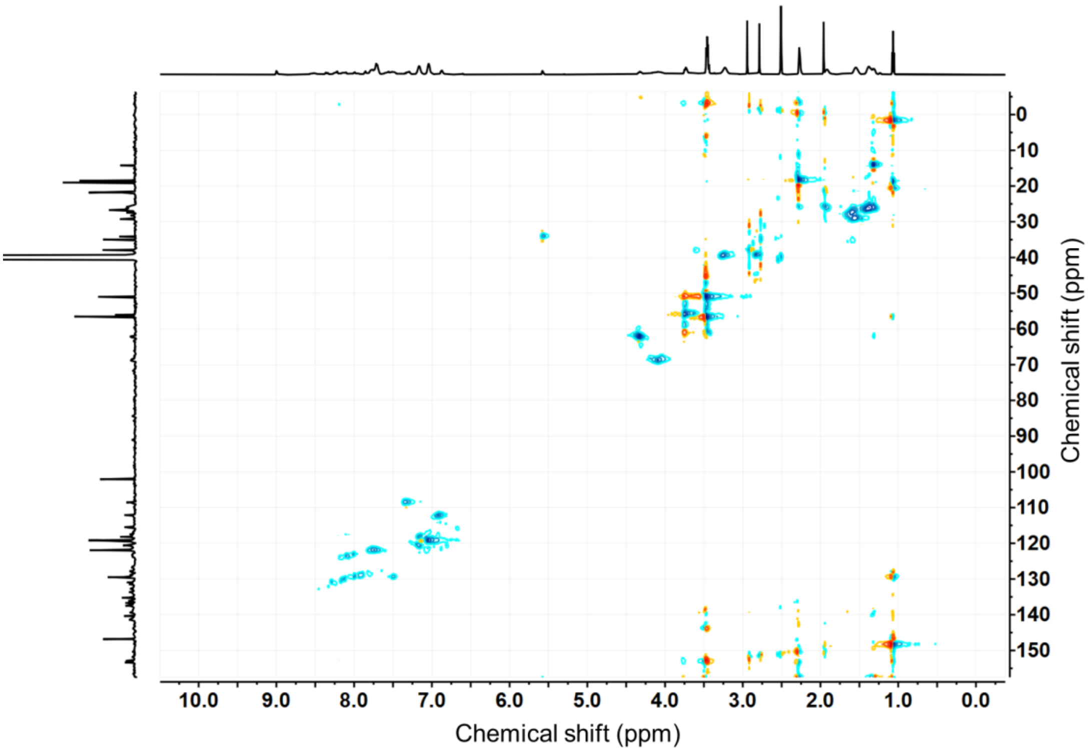

Supplementary Figure 6. Two-dimension (2D) NMR spectra of PAA (PPOH-3).

Section 3.2 Fourier transform infrared spectroscopic analysis

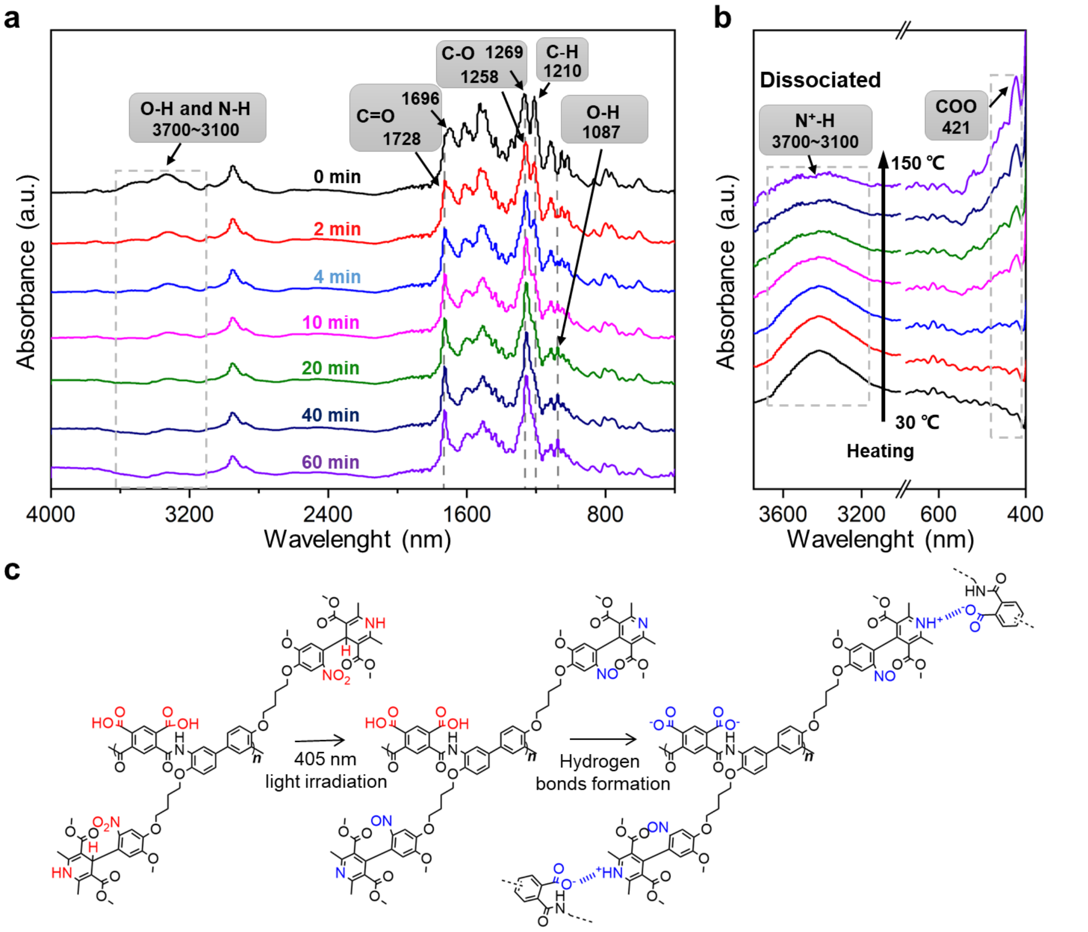

Supplementary Figure 7. Ultraviolet exposure induces the formation of intermolecular hydrogen bonds in photosensitive PDM-PMDA films[3]. (a) The normalized FTIR spectra of the PDM-PMDA film under different 405-nm light irradiation durations. The changes of five characteristic absorption bands of the film with 405-nm light irradiation[4,5,6]: the C-H bending vibrations peak at 1210 cm^{-1} is disappeared as 1,4-dihydropyridine structures decomposing into 2,6-dimethylpyridinium structures completely, it appears a new absorption peak at 1087 cm^{-1} which is attributed to the out-of-plane torsional vibrations of O-H in intermolecular hydrogen bond of N^{+}-H···O^{-} between 2,6-dimethylpyridinium structure and hydrogen benzoate ion, a decrease of the vibration intensity of O-H and

N-H around 3700-3100 cm^{-1} is attributed to the ionization of the hydrogen benzoate induced by the 2,6-dimethylpyridinium structures, the hydrogen bond of N^{+}-H···O^{-} lengthens the C-O bound within carboxyl structure, which causing red shift from 1269 cm^{-1} to 1258 cm^{-1} of the C-O stretching vibration, the competitive N atom of 2,6-dimethylpyridinium structure decreases the capability of forming hydrogen bond of C=O that results blue shift from 1696 cm^{-1} to 1728 cm^{-1} of the C=O stretching vibration. (b) Temperature-dependent FTIR spectra of the irradiated PDM-PMDA film (exposed time: 60 min). As all 1,4-dihydropyridine structures decompose into 2,6-dimethylpyridinium structures completely, the absorption peaks in 3700-3100 cm^{-1} are attributed to the vibration of N^{+}-H. With temperature increasing, the N^{+}-H vibration intensity decreases gradually with it dissociated. Meanwhile, the in-plane rocking vibration intensity at 421 cm^{-1} of COO^{-} increases with the dissociation of N^{+}-H···O^{-}. (c) N^{+}-H···O^{-} formed in PDM-PMDA during irradiation.

Section 3.3 Young's modulus determined by AFM

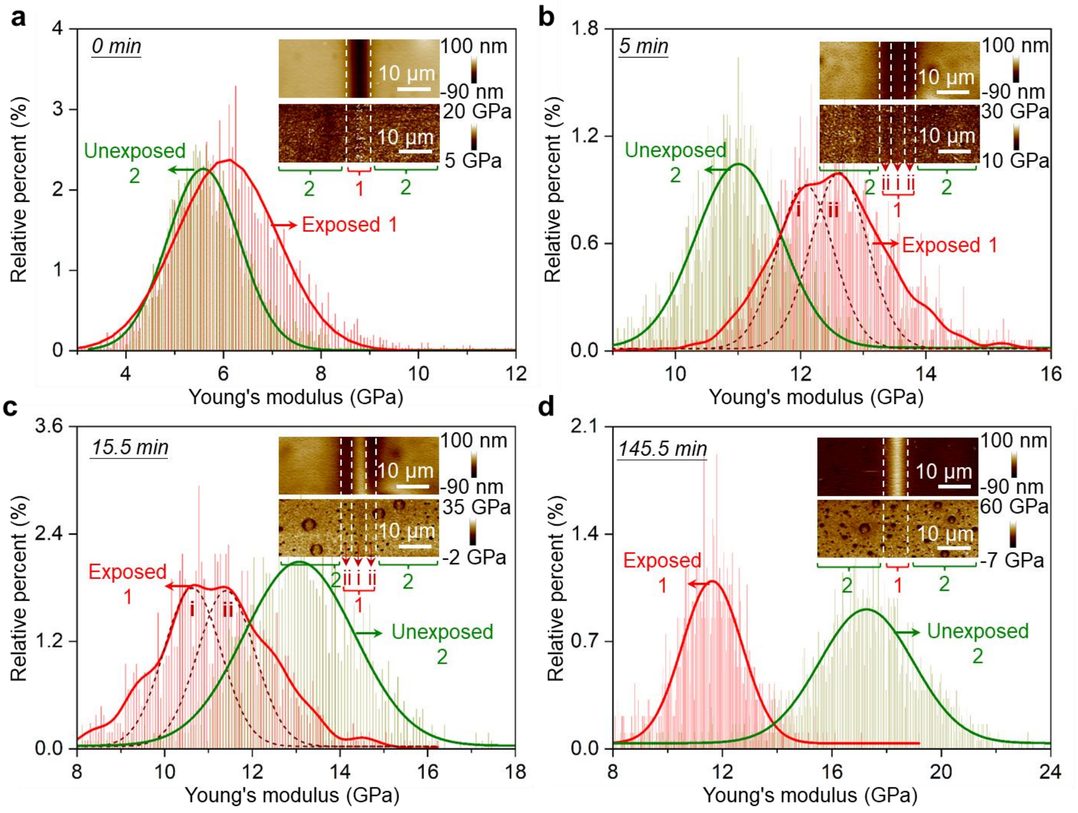

Supplementary Figure 8. The evolution of morphology and Young's modulus of the exposed and unexposed areas of the PPOH-3 film under an atmosphere of ethanol vapor at different times revealed by in-situ AFM. (a-d), Normalized Young's modulus profile of both exposed (red) and unexposed (green) areas. The inserts show the corresponding morphology images and Young's modulus maps. (a) At 0 minute. (b) At 5 minutes. The suction of the residual solvent (*N*, *N*-dimethylacetamide) by ethanol vapor gradually increases the Young's modulus of both exposed area and unexposed area. The exposed area is divided into two parts (i, ii) with different Young's modulus due to gradual diffusion of the residual solvent. (c) At 15.5 minutes. (d) At 145.5 minutes. The suction of the residual solvent by ethanol vapor reaches equilibrium.

The exposed area has more residual solvent than that of unexposed area, and therefore has a lower Young's modulus value.

Section 3.4 Residual solvent absorption and desorption analysis

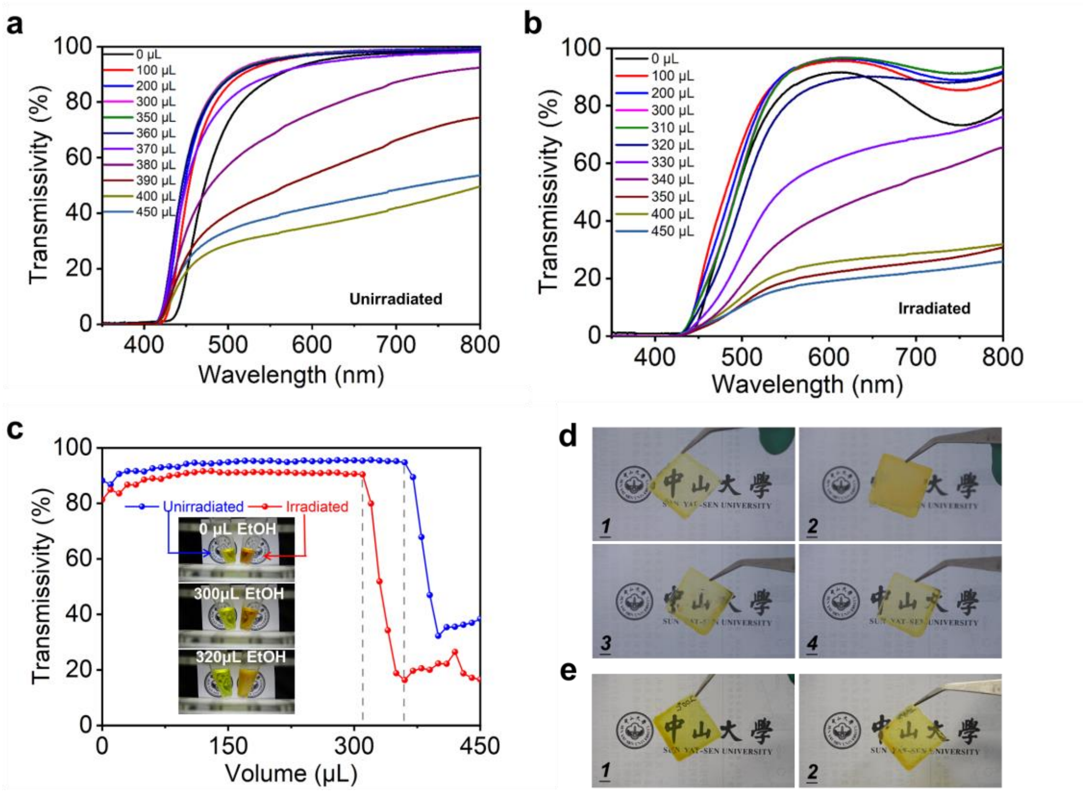

Supplementary Figure 9. The solvent flux of the PAA film surface is described qualitatively by the gelation rate of the polymer in ethanol. (a) The transmissivity of unirradiated PAA (PPOH-3) solution (100 μL) with solid content of 10% under different volume of added ethanol. The transmissivity of the solution decreased when the additional ethanol volume reached 370 μL. (b) The transmissivity of PAA (PPOH-3) solution (100 μL) with solid content of 10% irradiated by 405-nm light under different volume of added ethanol. The transmissivity of the solution decreased when the added ethanol volume reached 320 μL. (c) The transmissivity of two kinds *N*, *N*-dimethylacetamide solutions of PAA (PPOH-3) under a fixed wavelength (550 nm) under different the volume of added ethanol. Insets, the apparent turbidity of the two kinds solutions before (left centrifuge tube) and after (right centrifuge tube)

irradiation under different volume of added ethanol. (d) After prebaking at 60 ℃ for 2 hours, the transparency changes of the PAA (PPOH-3) film under different conditions. Step 1. Original state. Step 2. After being immersed in ethanol for 10 seconds. Step 3. The sample is blown by hot wind for 5 seconds after step 2. Step 4. The sample is blown by hot wind for 5 seconds after step 3. (e) After prebaking at 200 ℃ for 2 hours, the transparency changes of the PAA (PPOH-3) film under different conditions. Step 1. Original state. Step 2. After being immersed in ethanol for 10 seconds.

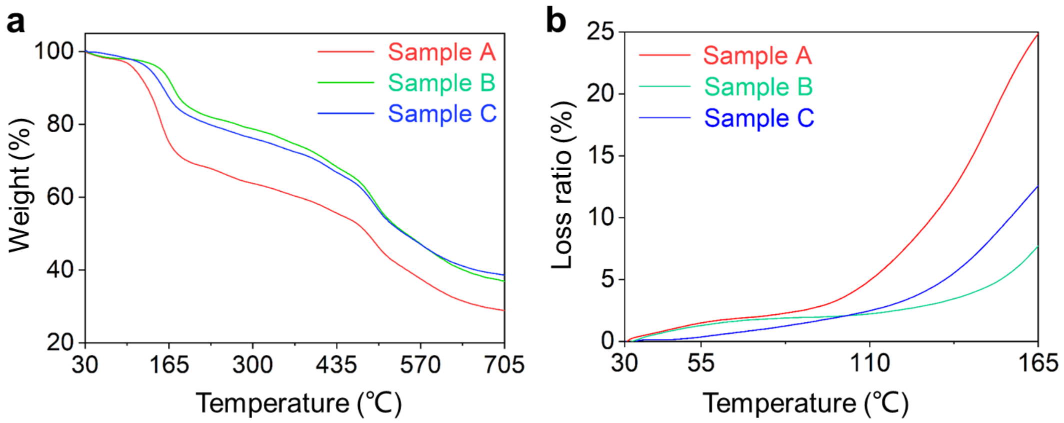

Supplementary Figure 10. Thermogravimetric curves. The residual quantity of solvent (DMAc) in PPOH-3 film samples is influenced by irradiation and ethanol based on (a) thermogravimetric loss and (b) thermogravimetric loss ratio. Sample A, Original PPOH-3 film. Sample B, PPOH-3 film which immersed in ethanol directly for 10 seconds. Sample C, PPOH-3 film which irradiated by 405-nm light and then immersed in ethanol for 10 seconds. As temperature increasing, those samples showed different thermal stability (relative thermal stability is sample B > C > A) at 165 ℃ which is the boiling point of the residual solvent (DMAc). Both the residual solvent (DMAc) in sample B and sample C is sucked out by ethanol, resulting in lower thermogravimetric loss ratio than the original sample[7]. The hydrogen bond crosslinking networks formed on the surface of sample C because 405-nm light irradiation could obstruct the diffusion of residual solvent (DMAc), resulting in higher thermogravimetric loss ratio than sample B.

Supplementary Table 3. The thermogravimetric loss of the PPOH-3 film with different treatments.

Treatment method	T_{dec} (°C) for 1% weight loss	T_{dec} (°C) for 5% weight loss	T_{dec} (°C) for 25% weight loss
Original film (without any treatment)	45	110	165
Immersed in ethanol for 10 seconds, then blow-dried by N_2	35	147	354
Irradiated by UV light for 60 seconds, then immersed in ethanol for 10 seconds and blow-dried by N_2	75	135	280

Section 4: The formation of wrinkles on photosensitive polyamic acid films

Section 4.1 The fabrication of uniform wrinkles in a large area

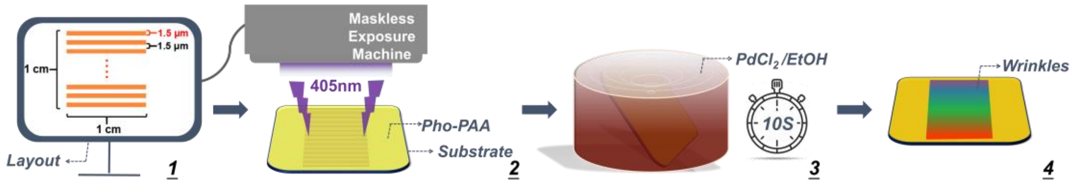

Supplementary Figure 11. The preparation process schematic of grating wrinkles on PAA films.

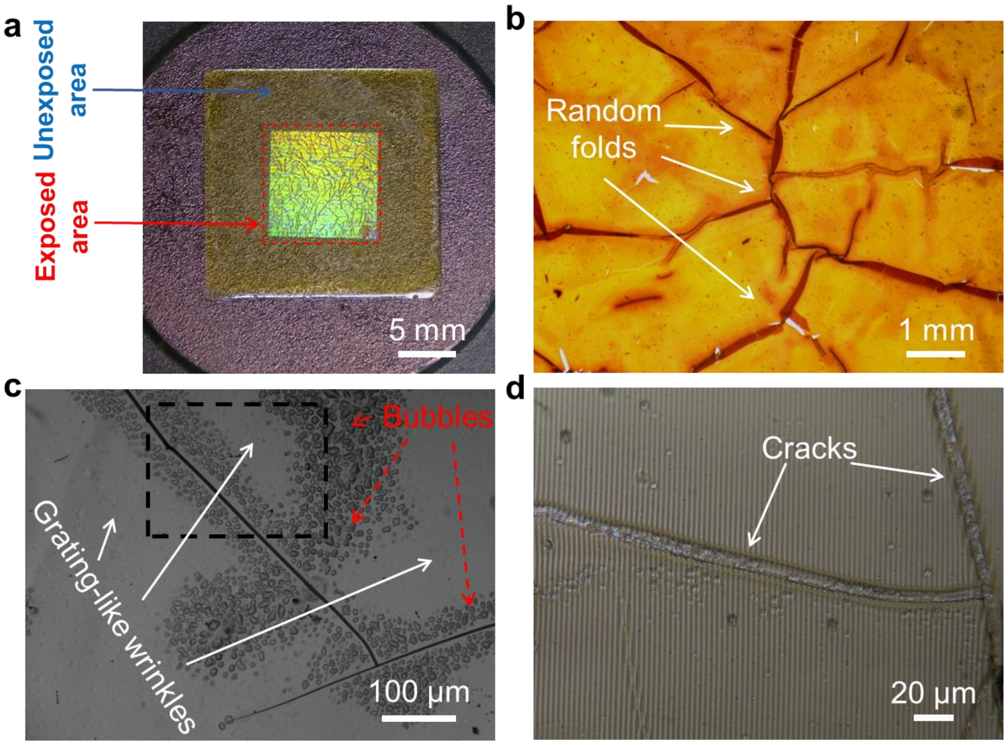

Supplementary Figure 12. Defective wrinkles with a line width of 1.5 μm and spacing of 1.5 μm formed on PPOH-3 film in pure ethanol. (a) Photograph of PPOH-3 sample after developing in ethanol. (b) Obvious random wrinkles on the film surface. (c-d) Optical microscope images of the undesigned bubbles (c) and creases in irradiated area (d).

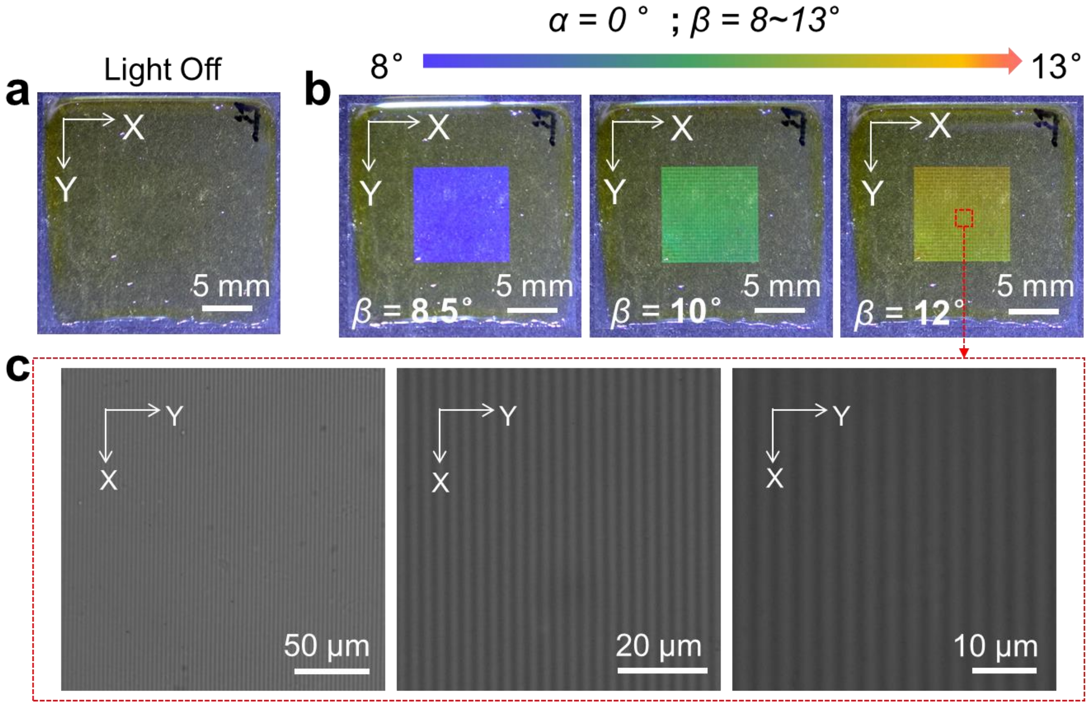

Supplementary Figure 13. The structural color on the film is due to the designed wrinkles under white light. (a) Photograph of irradiated PPOH-3 sample after developing in $PdCl_2$ / ethanol solution. (b) Angle-dependent structural colors on PPOH-3 film under vertical white light irradiation (α = 0°). (c) Optical microscope images of designed wrinkling structures with a line width of 1.5 μm and spacing of 1.5 μm on PPOH-3 film.

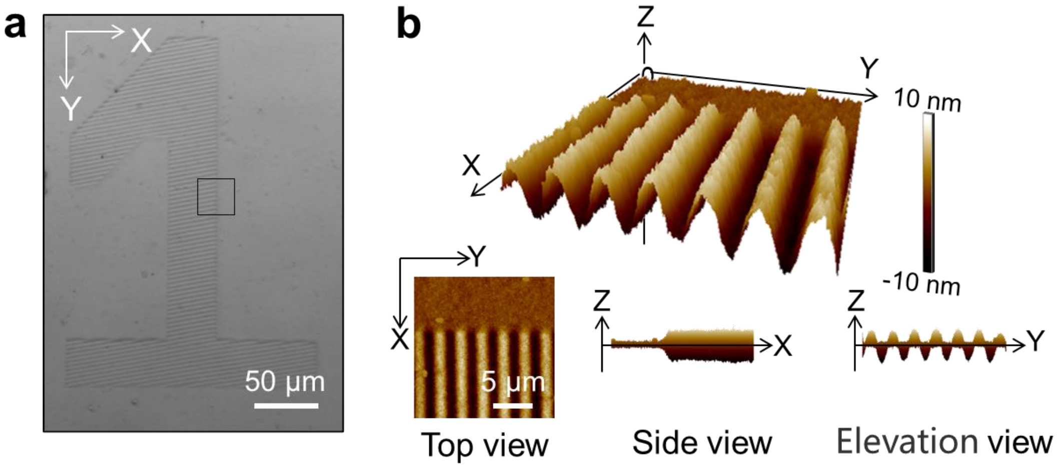

Supplementary Figure 14. The edge morphology of wrinkles. (a) An image of the number "1" constructed by wrinkles on the polyimide (PI, thermal imidized PPOH-3) film. (b) Three-dimension (3D) AFM images of the wrinkles' boundary on PI film. The wrinkles with relief structure on the film show sinusoidal morphology relative to the plane's unexposed area.

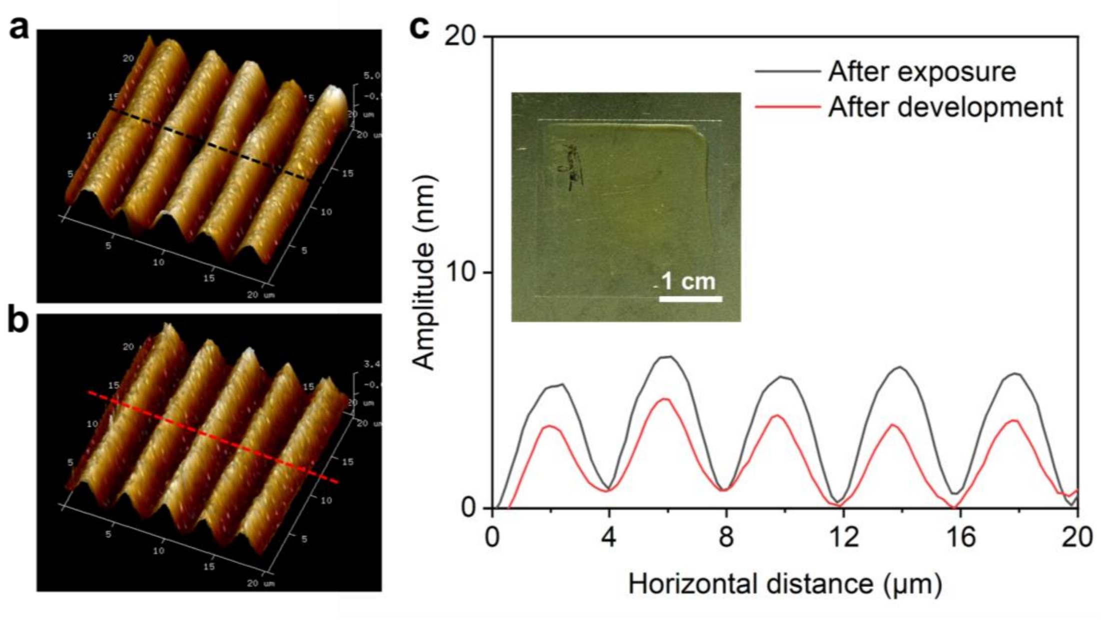

Supplementary Figure 15. Wrinkling structures on PAA film (PMDA: ODA = 1: 1) mixed with PDM. AFM images of the film after (a) exposure and then followed by (b) development. (c) Optical image and surface morphology of the film.

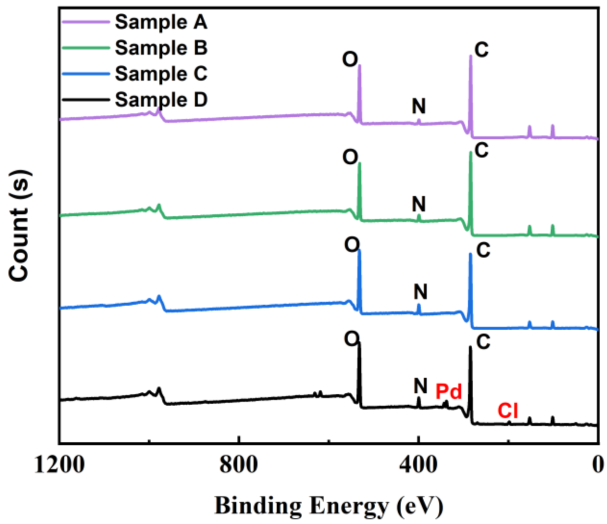

Supplementary Figure 16. The XPS spectra of PPOH-3 films before and after immersion in ethanol with $PdCl_2$. Sample A: Original PAA film; Sample B: Only irradiated by 405-nm light; Sample C: Developed by $PdCl_2$ / ethanol solution without irradiation; Sample D: Developed by $PdCl_2$ / ethanol solution after irradiation.

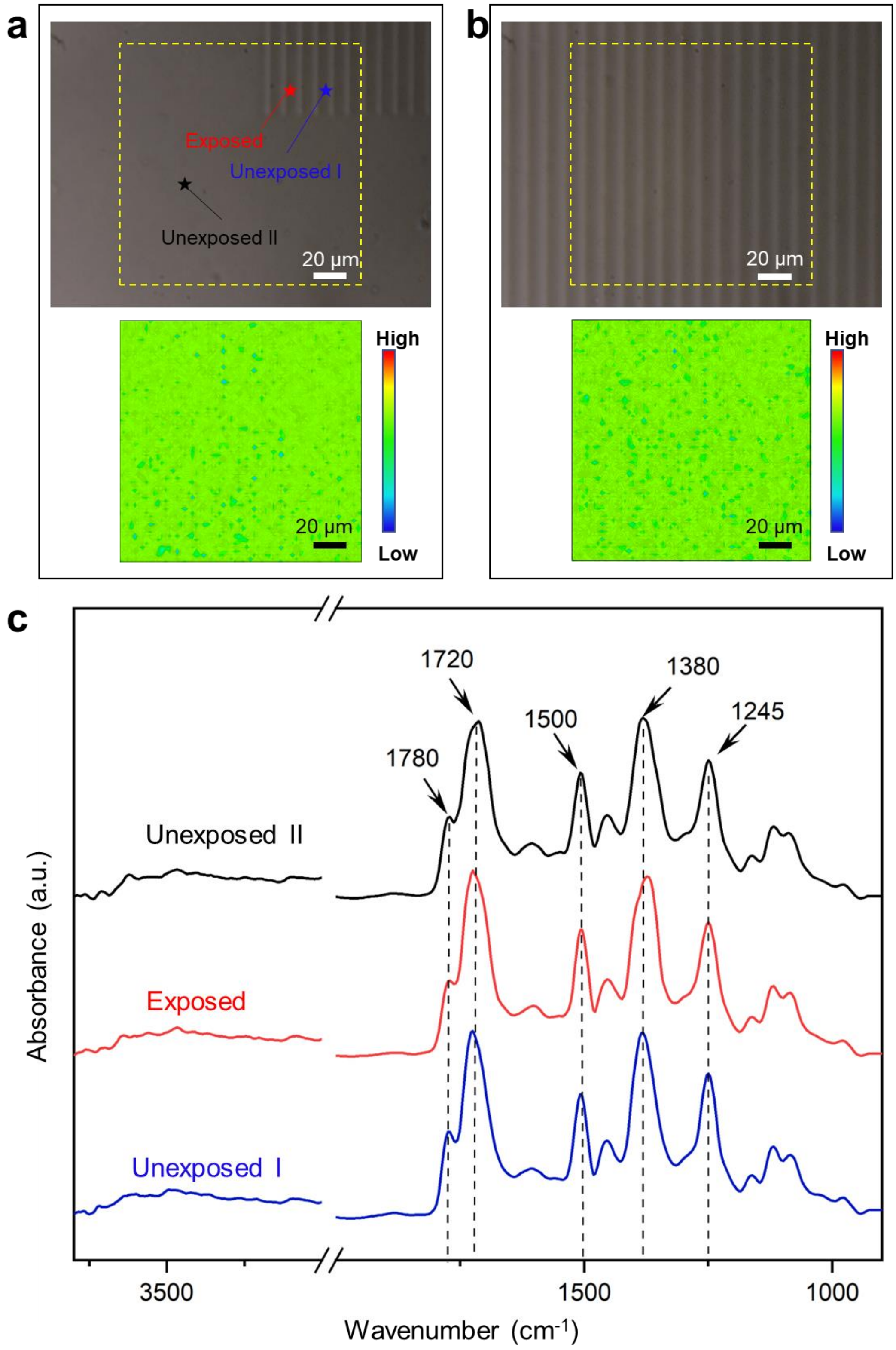

Supplementary Figure 17. Characterization of the structure homogeneity of the wrinkles on the imidized PPOH-3 film. Optical microscope image and corresponding micro-infrared spectroscopy analysis of (a) the border of the wrinkles and (b) the

wrinkles on imidized PPOH-3 film. The scanned micro-infrared spectroscopy map is generated by chemical mapping of selected areas in scanned frequency range of 873-3856 cm^{-1}. (c) The comparison of the normalized FTIR spectra of three different positions on imidized PPOH-3 film. The characteristic absorption bands at 1780 and 1720 cm^{-1} are caused by the asymmetric and symmetrical stretching vibrations of carbonyl groups in the imide rings, at 1500 cm^{-1} is classified as the vibration of benzene rings, at 1380 cm^{-1} is attributed to the stretching vibration of the C-N bond in the imide rings, and at 1245 cm^{-1} is classified as the stretching vibrations of C-O-C in the aromatic ether structures.

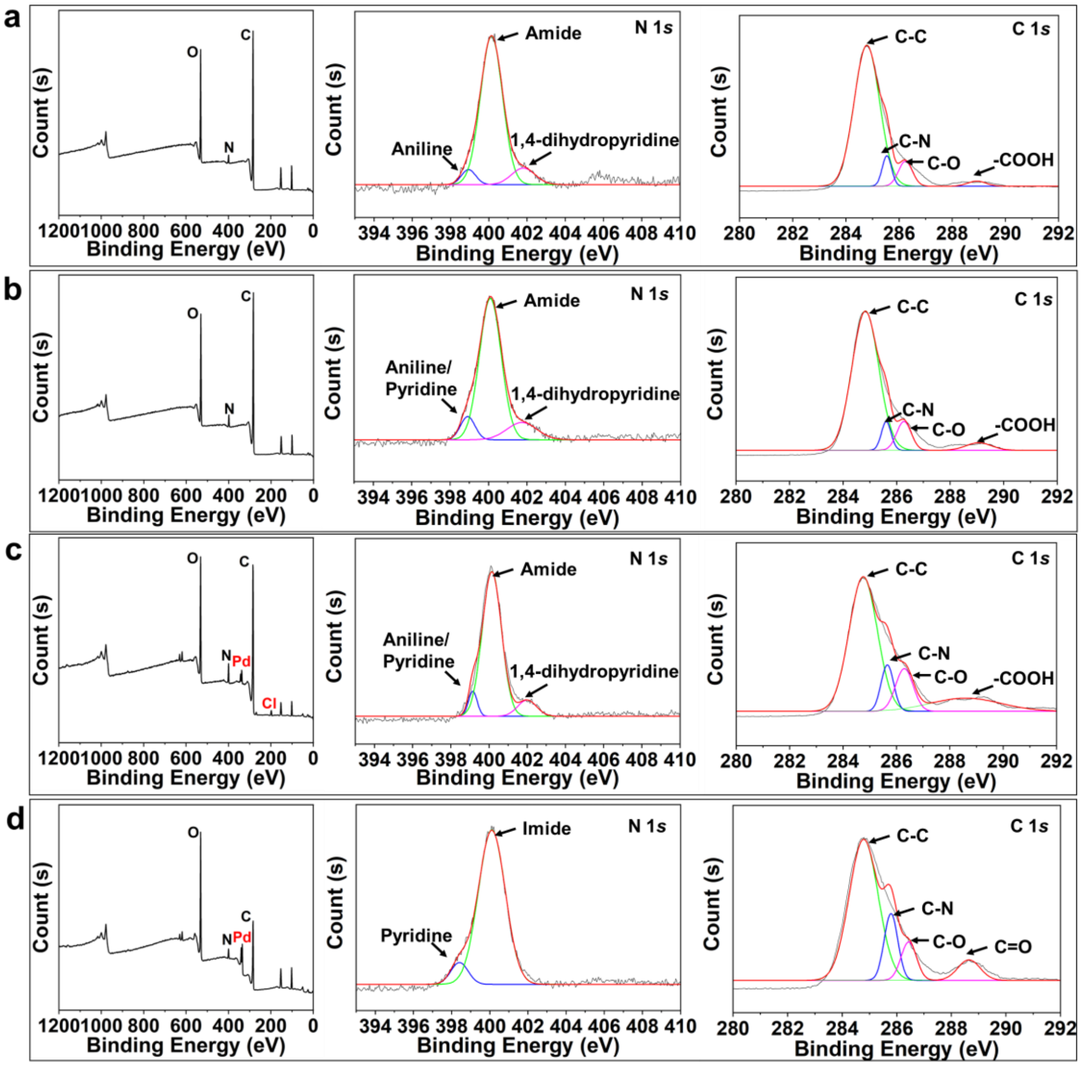

Supplementary Figure 18. Full XPS spectra, N 1*s* and C 1*s* XPS spectra of PPHO-3 film (a) before irradiation, (b) after irradiation, (c) after immersion in ethanol with $PdCl_2$, and (d) after thermal treatment at 300 °C, respectively.

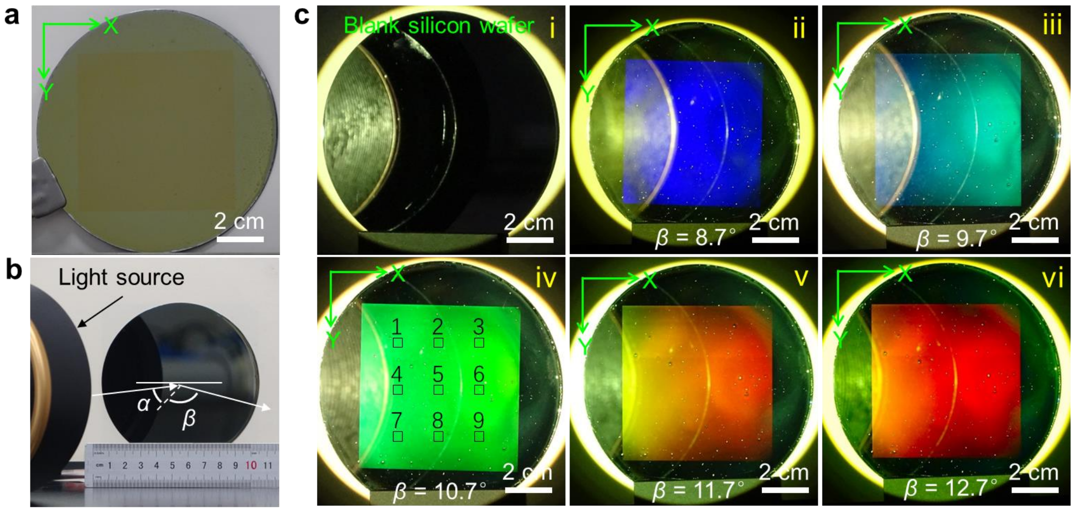

Supplementary Figure 19. Homogenous wrinkles over wafer scale. (a) Wrinkled structures with both line width and spacing of 1.5 μm formed on PPOH-3 film on a four-inch silicon wafer. (b) Schematic illustration of angle (α) between irradiation light and surface normal as well as (β) between viewing light and surface normal. (c) Optical images of bare silicon wafer, and wrinkled PPOH-3 film on wafer under different viewing angles (β).

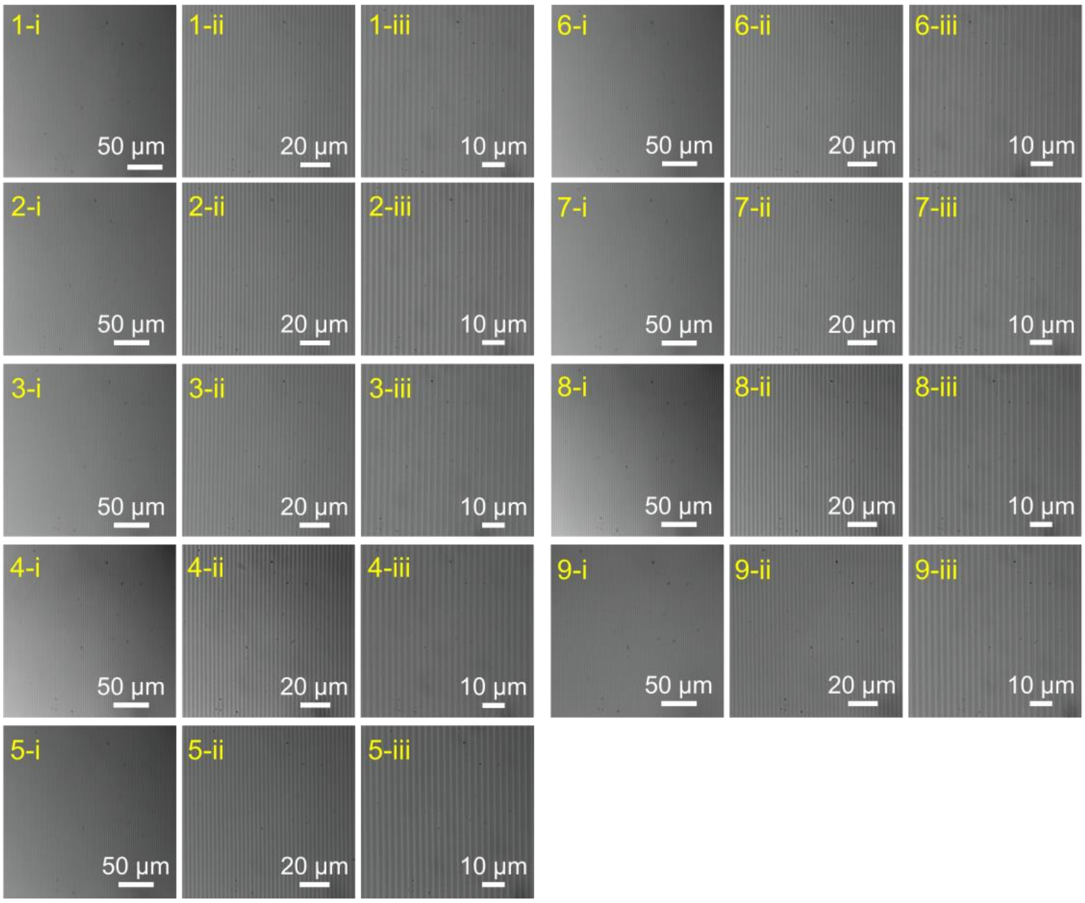

Supplementary Figure 20. The optical microscope images (i, ii, iii for different magnification) in 9 different locations (see in Supplementary Figure 19c) on the wrinkled PPOH-3 film.

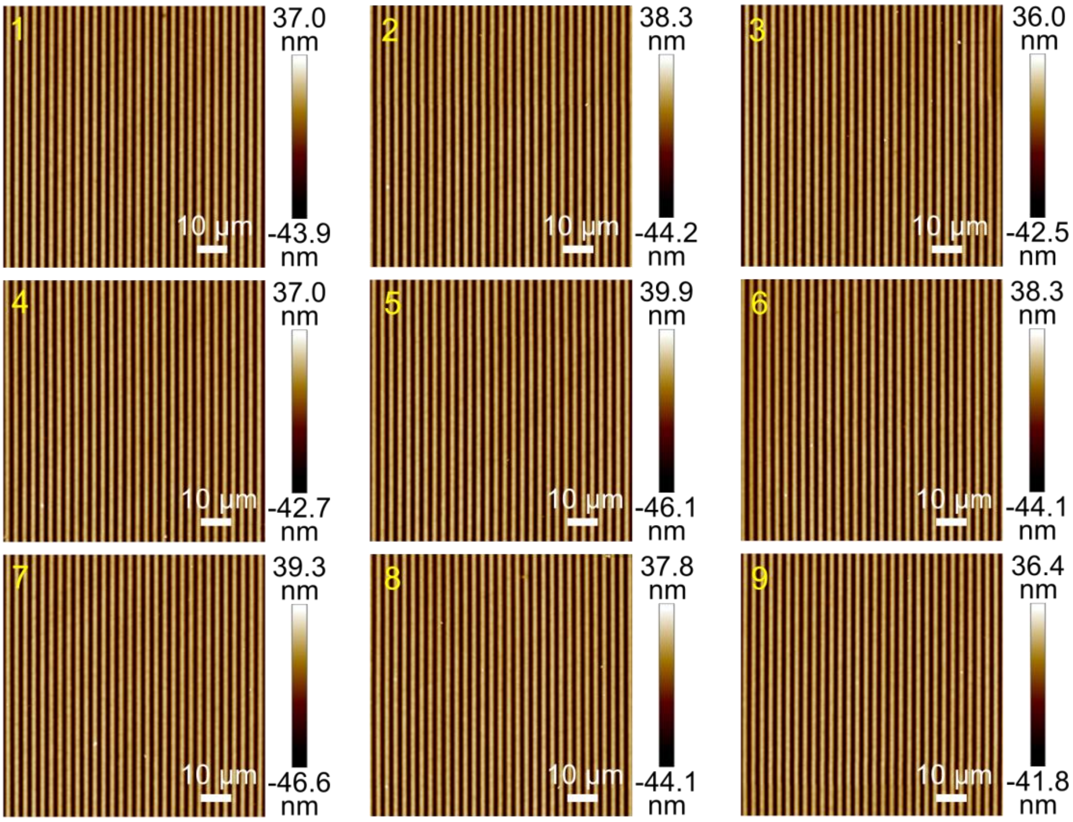

Supplementary Figure 21. AFM images in 9 different locations (see in Supplementary Figure 19c) on the wrinkled PPOH-3 film.

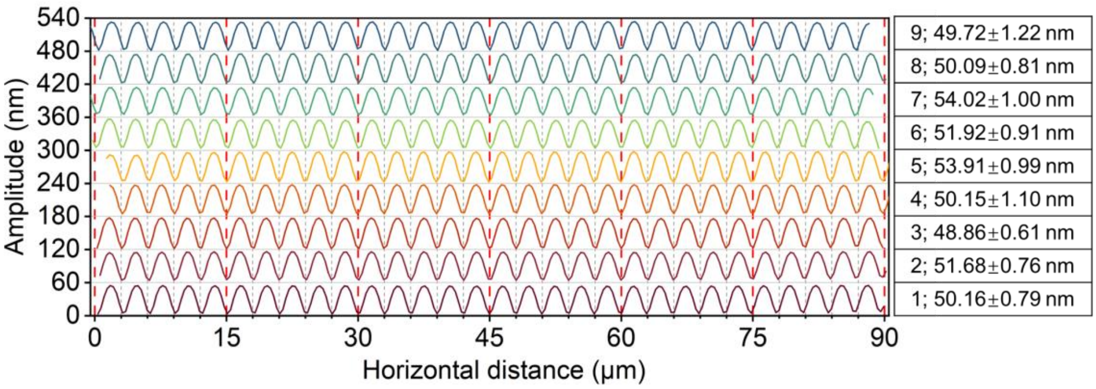

Supplementary Figure 22. The comparison of the profiles and the corresponding amplitude of wrinkled PPOH-3 film in 9 different locations (see in Supplementary Figure 19c).

Section 4.2 Controllable adjustment of wrinkles morphology

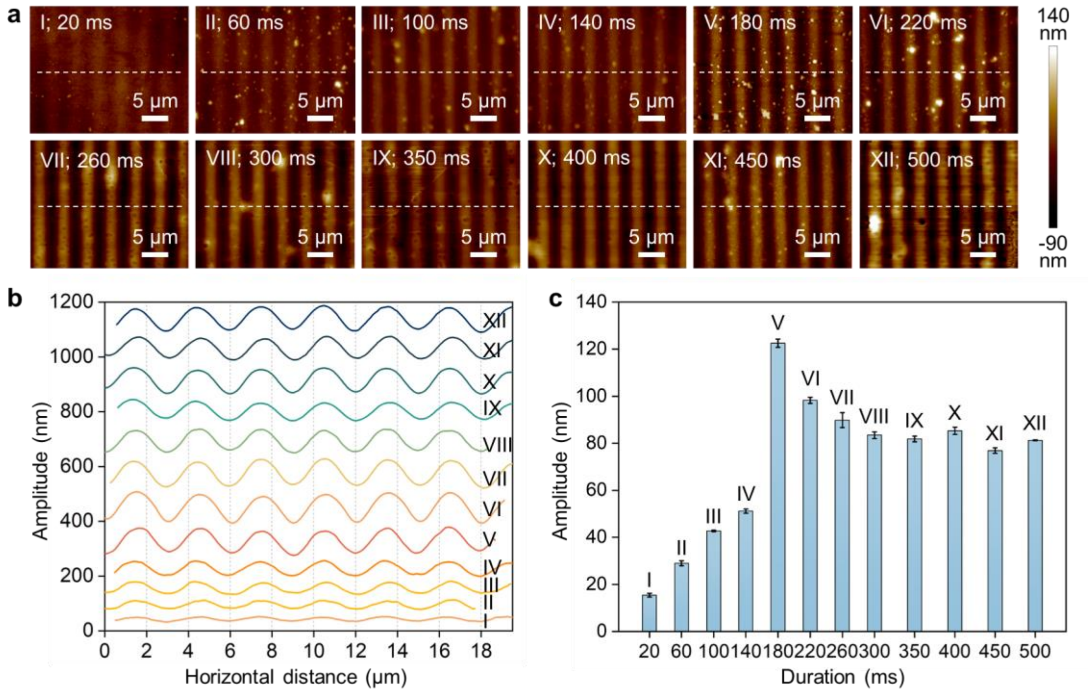

Supplementary Figure 23. Effects of irradiation time on the amplitude of wrinkles with 405 nm light. (a) AFM topological images of the wrinkles with both line width and spacing of 1.5 μm on PPOH-3 films under different irradiation time after development in ethanol containing $PdCl_2$. (b) AFM height profile images of 12 kinds of wrinkling structures under different irradiation time. (c) Comparison of the average amplitude of 12 kinds of wrinkling structures under different irradiation time. The maximum amplitude of the samples is 122 nm with an irradiation time of 180 ms. The error bars span a range of two standard deviations.

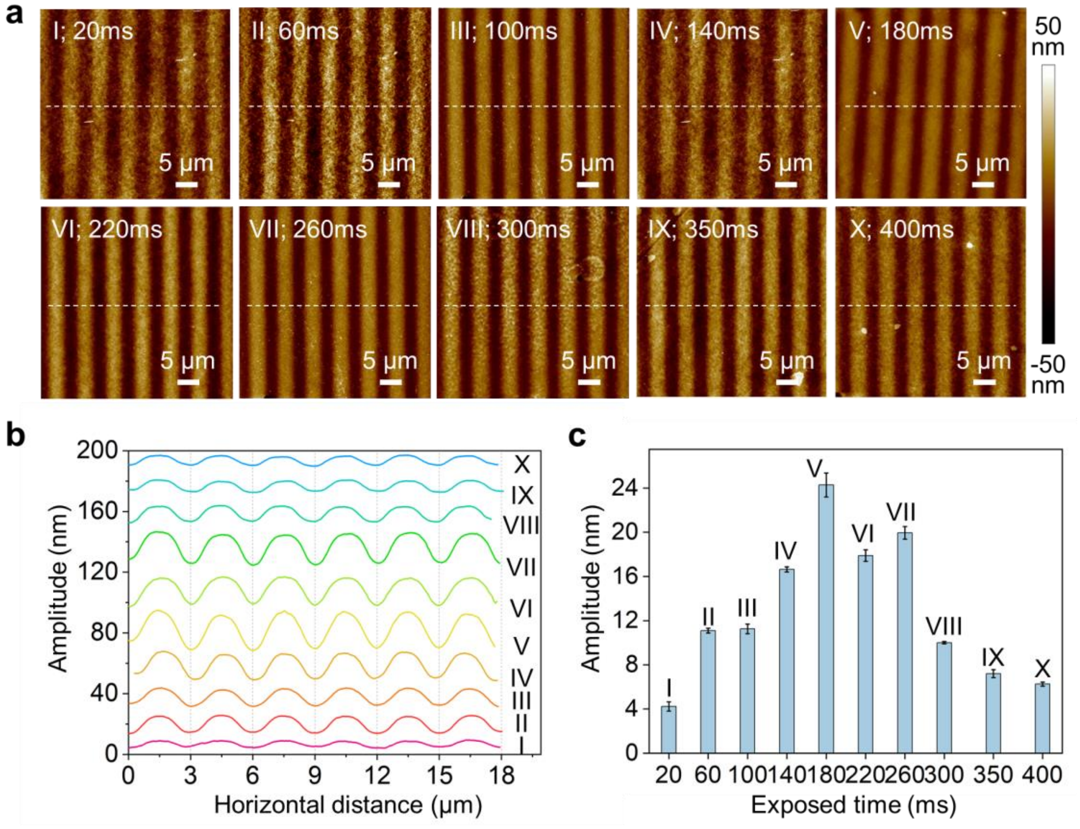

Supplementary Figure 24. Effects of 405-nm light exposed time on the amplitude of wrinkles. (a) The wrinkles AFM images of PPO (PDM: PMDA: ODA = 1: 7: 6) films. The wrinkles formed after developing in ethanol containing $PdCl_2$ have a same line width (1.5 μm) and spacing (1.5 μm) but different amplitudes under different exposed times. (b) AFM profile images of 10 kinds of wrinkling structures under different exposed times. (c) Average amplitude of 10 kinds of wrinkles. The maximum amplitude of samples is 24 nm with exposed time of 180 ms. The error bars span a range of two standard deviations.

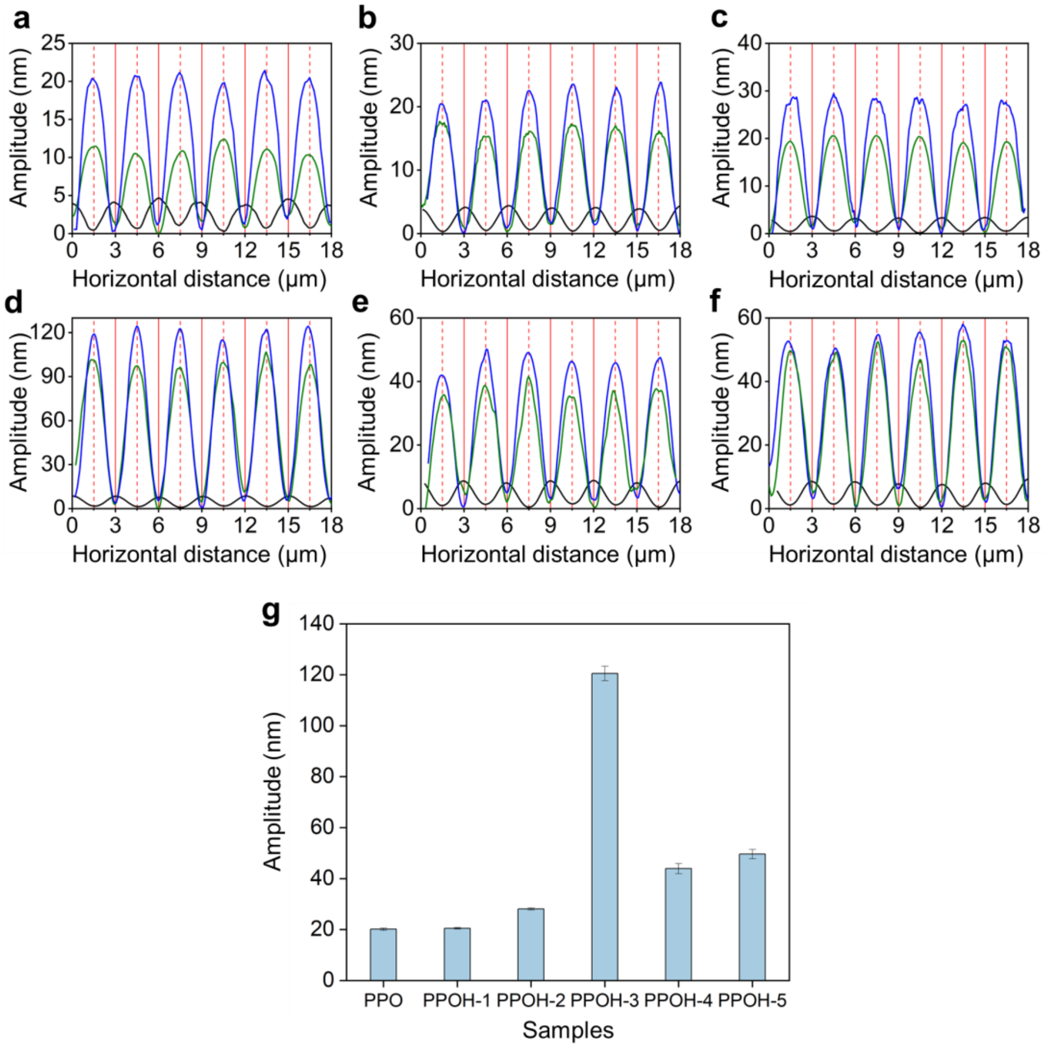

Supplementary Figure 25. The flexibility of PAA molecular chains affects the amplitude of wrinkles. (a-f) Profiles of PPO film and 5 kinds of PPOH film at different stages obtained from AFM. Stage 1. Only irradiated by 405-nm light with an exposed time of 180 ms but without developing (black lines). Stage 2. Developed by ethanol after irradiation (green lines). Stage 3. Developed by $PdCl_2$ / ethanol solution after irradiation (blue lines). a, PPO; b, PPOH-1; c, PPOH-2; d, PPOH-3; e, PPOH-4; f, PPOH-5. (g) Comparing the average amplitude of 6 kinds of those films after $PdCl_2$ / ethanol solution developing. Among them, the amplitude of the PPOH-3 film has the maximum value (122 nm). The error bars span a range of two standard deviations.

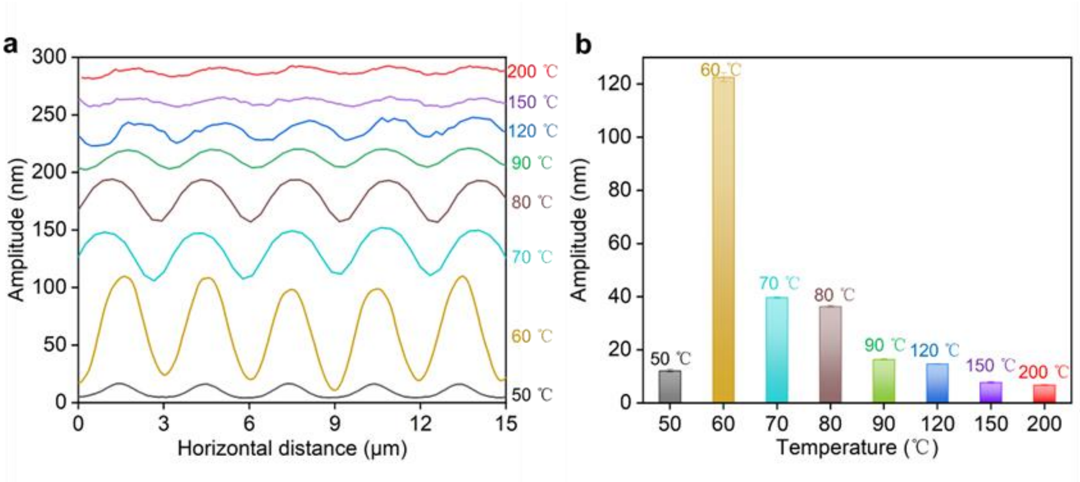

Supplementary Figure 26. The effects of the baking temperatures on the morphology of the wrinkled patterns on PPOH-3 films. (a) AFM height profile images of wrinkling structures under different baking temperature (50 ℃, 60 ℃, 70 ℃, 80 ℃, 90 ℃, 120 ℃, 150 ℃, 200 ℃). (b) Comparison of the average amplitude the wrinkling structures under different baking temperature. The wrinkles on the film baked after 60 ℃ have the maximum amplitude. The error bars span a range of two standard deviations.

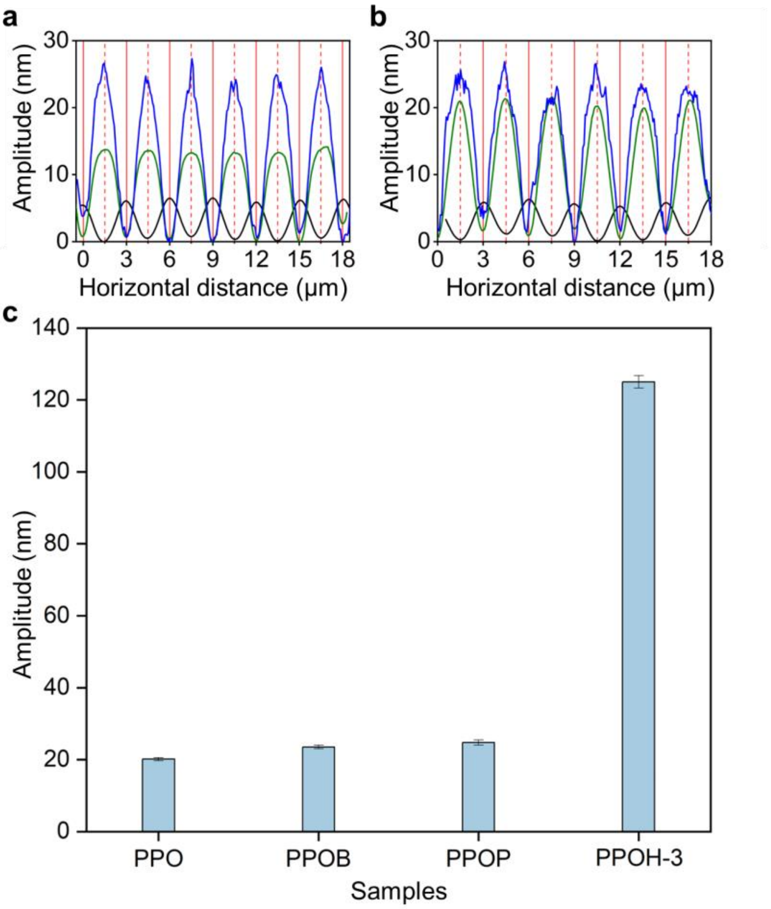

Supplementary Figure 27. The rigid segment structure in PAA molecular chains affects the amplitude of wrinkles. Profiles of (a) PPOB film and (b) PPOP film at different stages obtained from AFM. Stage 1. Only irradiated by 405-nm light with an exposed time of 180 ms but without developing (black lines). Stage 2. Developed by ethanol after exposing (green lines). Stage 3. Developed by $PdCl_2$ / ethanol solution after exposing (blue lines). (c) Comparing the average amplitude of four samples (i.e. PPO, PPOB, PPOP and PPOH-3) after developing in $PdCl_2$ / ethanol solution. Among them, the amplitude of the PPOH-3 film has the maximum value (122 nm). The error bars span a range of two standard deviations.

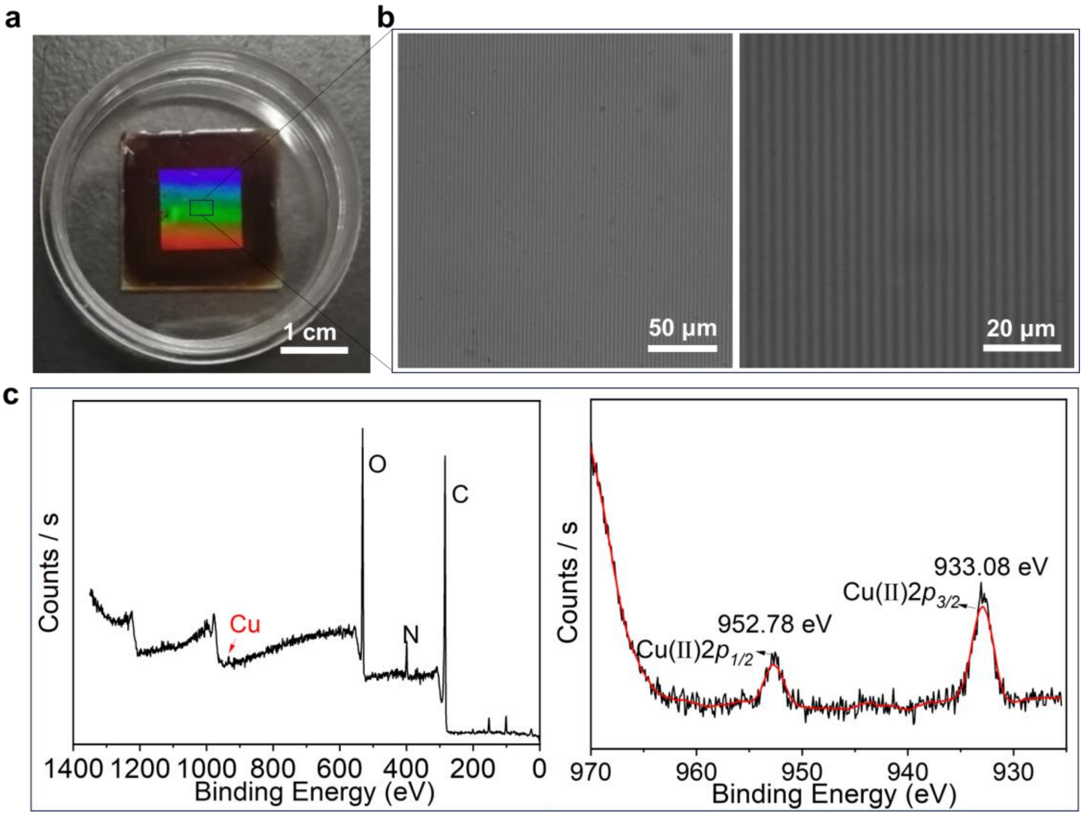

Supplementary Figure 28. PPOH-3 film of wrinkling patterns without random or cracked structures. (a) Photograph of the film after developing in ethanol containing $Cu(NO_3)_2$ followed by thermal treating with 300 ℃. (b) Optical images of the ordered wrinkles with a line width of 1.5 μm and spacing of 1.5 μm. (c) XPS spectra of the wrinkling film.

Section 5: Optical property of wrinkles on photosensitive polyamic acid films

Section 5.1 Diffraction simulations of wrinkles under out-of-plane rotation

The finite-difference time domain (FDTD) is used to simulate the diffraction property of the grating wrinkles (1.5-μm line width, 1.5-μm line spacing, and 122-nm amplitude). The wavelength range of the light source is set as 400-800 nm. Because the wavelength range of visible light (380-760 nm) is much smaller than the period of wrinkles designed (3 μm) in this paper, the wrinkles are polarization insensitive. The simulation mode is set as 2-dimensional (2D) to conserve computing resources. When the direction of source perpendicular to the wrinkles, the periodic boundary is set in transverse axis, and the perfectly matched layer (PML) boundary is set in optical propagation direction. The source incident from air layer on the top, and the diffraction information is corrected behind the source. The diffraction order is shifted dependent on the incident angle α. The smaller incident angle α, the higher intensity on 1st-order diffraction angle β.

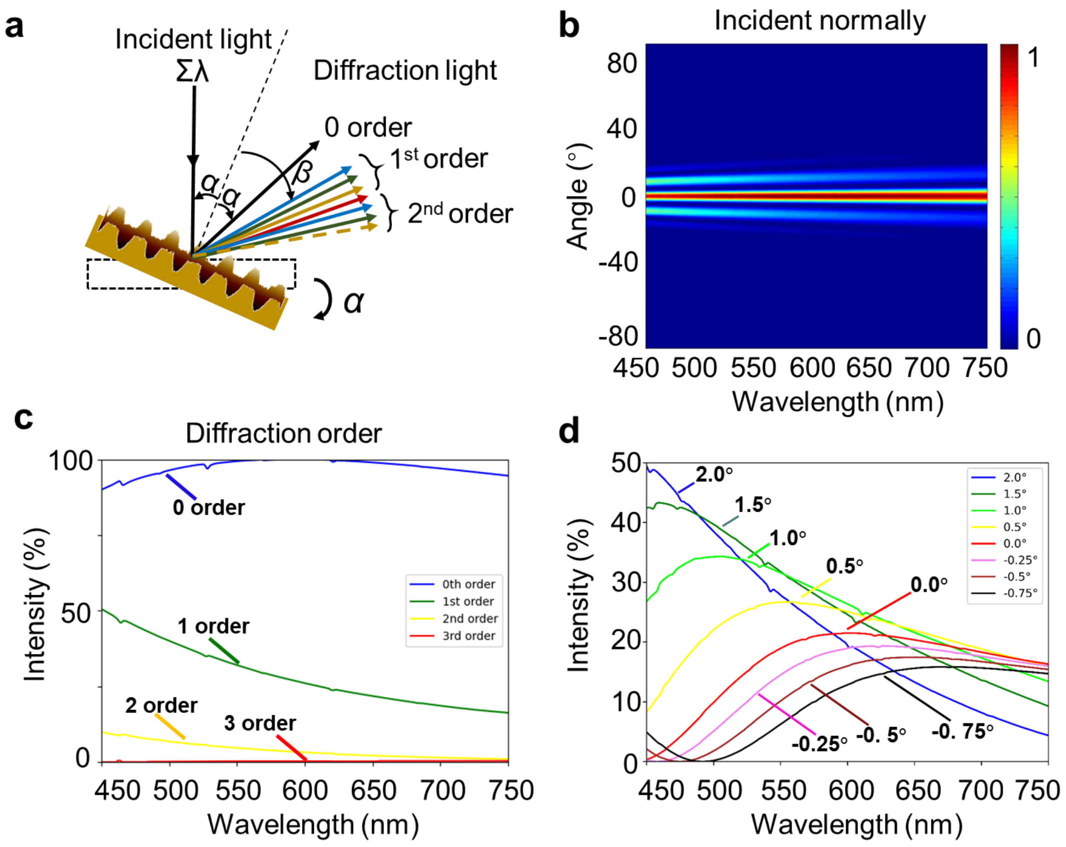

Supplementary Figure 29. The optical results of the grating wrinkles in simulation. (a) The diffraction schematic of grating wrinkles under out-of-plane rotating. The sample is illuminated by plane light vertically, and the observation direction perpendicular to the grating direction, i.e., $\alpha = 0°$, $\beta = 12.7°$, $\varphi = 0°$. The out-of-plane rotation will change the diffraction results of the sample under other conditions unchanged, i.e., $\alpha \neq 0°$, $\beta = 12.7° - \alpha$, $\varphi = 0°$. (b) Calculated normalizing electric field intensity results based on the wrinkles on PPOH-3 film (see in Supplementary Fig. 13c). (c) Calculated results of normalized diffraction order intensity based on the wrinkles on PPOH-3 film. (d) Calculated diffraction results under different incident angles α based on wrinkles on PPOH-3 film. Sum of detect angle and incident angle is 12.7°, i.e., $\alpha + \beta = 12.7°$. The negative angle α means the incident direction and the observation direction on the same side of normal line.

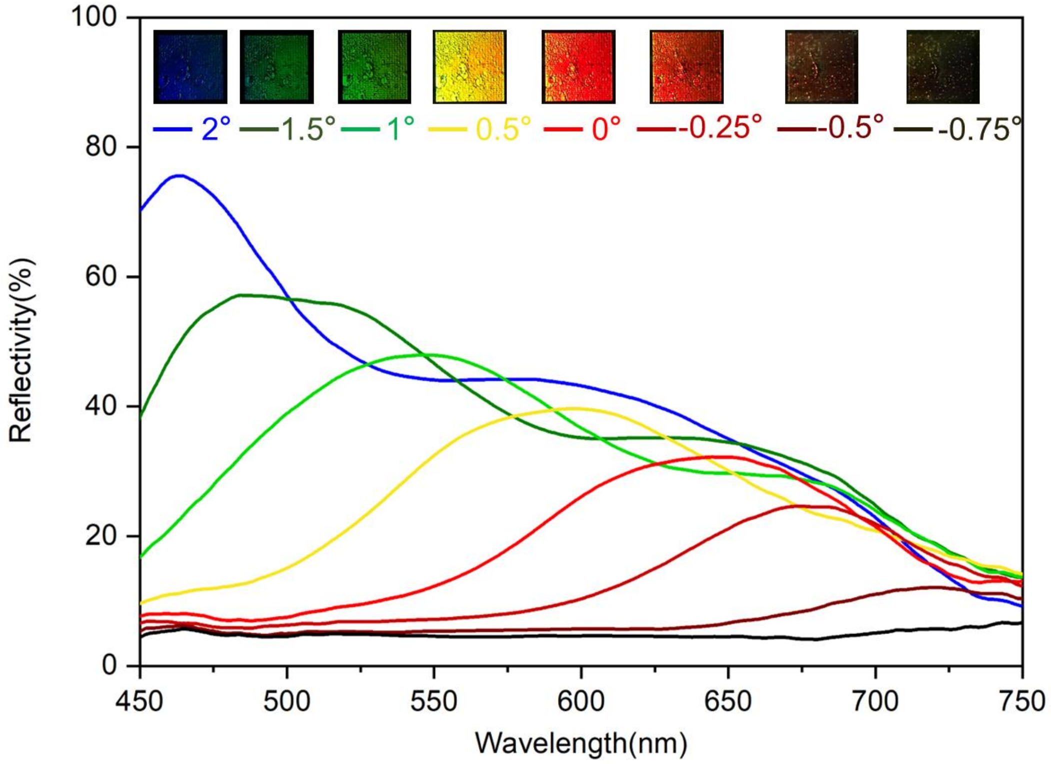

Supplementary Figure 30. 1st-order diffraction spectra in experiments. Diffraction results under different incident angles α based on Supplementary Fig. 13b. Inset, angle-dependent structural color images of wrinkles with a line width of 1.5 μm and a line spacing of 1.5 μm.

Supplementary Table 4. As the sum of incident angle α and diffractive / observation angle β is fixed (12.7°), the diffractive wavelength λ of the 1st-order diffraction by the wrinkles when the film is tilted with different angles α. The period D of grating wrinkles is 3 μm.

Variate of Bragg diffraction equation	Bragg diffraction equation: λ = D (sin β - sin α); D = 3 μm.							
	Actual arguments							
Diffractive angle β (°)	10.7	11.2	11.7	12.2	12.7	12.95	13.2	13.45
Incident angle α (°)	2	1.5	1	0.5	0	-0.25	-0.5	-0.75
sin β – sin α	0.151	0.168	0.185	0.203	0.220	0.228	0.237	0.246
Theoretical diffractive wavelength λ (nm)	453	504	555	609	660	684	711	738

Section 5.2 Diffraction simulations of wrinkles under in-plane rotation

With FDTD method, the light intensity of the 1st-order diffraction from high to low as the wrinkles (line width: 1.5 μm; line spacing: 1.5 μm; amplitude: 122 nm) are rotated in-plane under vertical irradiating with plane white light. The light intensity reaches the maximum in the direction of the reflection plane perpendicular to the wrinkles. The intensity of the in-plane rotating wrinkles is detected at the 1st-order diffraction angle.

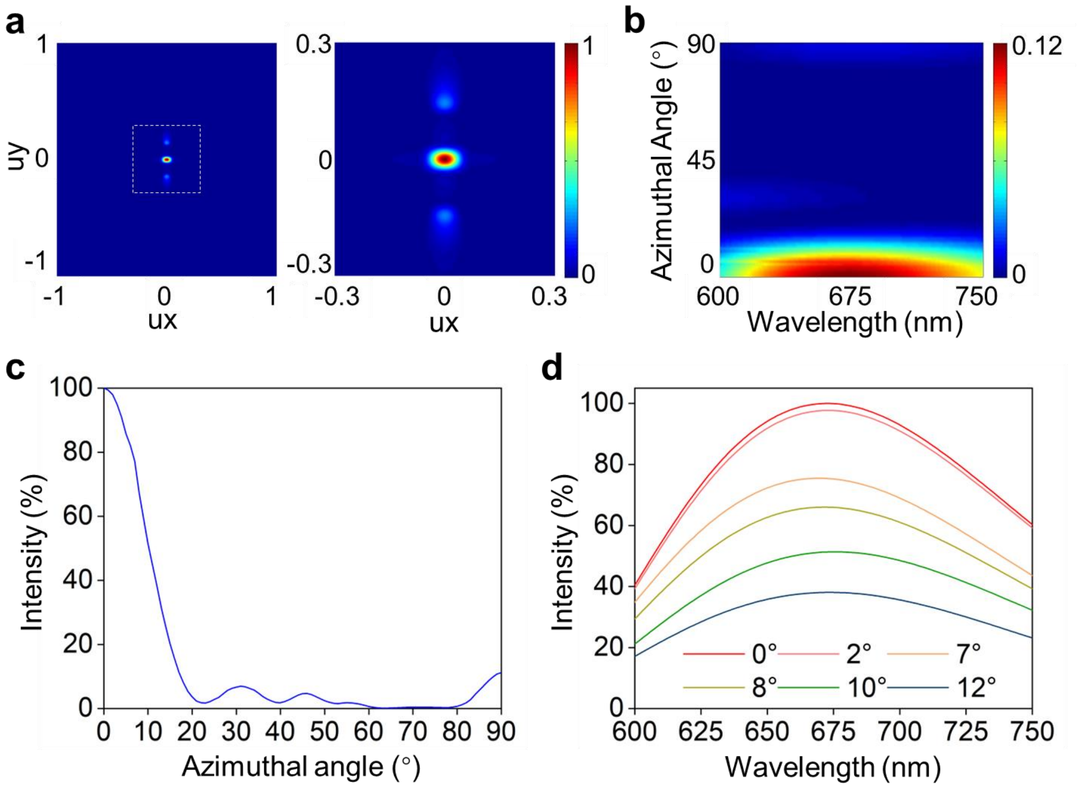

Supplementary Figure 31. Simulation and experiment optical response of the wrinkles with a line width of 1.5 μm and a spacing of 1.5 μm under in-plane rotating. (a) Calculated diffraction results under different azimuthal angles φ based on the demonstration in Fig. 3a. The ux and uy represent the directional cosine values in the x direction and y direction, respectively. (b) The calculated 1st-order diffraction intensity varies with the azimuthal angle. Remaining other conditions, rotating in-plane can change the diffraction result directly of the sample, i.e., $\alpha = 0°$, $\beta = 12.7°$, $\varphi \neq 0°$. (c) Normalized 1st-order diffraction (the diffraction angle is 12.7°) intensity with in-plane rotating. (d) Normalized intensity at specific angle of in-plane rotation (the maximum value at 0° is marked as 1). The specific angles are 0°, 2°, 7°, 8°, 10°, and 12°, respectively.

Section 6: Patterned wrinkling photosensitive polyamic acid film

Section 6.1 Dot matrix patterns and wrinkles patterns

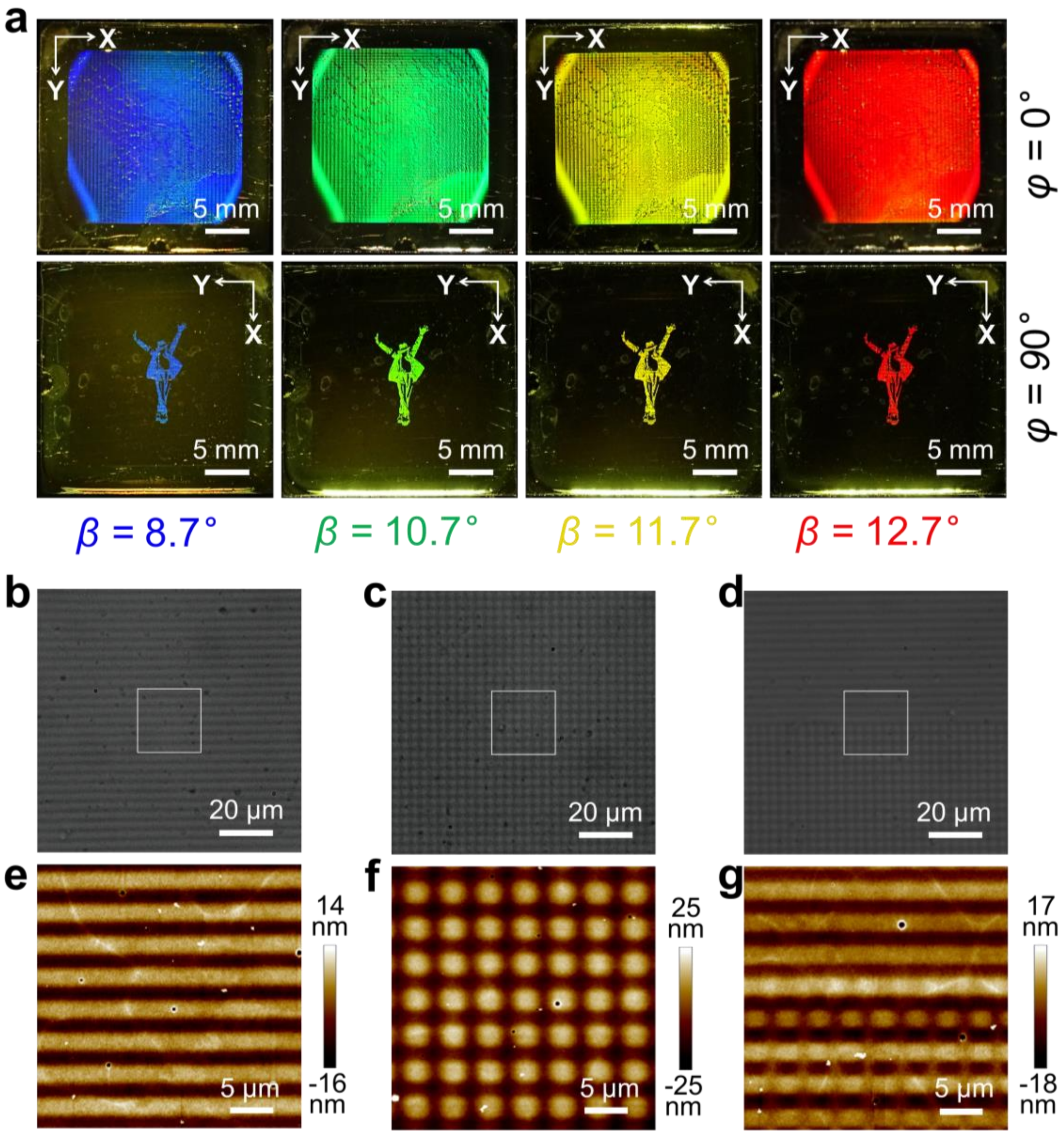

Supplementary Figure 32. Encryption patterns constructed by dot matrix and wrinkles. (a) Angle-dependent structural color images of the Michael Jackson (MJ) pattern and its background that constructed with wrinkles and dot matrix structures, respectively. (b, c, d) Optical microscope images and (e, f, g) 3D AFM images of wrinkles and dot matrix structures.

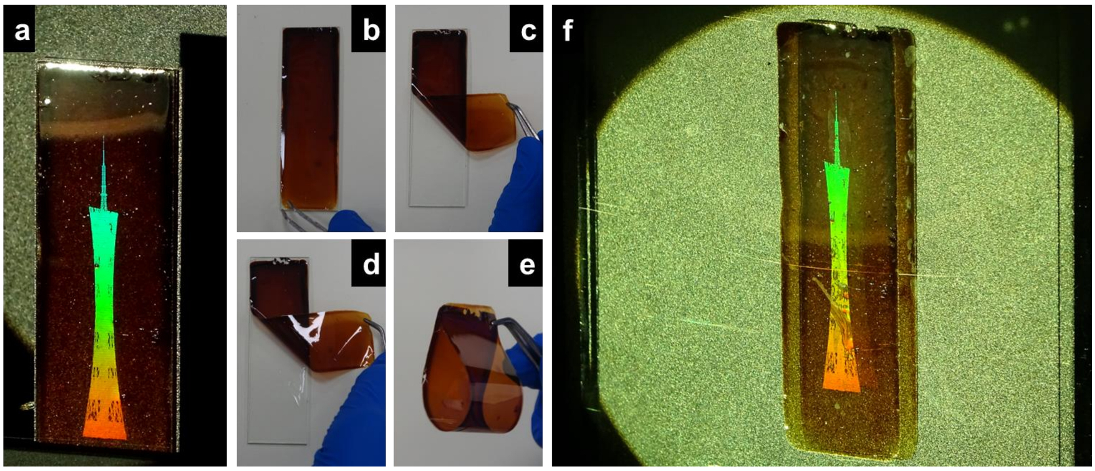

Supplementary Figure 33. Transferability of the PI film containing wrinkling patterns. (a). An optical image of wrinkled polyimide film on glass slide shows the pattern of Canton Tower. (b-e). Release process of the wrinkled polyimide film from glass substrate by peeling with tweezer. (f) The film was then deposited onto another glass substrate. No obvious change of the pattern as well as homogeneity of the Canton Tower, indicating the film can be transferred freely from one substrate to the other without changing its optical information.

Section 6.2 Patterning four-plex wrinkles

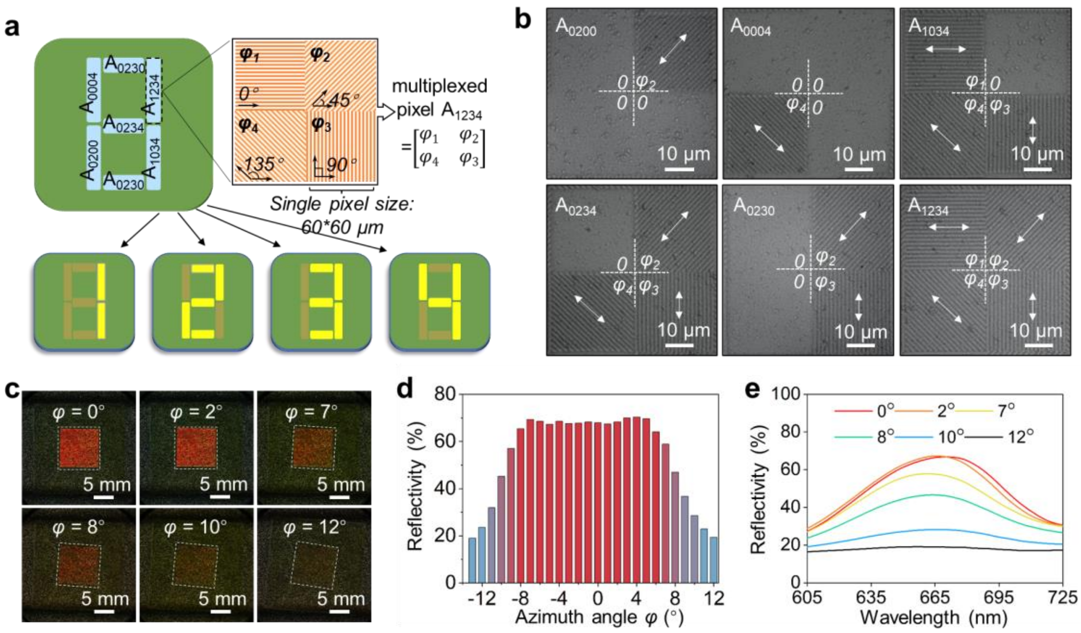

Supplementary Figure 34. Optical properties of PPOH-3 film with designed wrinkles. (a) Schematic image of utilizing six multiplexed pixels to design switchable images of numbers "1" to "4" based on the rotation of φ by 45°. Each multiplexed pixel could be described as a 2 × 2 matrix, which contained four elements of 60-μm-sized squares composed of independent and periodic arrays of wrinkles with defined directions (φ: φ_1 = 0°, φ_2 = 45°, φ_3 = 90° and φ_4 = 135°). The multiplexed pixels were utilized to construct "number 8" with seven short lines. The multiplexed pixels in the seven short lines (A_{0200}, A_{0004}, A_{1034}, A_{0234}, A_{0230}, and A_{1234}) were defined as the linear sums of corresponding independent pixels in presenting numbers 1 to 4. For example, if the line was only used in one number, its pixel contained only one wrinkled element and the areas of the other three elements are empty, and if the short line was shared by different numbers, such as 2 and 3, then the pixel could be present as the linear sums of that only for 2 and 3, namely $\begin{bmatrix} 0 & \varphi_2 \\ 0 & \varphi_3 \end{bmatrix} = \begin{bmatrix} 0 & \varphi_2 \\ 0 & 0 \end{bmatrix} + \begin{bmatrix} 0 & 0 \\ 0 & \varphi_3 \end{bmatrix}$. Altogether, six

multiplexed pixels were utilized to present numbers 1 to 4. (b) Optical microscopy images of the six multiplexed pixels with wrinkled structures on PAA film. (c) Optical image of the PAA film with red diffraction light under different azimuthal angles φ. (d) The maximum reflectivity of the red diffraction light for different azimuthal angle φ. (e) Spectral reflectance curves of the red wrinkling area at a specific angle of in-plane rotation (i. e. 0°, 2°, 7°, 8°, 10°, 12°).

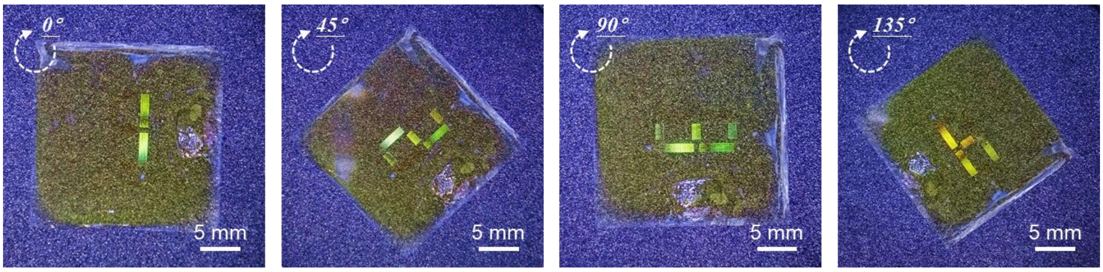

Supplementary Figure 35. Wrinkling patterns constructed by multiplexed pixels are obtained on PPOH-3 film after developing with methanol containing $PdCl_2$.

Section 6.3 Patterning eight-plex wrinkles

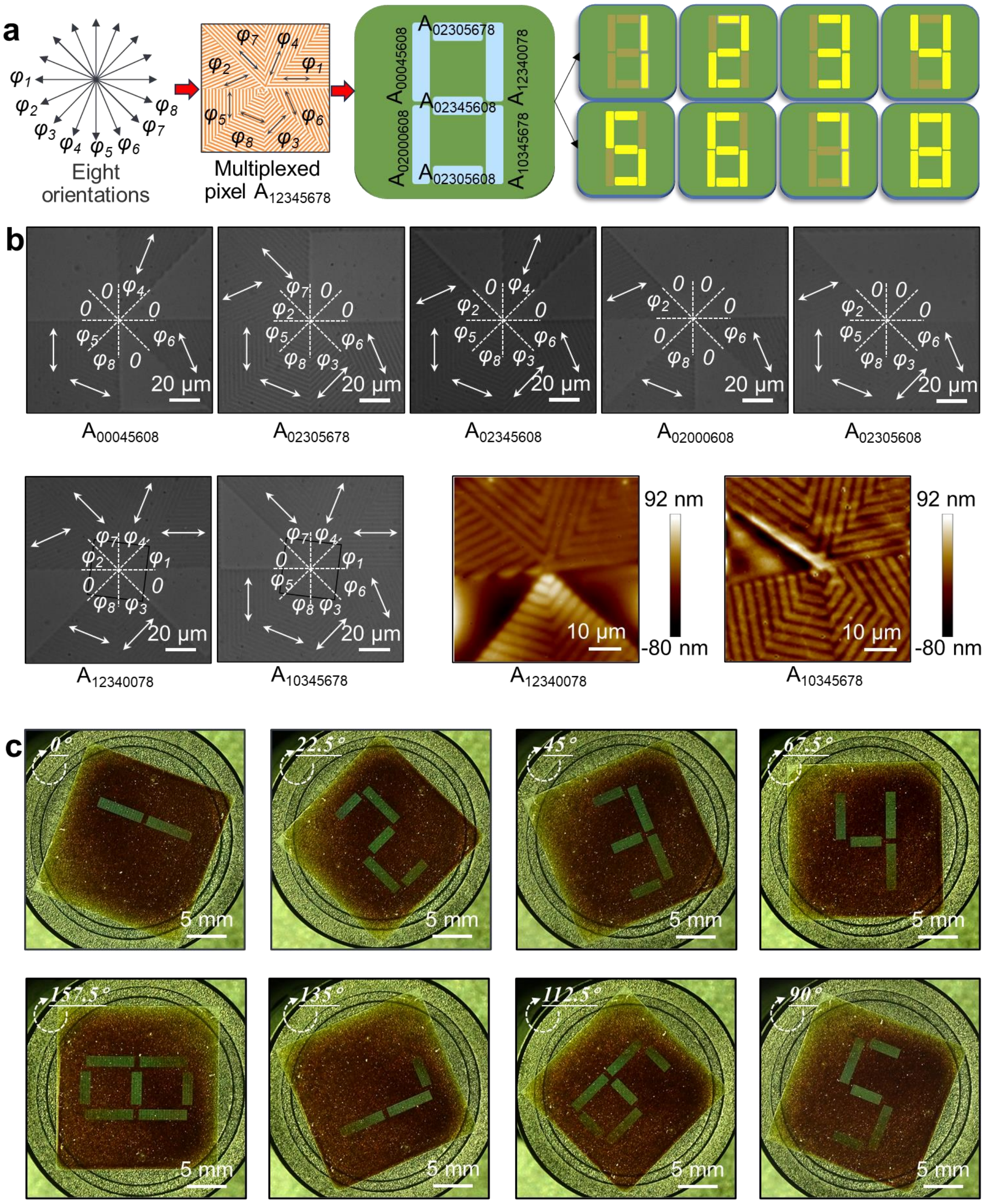

Supplementary Figure 36. Rational design of wrinkles for optical encryption with eight switchable patterns at one place. (a) Schemes of utilizing seven multiplexed pixels to design switchable images of numbers “1” to “8” based on the rotation of φ by 22.5°. All multiplexed pixels were based on the multiplexed pixel $A_{12345678}$ constructed with eight different elements that have same appearance of isosceles right

triangle but anisotropic wrinkling structures with defined directions (φ: φ_1 = 0°, φ_2 = 22.5°, φ_3 = 45°, φ_4 = 67.5°, φ_5 = 90°, φ_6 = 112.5°, φ_7 = 135° and φ_8 = 157.5°). The multiplexed pixels were utilized to construct “number 8” with seven short lines. The multiplexed pixels in the seven short lines ($A_{00045608}$, $A_{02305678}$, $A_{02345608}$, $A_{02000608}$, $A_{02305608}$, $A_{12340078}$ and $A_{10345678}$) were defined as the linear sums of corresponding independent pixels in presenting numbers 1 to 8. For example, if the line was only used in one number, its pixel contained only one wrinkled element and the areas of the other seven elements are empty, and if the short line was shared by different numbers, such as 4, 5, 6 and 8, then the pixel could be present as the linear sums of that only for 4, 5, 6 and 8, namely $A_{00045608}$= $A_{0004000}$ + $A_{00005000}$ + $A_{00000600}$ + $A_{00000008}$. Altogether, seven multiplexed pixels were utilized to present numbers 1 to 8. (b) Optical microscopy images of utilizing seven types of multiplexed pixels ($A_{00045608}$, $A_{002305678}$, $A_{02345608}$, $A_{02000608}$, $A_{00230560}$, $A_{12340078}$ and $A_{10345678}$) to fabricate encrypted image including eight switchable numbers 1 to 8. The AFM topological image of pixels $A_{12340078}$ and $A_{10345678}$ were presented. (c) Optical images of the numbers “1” to “8” on wrinkled polyimide film at different azimuthal angle φ (0°, 22.5°, 45°, 67.5°, 90°, 112.5°, 135°, and 157.5°).

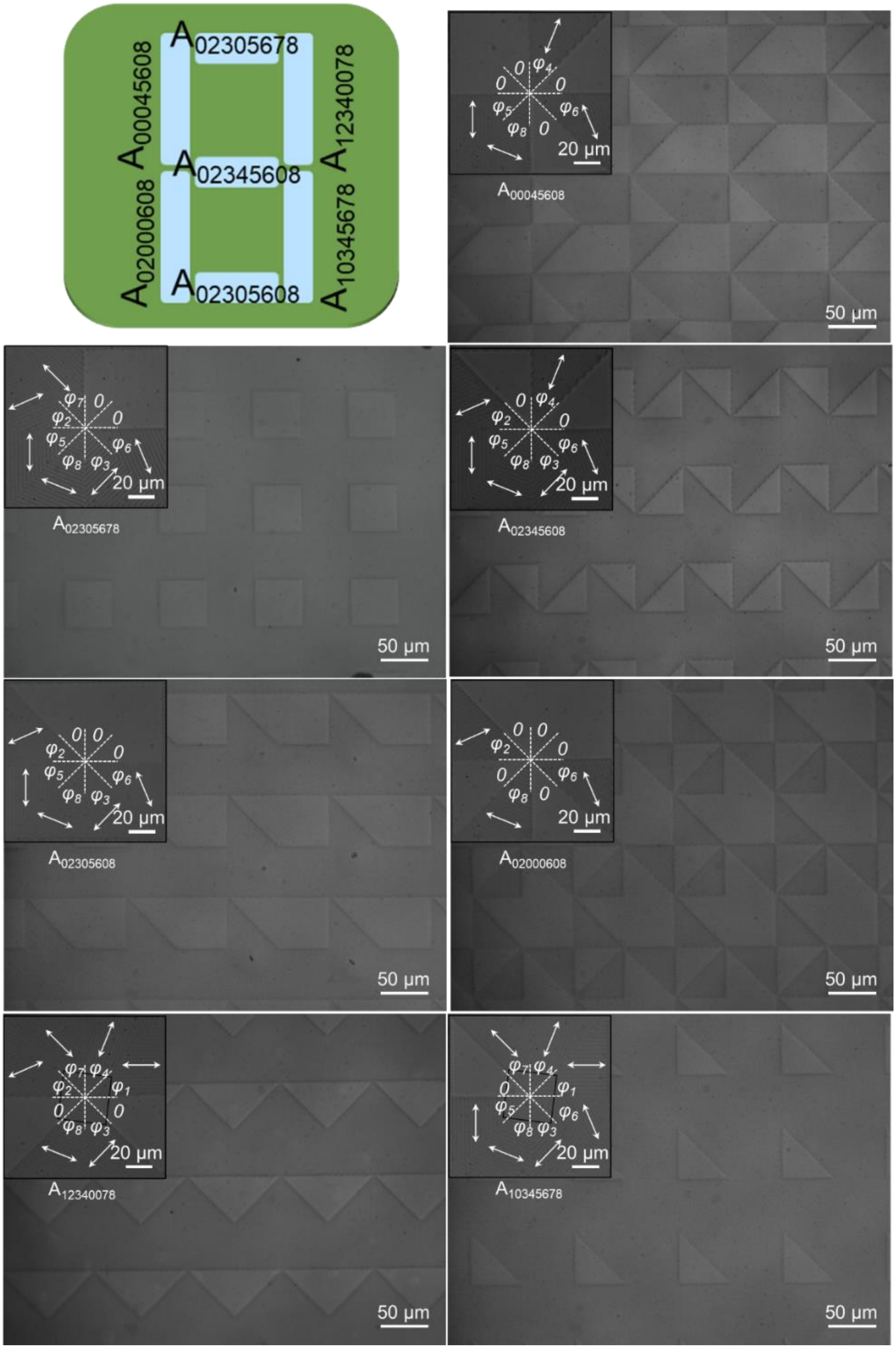

Supplementary Figure 37. Optical microscopy image of the morphology of the multiplexed pixels with wrinkled structures of eight switchable patterns over large areas.

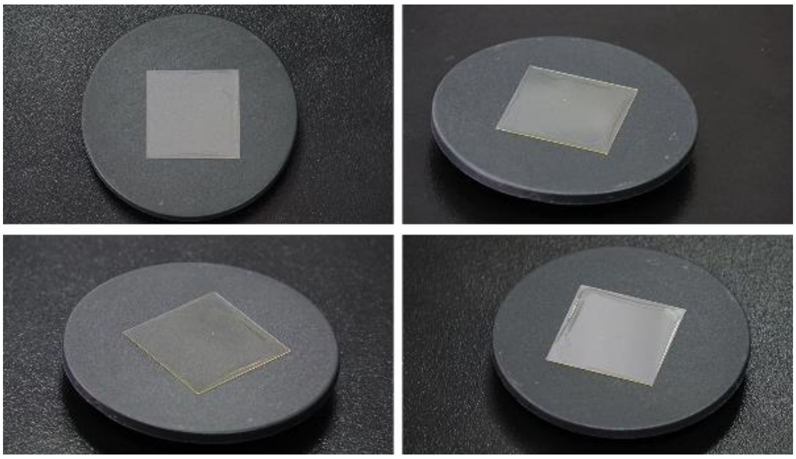

Supplementary Figure 38. Photographs of wrinkled PPOH-3 sample captured from random angles.

Section 7: Constructing copper circuit on the wrinkles template

Section 7.1 Coefficient thermal expansion and dielectric properties analysis

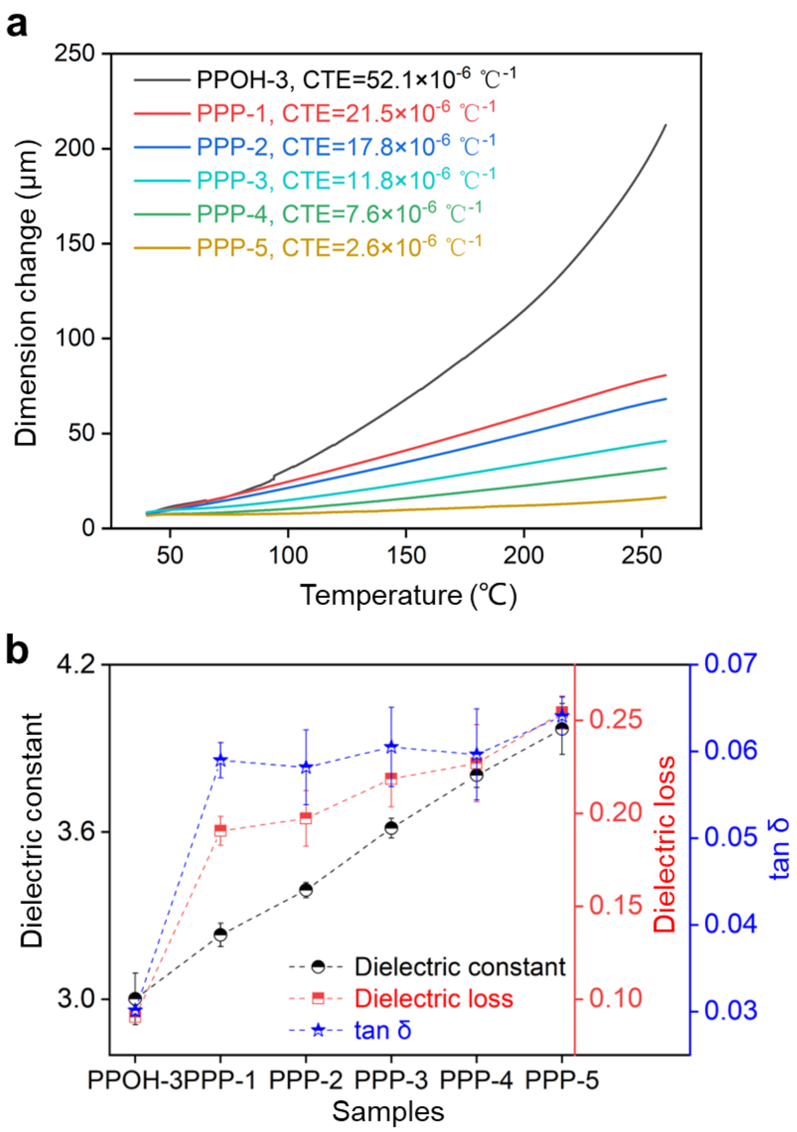

Supplementary Figure 39. Coefficient thermal expansion and dielectric properties of polyimide films. (a) The coefficient thermal expansion of 6 kinds of polyimide films with different rigid chains (see in Supplementary Tab. 1), the coefficient thermal expansion of imidized PPP-2 is 17.8×10^{-6} / °C, which is close to the copper. (b) Electrical properties of six kinds of polyimide films at 10 GHz and room temperature. The error bars span a range of two standard deviations.

Section 7.2 The characterization analysis of fine copper lines with a 13-μm width and an 87-μm spacing

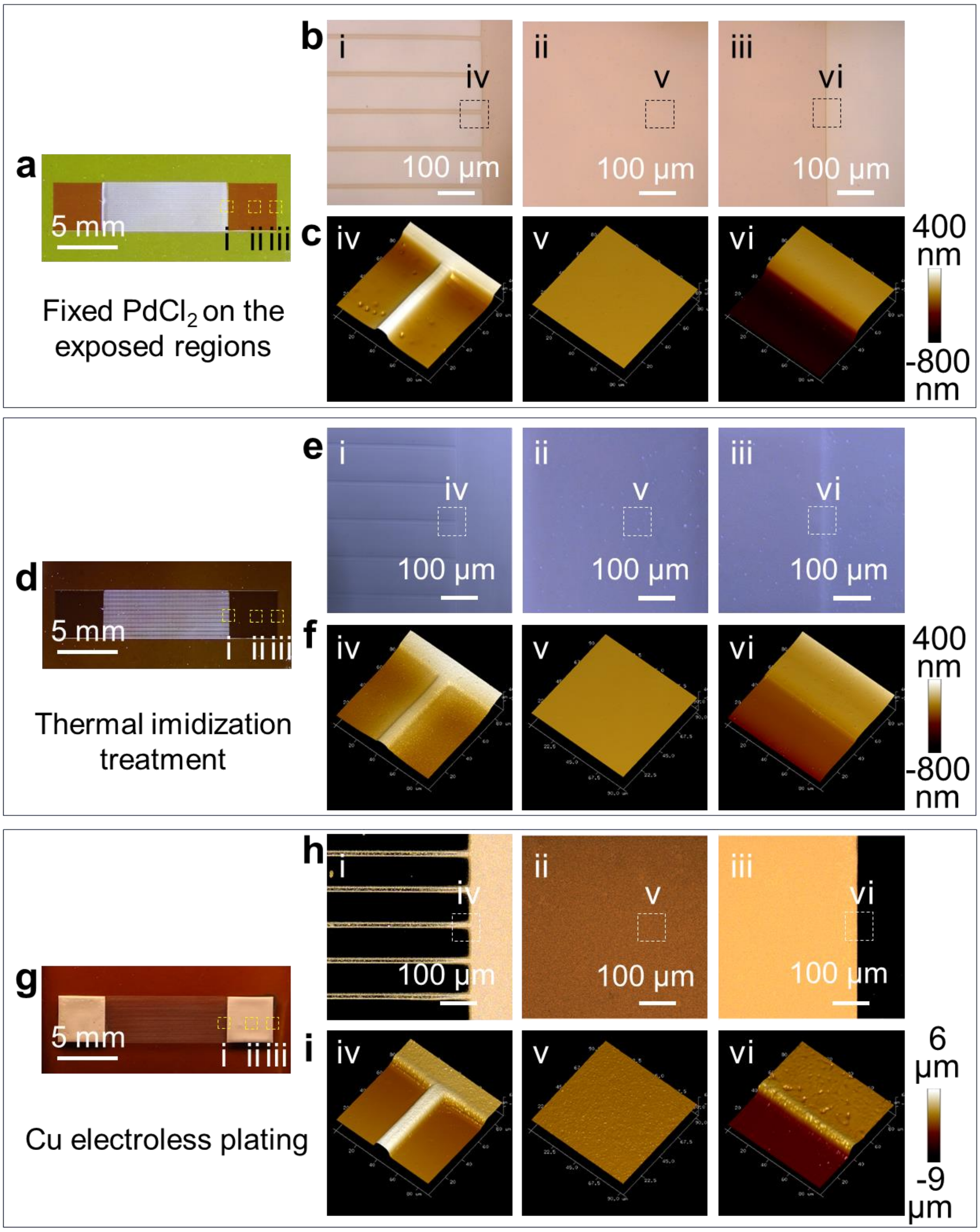

Supplementary Figure 40. Manufacturing copper circuit with a 13-μm width and an 87-μm spacing by addition method. (a, b, c) After exposing, the photosensitive PPP-2 film is developed by $PdCl_2$ / ethanol solution, and the exposed position formed the wrinkles and adsorbed $PdCl_2$. (d, e, f) The photosensitive PPP-2 film adsorbed $PdCl_2$

is imidized to polyimide by programmed heating；(g, h, i) The exposed areas are metalized by electroless copper plating. (i is the junction between the exposed lines, i.e. wrinkles, and the exposed square; ii is the center of the exposed square; iii is the junction between the exposed square and the unexposed area of the film). a, b, d, e, g and h, optical images. c, f and i, AFM images.

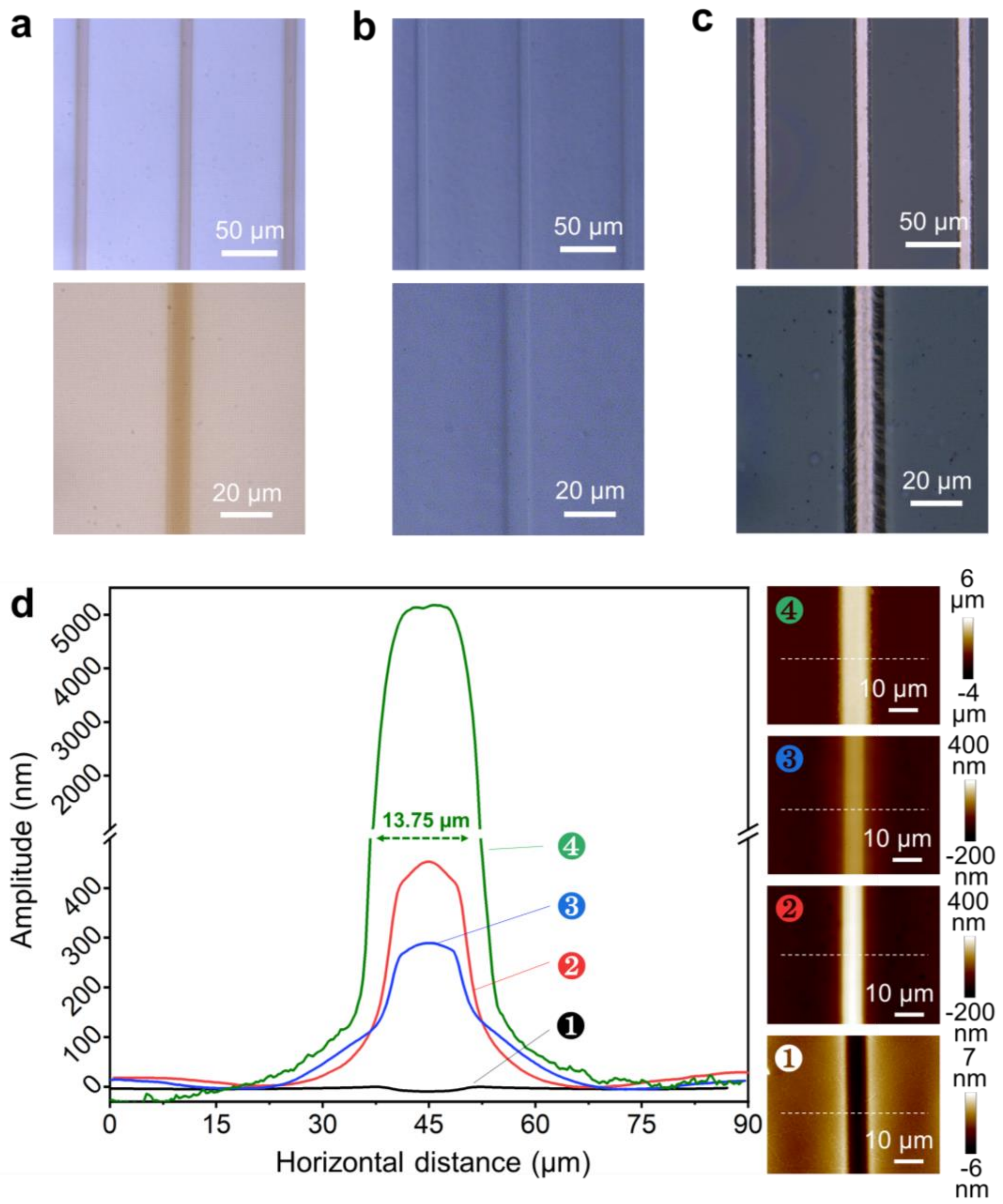

Supplementary Figure 41. The manufacturing process of fine copper lines with a 13-μm width and an 87-μm spacing on polyimide (imidized PPP-2) film. In situ optical images of the wrinkles after (a) adsorbing of the $PdCl_2$, (b) thermal acylation, and (c) electroless copper plating. (d) In situ AFM images (right) of the surface profiles evolution (left) of the film during the metal patterning process. 1, After patterning; 2, After developing in $PdCl_2$ / ethanol solution; 3, Thermal imidization; 4, Electroless plating of copper.

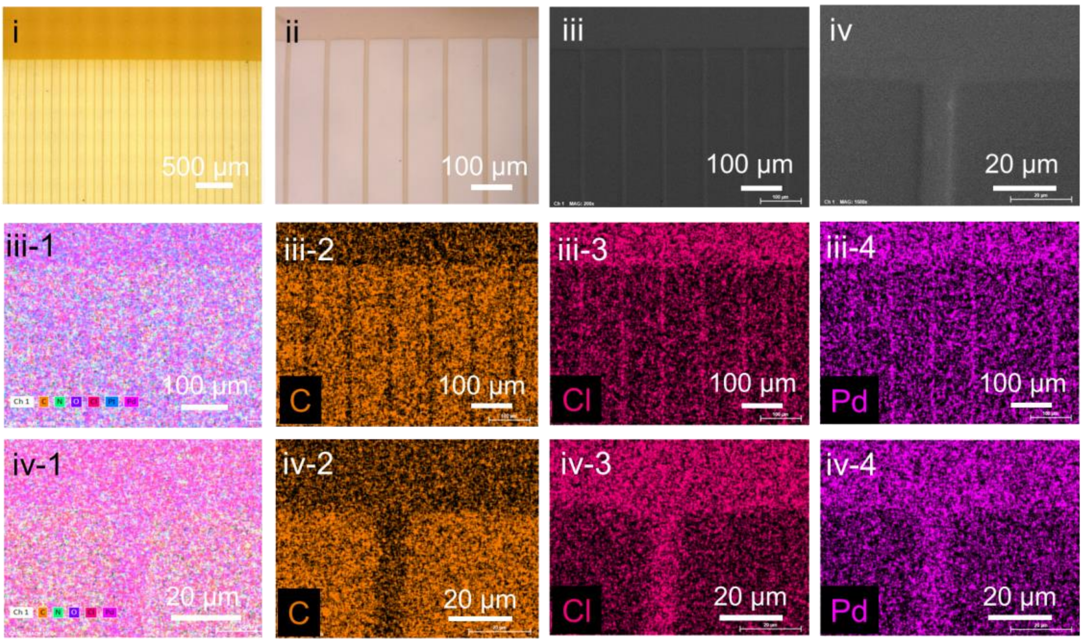

Supplementary Figure 42. Analysis of surface components of PPP-2 film adsorbed $PdCl_2$. (i, ii) The optical micrographs, (iii, iv) SEM images, and (v-xii) EDS images of the photosensitive polyamide acid film after adsorbing $PdCl_2$.

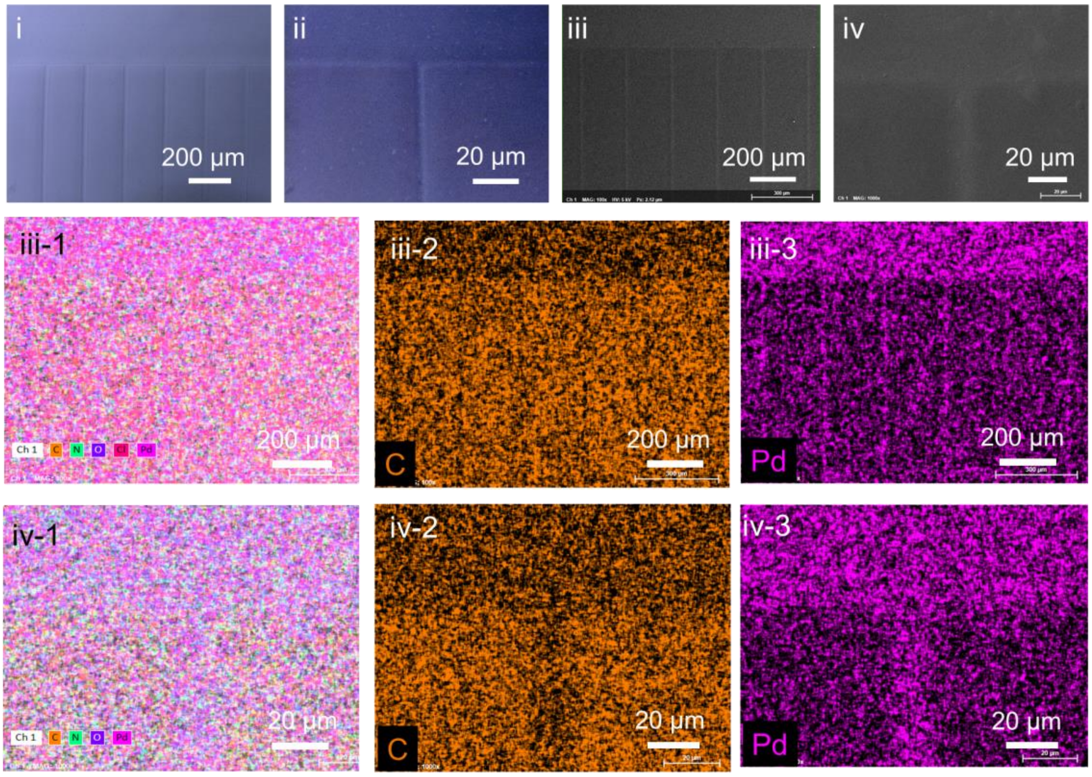

Supplementary Figure 43. Analysis of surface components of polyimide (imidized PPP-2) films adsorbed $PdCl_2$ after thermal imidization. (i, ii) The optical micrographs, (iii, iv) SEM images, and (v-x) EDS images of photosensitive polyimide film with Pd (0) on surface after thermal acylation.

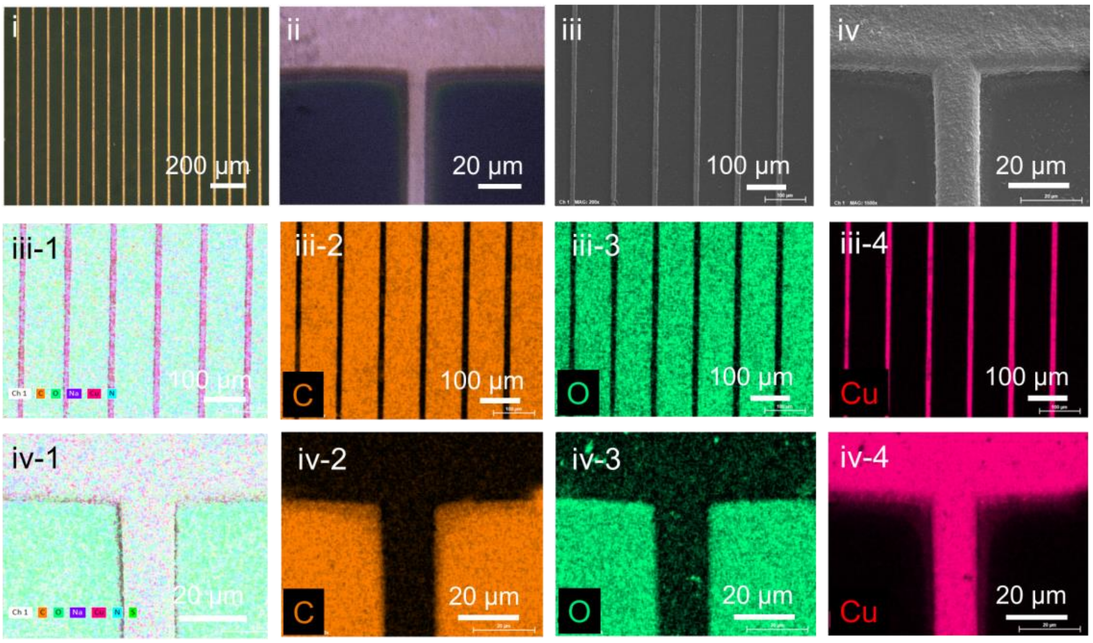

Supplementary Figure 44. Analysis of surface components of polyimide (imidized PPP-2) film after electroless copper plating. (i, ii) The optical micrographs, (iii, iv) SEM images, and (v-xii) EDS images of the wrinkles on the photosensitive polyimide film after electroless copper plating.

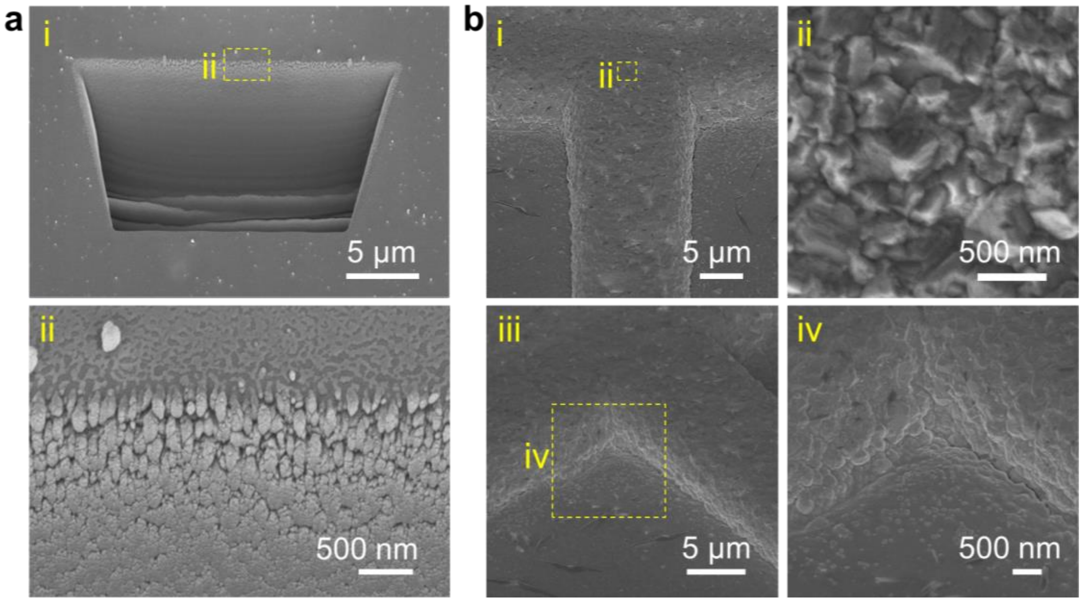

Supplementary Figure 45. The SEM images of photosensitive polyimide (imidized PPP-2) film containing Pd(0) on the surface before and after electroless copper plating. (a) Interface structure of the polyimide film containing Pd(0) after focused ion beam cutting; (b) Details of copper wire (i, ii) on the film surface and (iii, iv) the connection area between the copper wire and the film interface.

Section 7.3 The characterization analysis of fine copper lines with a 13-µm width and a 17-µm spacing

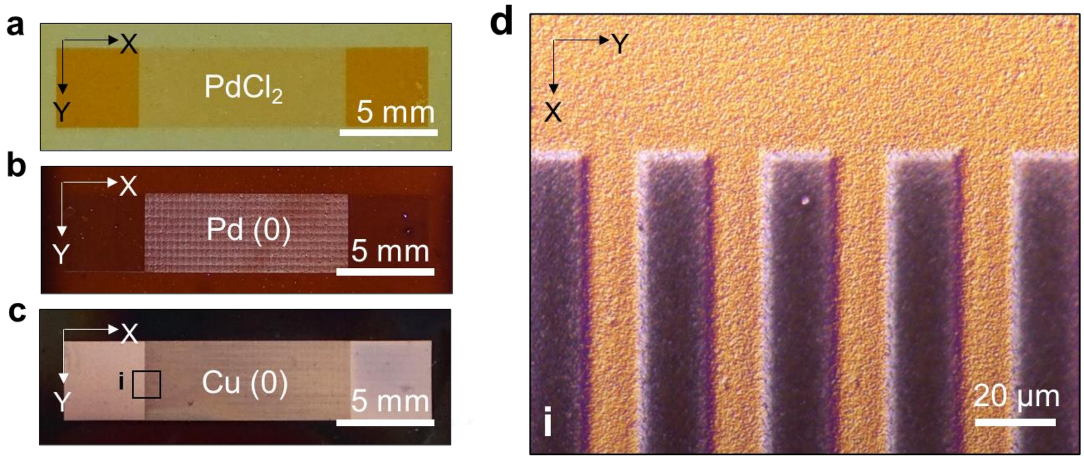

Supplementary Figure 46. The manufacturing process of copper circuit with a 13-µm width and a 17-µm spacing. The patterned area images after (a) developing in $PdCl_2$ / ethanol solution, (b) thermal imidization at 300 °C, and (c) electroless plating of copper. (d) Optical microscopy images of the connect area between copper square and copper lines.

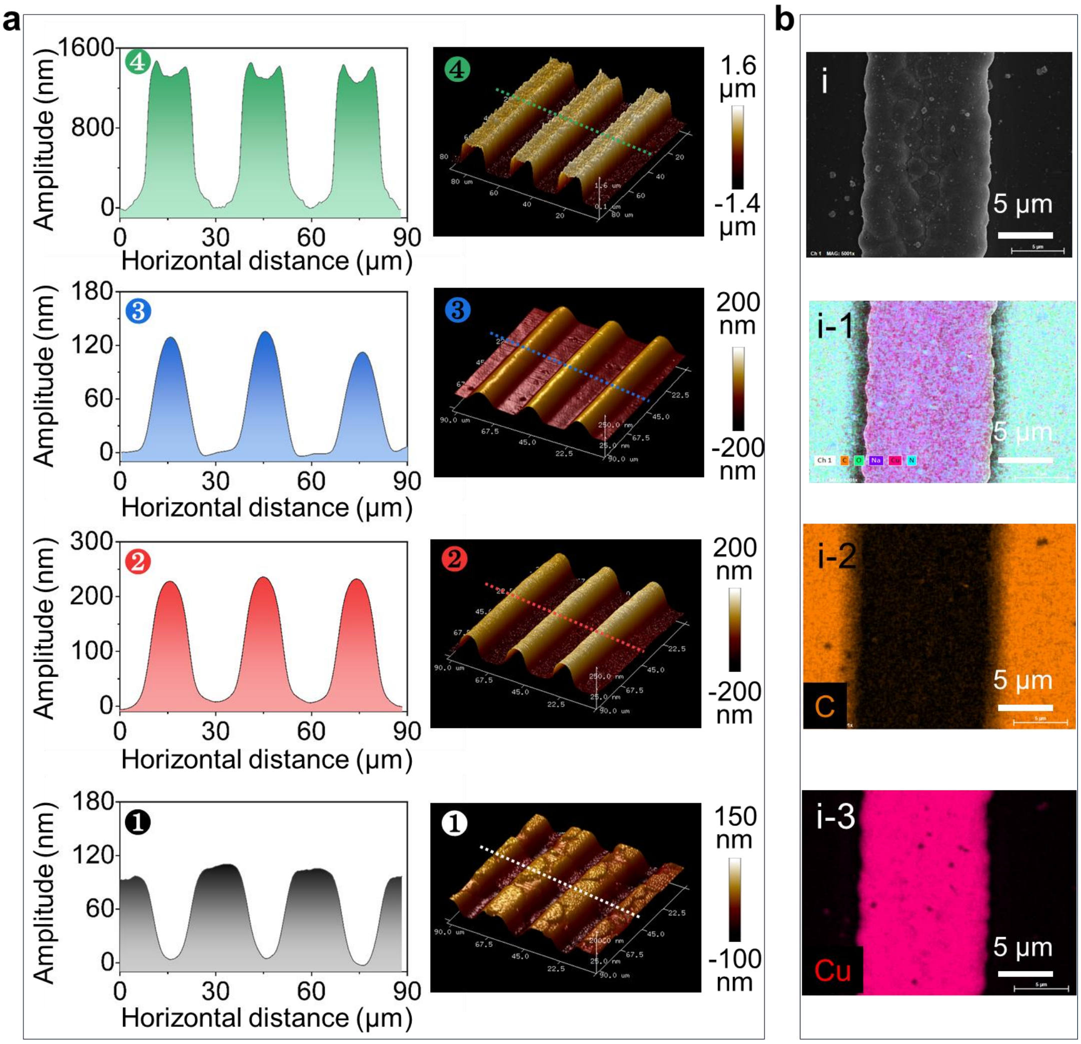

-

Supplementary Figure 47. The manufacturing process of fine copper lines with a 13-μm width and a 17-μm spacing on polyimide (imidized PPP-2) film. (a) In situ AFM images (right) of the surface profiles evolution (left) of the film during the metal patterning process. 1, After patterning; 2, After developing in $PdCl_2$ / ethanol solution; 3, Thermal imidization; 4, Electroless plating of copper. (b) The (i) SEM image and (i-1, 2, 3) EDS images of the copper lines on film.

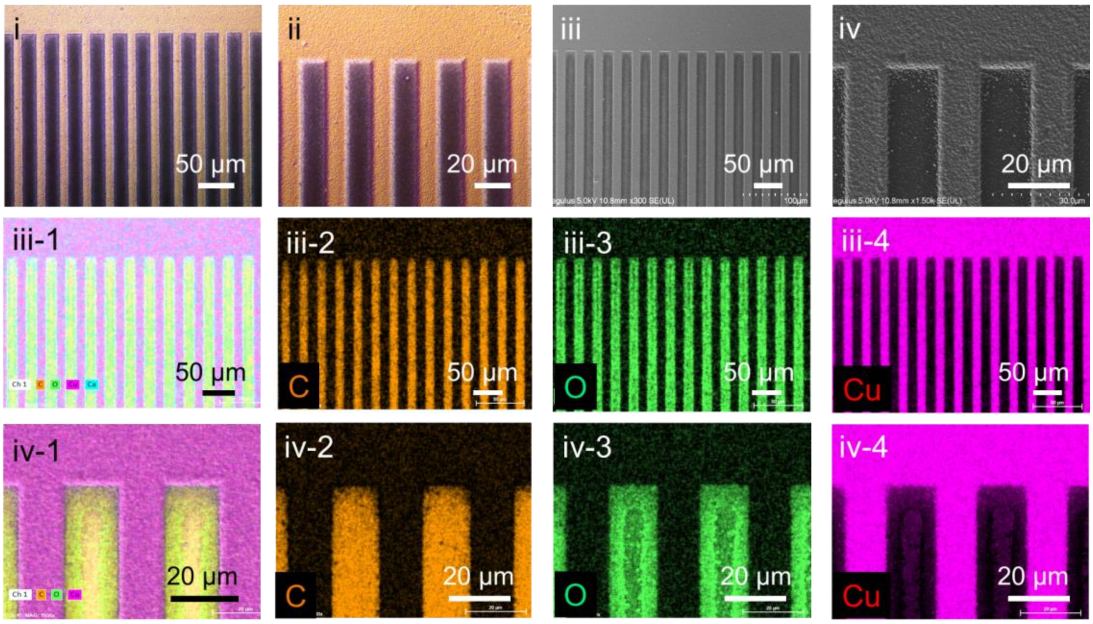

Supplementary Figure 48. Analysis of surface components of polyimide (imidized PPP-2) film after electroless copper plating. (i, ii) The optical micrographs, (iii, iv) SEM images, and (v-xii) EDS images of the wrinkles on the photosensitive polyimide film after electroless copper plating.

Section 7.4 Mechanical stability of copper circuit

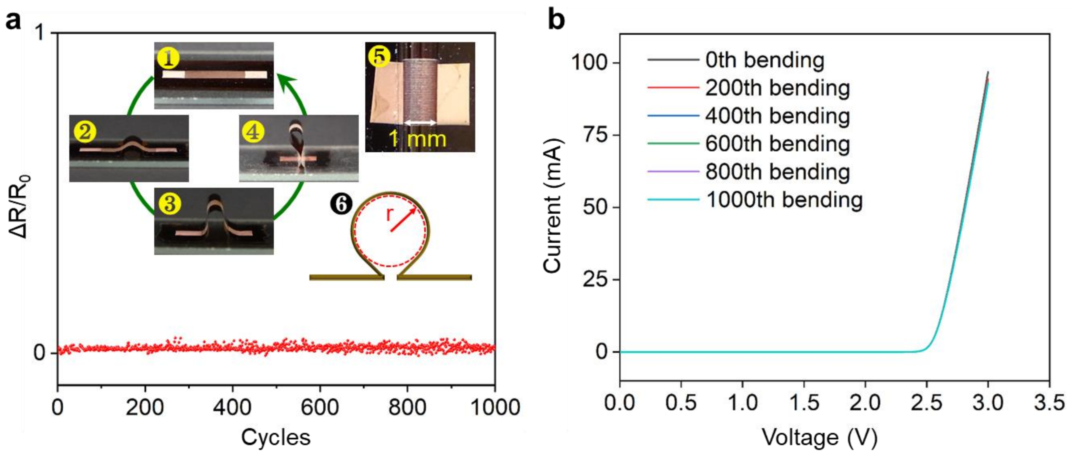

Supplementary Figure 49. Bending resistance of copper circuit. (a) Normalized resistance of copper patterns after repeated bending of the copper circuit with a radius of curvature (r) of 0.5 mm. (b) The I-V curve of the copper circuit under repeated bending at an r of 0.5 mm.

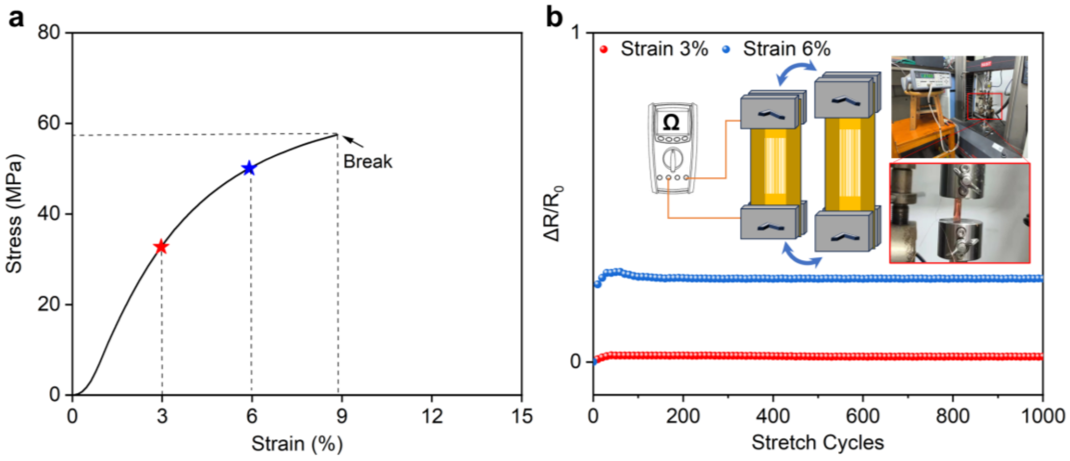

Supplementary Figure 50. Stretching resistance of copper circuit. (a) Stress-strain curve of the flexible IC carrier board. (b) Normalized resistance of copper patterns after repeated stretching with the strain of 3% and 6%, respectively.

Supplementary Table 5. Comparison results of the typical parameters of the copper circuit in this work with industry standards for commercialization.

Content	This Work	Typical parameter	Standard document
Thickness of substrate (*H*)	~ 12 μm	10 μm ~125 μm	PRC National Standard GB/T 13555-2017
The dielectric constant of the substrate	3.3	2.8 ~ 3.9	IPC-2221A
Coefficient thermal expansion (CTE) of substrate	17.8×10^{-6} / ℃	5.0×10^{-6} / ℃ ~ 15×10^{-6} / ℃	IPC-2221A
Line width of wire (*W*)	13 μm	$W < 2H$	IPC-2221A
Line spacing of the wire	17 μm	0.1 mm ~ 0.13 mm	IPC-2221A
Line thickness of wire (*h*)	1.8 μm	0.6 μm ~ 2.5 μm at electroless copper plating	IPC-2221A
The electric conductivity of the wire	1.76×10^{-8} Ω·m	1.62×10^{-8} Ω·m ~1.81×10^{-8} Ω·m	IPC-4562
Adhesion stability of wire	5B	0B ~ 5B	ASTM D3359-09
Bending resistance of wire	1000 bending without failure at a curvature radius of 0.5 mm	≥400 bending without failure at a curvature radius of 2.0 mm	PRC National Standard GB/T 13555-2017
Roughness of copper foil	~ 56.5 nm	< 0.43 μm	IPC-4562

Section 8: Supplementary animation demonstration

Section 8.1 Supplementary Movie 1

Supplementary Movie 1 shows the patterns changing of PAA film containing both dot matrix and wrinkles under horizontal rotation. The MJ image and its circumambient part are constructed with dot matrix and wrinkles, respectively. The dot matrix along the y-axis has the same period as the ambient wrinkles, so compared with the wrinkles, the dot matrix exhibits structural color independent of the azimuthal angle φ (Supplementary Fig. 22a). When $\varphi = 0°$, the image of MJ that has the same structural color as the ambient wrinkles showed that cannot be recognized. And when $\varphi = 90°$, the part occupied by wrinkles become transparent, meanwhile the dot matrix along the x-axis still has a fixed period making the MJ image show vivid structural colors. The wrinkled patterns on the film show structural color dependent on the viewing angle β, and the good monochromaticity of both patterns are observed under the corresponding viewing angle.

Section 8.2 Supplementary Movie 2

Supplementary Movie 2 shows the switchable images of the four numbers from “1” to “4” without crosstalk on the PAA film under horizontal rotation (see in Fig. 3). The azimuth angle φ between the two adjacent images is 45°.

Section 8.3 Supplementary Movie 3

Supplementary Movie 3 shows the switchable images of the eight numbers from “1” to “8” without crosstalk on the polyimide film under horizontal rotation (see in Extended Data Fig. 6c). The azimuth angle φ between the two adjacent images is 22.5°.

Section 8.4 Supplementary Movie 4

Supplementary Movie 4 shows the adhesion stability of the copper lines of the copper circuit by Scotch tape adhesion test. The 3M pressure-sensitive tape is pasted on the copper patterns by a roller with 2 kg weight and then peeled quickly. The clean surface of the tape indicates that the adhesion stability of the copper patterns on the polyimide film has reached the highest adhesion classification (5B) of the American Society of Testing Materials (ASTM) standard D3359 tape test method.

Section 8.5 Supplementary Movie 5

Supplementary Movie 5 shows the high conductivity of the copper circuit through a simple conductive circuit device composed of a 3 V power supply, a customized luminescent plate covered by the school badge pattern of Sun Yat-sen University, and the copper circuit. Whether it is continuous or intermittent current through, there is no damage to the copper circuit.

Section 8.6 Supplementary Movie 6

Supplementary Movie 6 shows the high reliability of the copper circuit through the thermal imaging of the simple conductive circuit device. Under long-time electrification (30 minutes), the copper circuit has no significant temperature change.

Supplementary References

1. Liu, J. et al. Metal conductive surface patterning on photoactive polyimide. *Adv. Funct. Mater.* **27**, 1701674 (2017).

2. Kuo, H. M., Hsu, Y. T., Wang, Y. W., Lee, G. H. & Lai, C. K. The π-π interactions enhanced in salicylaldimines and salicylaldazines. *Tetrahedron.* **71**, 7729-7738 (2015).

3. Wei, L. H. 2,6-Dimethylpyridinium 2,4-dihydroxy-benzoate 2,6-dimethylpyridine solvate. *Acta Cryst.* **E63**, o368-o369 (2007).

4. Vijayalakshmi, A., Vidyavathy, B., Peramaiyan, G. & Vinitha, G. Synthesis, growth, structural and optical studies of a new organic three-dimensional framework: 4-(aminocarbonyl) pyridine 4-(aminocarbonyl) pyridinium hydrogen L-malate. *J. Solid State Chem.* **246**, 237-244 (2017).

5. Bryndal, I. et. al. Comprehensive physicochemical studies of a new hybrid material: 2-Amino-4-methyl-3-nitropyridinium hydrogen oxalate. *Spectrochim. Acta, Part A.* **117**, 434-441 (2014).

6. Thanigaimani, K., Khalib, N. C., Farhadikoutenaei, A., Arshad, S. & Razak, I. A. Synthesis and structural characterization of two new charge-assisted hydrogen-bonded supramolecular networks in 2-amino-4-methylpyridinium isophthalate dihydrate and 2-amino-5-methylpyridinium hydrogen isophthalate. *Mol. Cryst. Liq. Cryst.* **606**, 246-261 (2015).

7. Dauengauer, S. A., Sazanov, Y. N., Shibaev, L. A., Bulina, T. M. & Stepanov, N. G. Complexes of acid amides with polar aprotic solvents. II. Thermal analysis of the complexes of bis(N-phenyl)-pyromellitic acid amide with dimethylformamide,

dimethylacetamide, N-methylpyrrolidone and dimethylsulfoxide. *J. Therm. Anal.* **25**, 441-447 (1982).
