# Supplementary material for: Designed wrinkles for optical encryption and flexible integrated circuit carrier board
Source: Nat Commun. 2024 Jul 4;15:5616. doi: 10.1038/s41467-024-50069-7 (PMC11224375; doi:10.1038/s41467-024-50069-7)
Supplement: Supplementary file 2 — Description of Supplementary Information [file 41467_2024_50069_MOESM2_ESM.docx]

**Description of Additional Supplementary Files**

File Name: Supplementary Movie 1

Description: MJ image encrypted with wrinkled dot matrix

File Name: Supplementary Movie 2

Description: Switchable images of 4 numbers

File Name: Supplementary Movie 3

Description: Switchable images of 8 numbers

File Name: Supplementary Movie 4

Description: Adhesion stability of copper lines on wrinkled polyimide

File Name: Supplementary Movie 5

Description: High conductivity of copper circuit on wrinkled polyimide

File Name: Supplementary Movie 6

Description: High reliability of copper circuit on wrinkled polyimide
